# Ruthenium-Catalyzed Transfer Hydrogenation of Alkynes: Access to Alkanes and (*E*)- or (*Z*)-Alkenes in Tandem with Pd/Cu Sonogashira Cross-Coupling

**DOI:** 10.1021/acs.joc.4c01864

**Published:** 2025-02-27

**Authors:** Dominik Jankovič, Janez Košmrlj, Martin Gazvoda

**Affiliations:** University of Ljubljana, Faculty of Chemistry and Chemical Technology, Večna pot 113, SI-1000 Ljubljana, Slovenia

## Abstract

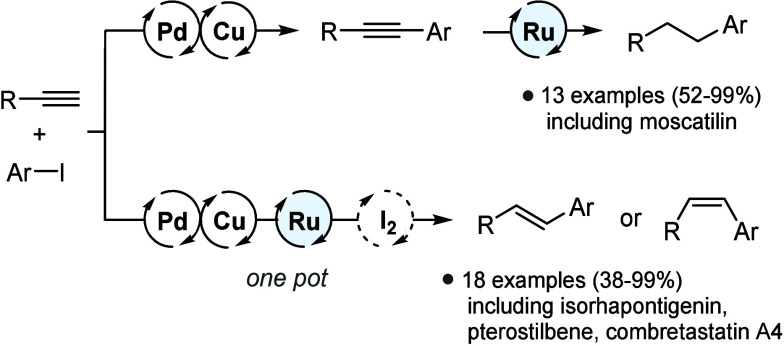

A complete reduction of alkynes using a combination of [RuCl_2_(*p*-cymene)]_2_, DTBM-SEGPHOS, and a paraformaldehyde/water system as the hydrogen source was developed, affording alkanes in 52–99% yields. In addition, the preparation of alkenes from terminal alkynes and aryl iodides by a tandem process of Pd/Cu-catalyzed Sonogashira reaction followed by Ru-catalyzed transfer hydrogenation is reported, affording alkenes in 38–99% yields. This multicatalysis proceeds via three consecutive reactions and can be extended further, as shown by adding an iodine-catalyzed *cis*–*trans* alkene isomerization step.

Catalytic hydrogenation reactions are among the most studied transformations with wide-ranging applications.^1^ With regard to the hydrogen source, the reactions can be divided into (i) those employing hydrogen gas, usually carried out in pressurized reaction vessels with heterogeneous palladium catalysts,^2^ and (ii) transfer hydrogenation reactions, in which the hydrogen is formed *in situ* from the reagent(s) and transferred to the substrate by the homogeneous catalyst, often ruthenium.^3^ Although ruthenium transfer hydrogenation has recently been extended to the partial hydrogenation of alkynes to either (*Z*)- or (*E*)-alkenes,^4^ no such reaction exists for the efficient complete hydrogenation of alkynes to alkanes. To date, only two such homogeneous transfer hydrogenation reactions catalyzed by copper^5^ and iridium^6^ have been described.

Alkynes, routinely prepared by the Sonogashira cross-coupling reaction, are substrates for subsequent partial hydrogenation to alkenes,^4^ which are important scaffolds in medicinal chemistry,^7^ commonly found in numerous naturally occurring substances,^8^ including the tubulin polymerization inhibitor combretastatin A4, an anticancer drug candidate that has already undergone several clinical trials.^9^

Via combination of the robustness and versatility of Sonogashira cross-coupling and ruthenium-catalyzed transfer hydrogenation, the two reactions could be combined into a powerful multicatalytic one-pot process that enables the direct production of (*E*)- or (*Z*)-alkenes starting from aryl halides and terminal alkynes (Figure 1). So far, only a few similar one-pot multi-metal-based transformations have been reported.^10^

**Figure 1:**
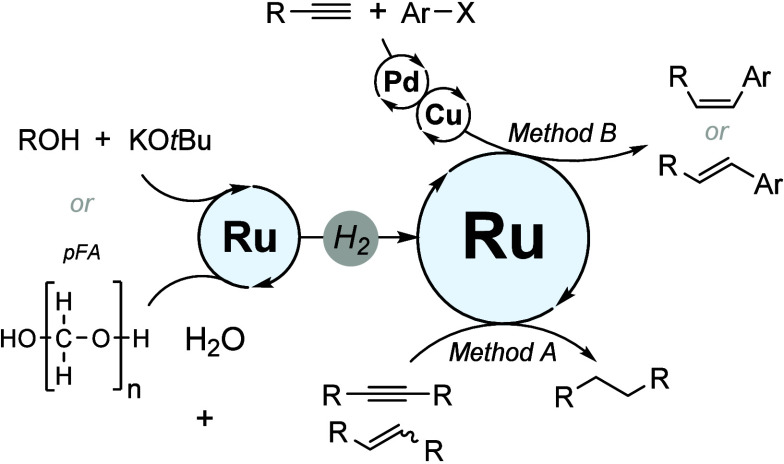
Development of ruthenium-catalyzed hydrogen generation with subsequent ruthenium-catalyzed transfer hydrogenation of alkynes and alkenes. In method A, hydrogen is produced from paraformaldehyde (pFA) and water and used for the hydrogenation of alkynes and alkenes in a stand-alone reaction. In method B, hydrogen is generated from alcohol (ROH)/KO*t*Bu, with the hydrogenation of the alkynes taking place in parallel to the Sonogashira cross-coupling.

Multicatalytic one-pot reactions are also an important area for the development of more sustainable synthetic chemistry.^11^

Reviewing the literature on the ruthenium-catalyzed transfer hydrogenation of alkynes,^4^ we envisioned a protocol for a simple complete hydrogenation of alkynes using standard laboratory equipment and commercially available catalysts and reagents. While the full hydrogenation of alkynes to alkanes is commonly accomplished using heterogeneous Pd/C catalysis, employing a transfer hydrogenation method eliminates the need for high-pressure hydrogen reactors. Although the complete hydrogenation of diphenylacetylene was reported by the groups of Prechtl,^4b^ Kann,^4c^ and Gelman,^4d^ there has been no general ruthenium-based method for the full hydrogenation of alkynes to alkanes. Inspired by the literature^12^ and our preliminary results, we identified great potential in the combination of [RuCl_2_(*p*-cymene)]_2_ and a paraformaldehyde (pFA)/water system for hydrogen formation and subsequent alkyne hydrogenation.^13^

In transition-metal catalysis, the ligand plays an important role.^14^ When screening different ruthenium and hydrogen sources, we found that the presence of a bidentate phosphine ligand is crucial for the formation of a fully reduced product. In a model reaction, the hydrogenation of diphenylacetylene (Figure 2), we investigated 11 ligands of this category, which differ in their bite angles and electronic properties. Ligands with three aryl substituents on the phosphine atom, e.g., BINAP, (*S*)-SEGPHOS, and (*S*)-DTBM-SEGPHOS, proved to be the best for the conversion under investigation. The optimization process and the results can be found in the Supporting Information. While the complete reduction proceeded efficiently at a lower reaction temperatures [80 °C (Figure 2, entry 13)], an increased temperature was later used to screen substrates in toluene to minimize the formation of alkene byproducts. It is noteworthy that the water rich solvent system (2:1 toluene:water ratio), which also serves as a reagent for Ru-catalyzed formation of hydrogen from pFA, was advantageous for the studied transformation, as such a system was previously shown to be efficient for hydrogen solubility and/or storage.^12b^

**Figure 2:**
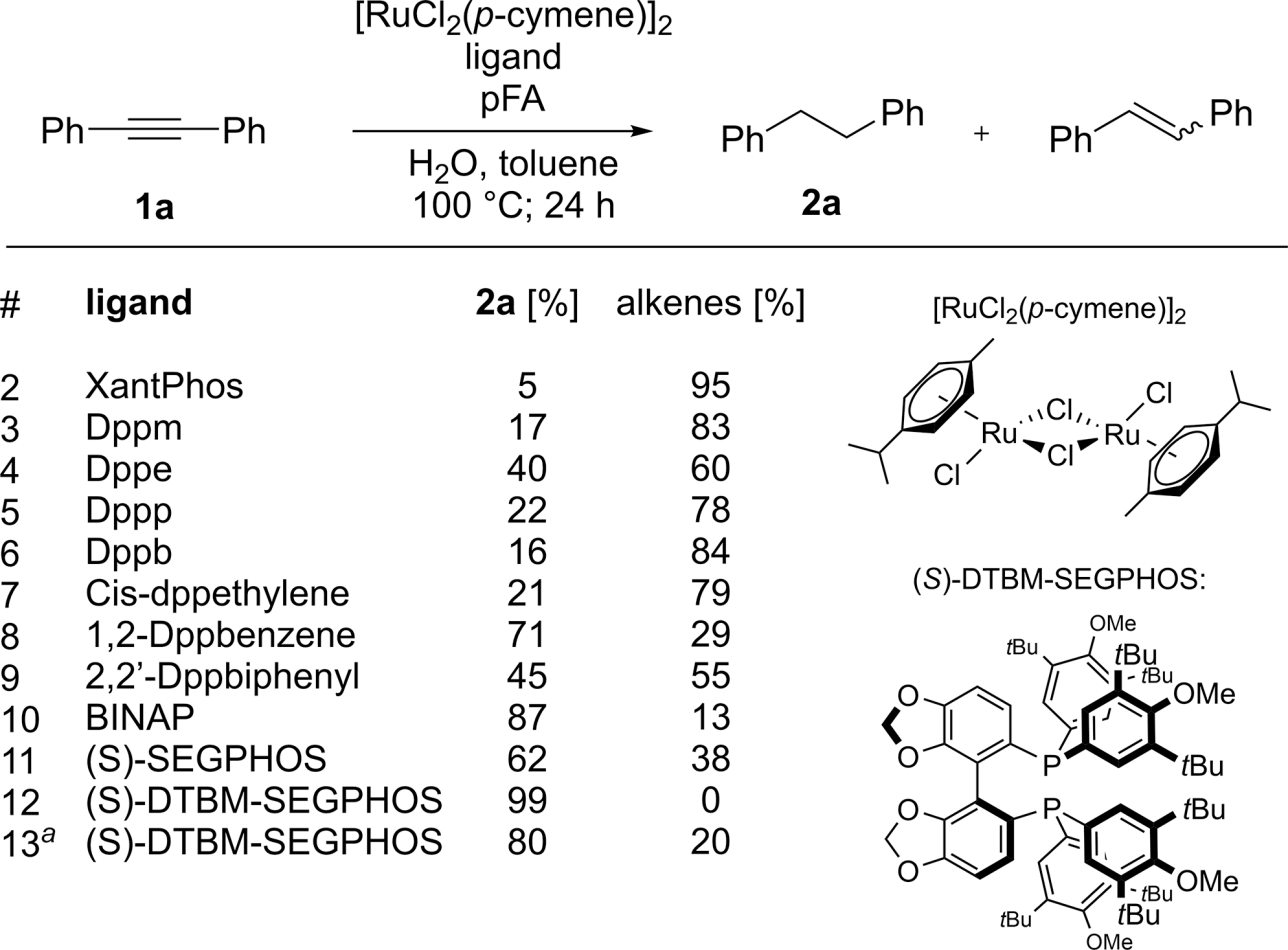
Optimization of the catalytic system for the full hydrogenation of internal alkynes. Reaction conditions: **1a** (0.5 mmol), [RuCl_2_(*p*-cymene)]_2_ (2.5 mol %), a ligand (5 mol %), pFA (5 mmol), toluene (2 mL), and H_2_O (1 mL). Conversion was determined by ^1^H NMR spectroscopy using 1,3,5-trimethoxybenzene as an internal standard. ^*a*^At 80 °C.

We tested the developed protocol on various internal alkynes (Figure 3) and generated 15 alkane products with moderate to excellent isolated yields. The hydrogenation of internal alkynes with electron-withdrawing functional groups (**1h**–**1j**) proceeded with conversions higher than those with electron-donating groups (**1f** and **1n**). Product **2d** was prepared from the corresponding internal alkyne with a ketone fragment, which was reduced in conjunction with the C≡C bond, giving **2d** in 82% yield in racemic form, despite the use of a chiral ligand.^15^ The aldehyde in **1k** was reduced to alcohol **2k**, while the nitro and ester functional groups in **1h**, **1j**, **1l**, and **1m** were retained. Using deuterated paraformaldehyde (pFA-*d*_2_) in combination with D_2_O, alkynes could be reduced to deuterated products, as shown in the case of **2m**. The developed method was used for the preparation of the natural product moscatilin (**2n**).^16^

**Figure 3:**
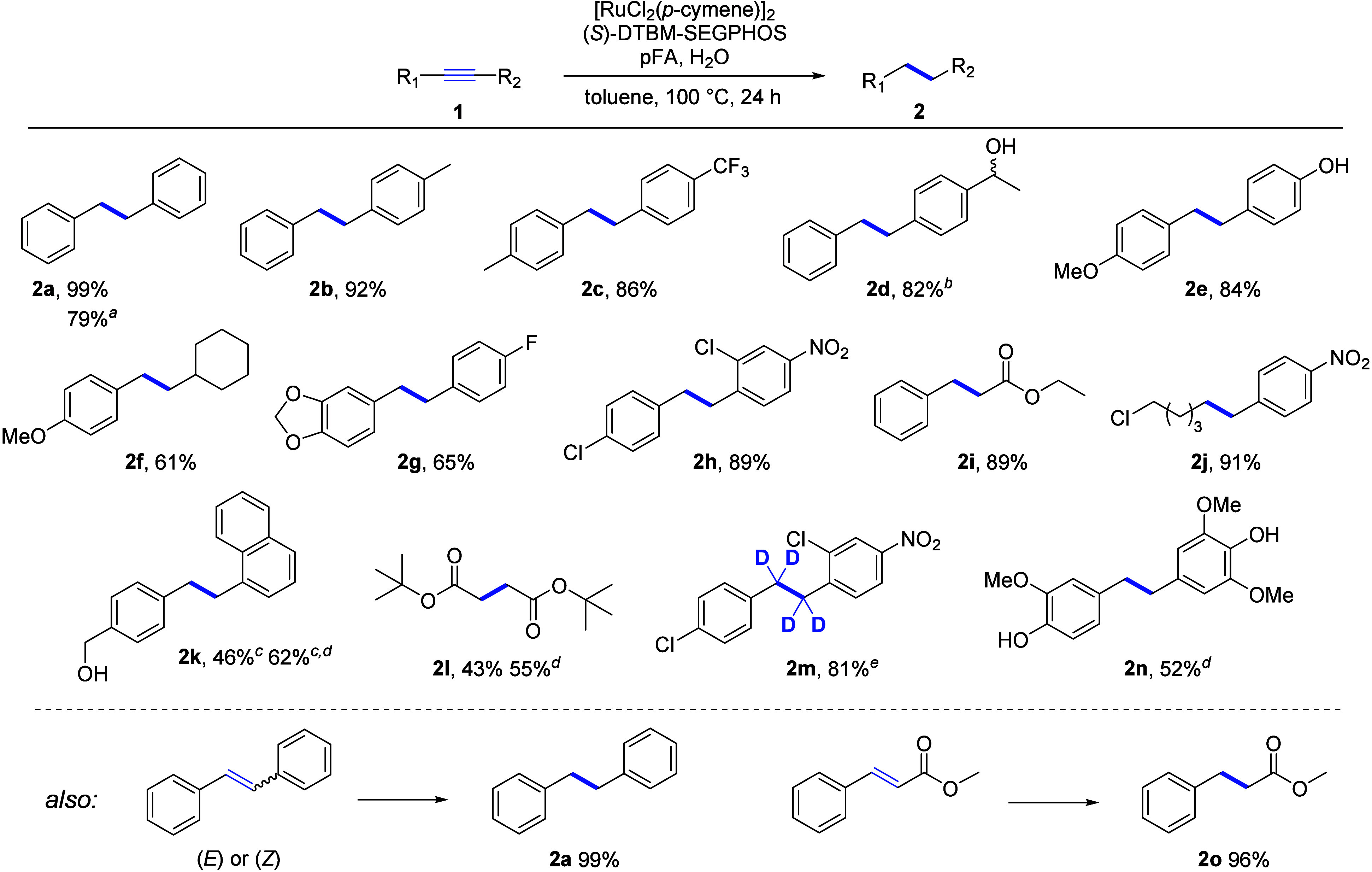
Substrate scope of the developed full hydrogenation of internal alkynes. Reaction conditions: internal alkyne (0.5 mmol, 1 equiv), [RuCl_2_(*p*-cymene)]_2_ (2.5 mol %, 5 mol % total Ru), (*S*)-DTBM-SEGPHOS (0.025 mmol, 5 mol %), pFA (5 mmol, 10 equiv), toluene (2 mL), and H_2_O (1 mL). Yields after purification by column chromatography are given. ^*a*^On a 2.0 mmol scale. ^*b*^The ketone functionality was reduced to alcohol alongside the C≡C bond. ^*c*^The aldehyde functionality was reduced to alcohol alongside the C≡C bond. ^*d*^Benzene was used instead of toluene. ^*e*^Deuterated paraformaldehyde (pFA-*d*_2_) and D_2_O were used.

Because the reaction proceeds via semireduced alkene intermediates, the method can also be used for the hydrogenation of alkenes, which is the more difficult of the two reduction steps that occur during the process. This was demonstrated in reactions with (*E*)- and (*Z*)-stilbene, both forming **2a** with quantitative conversion, and in the synthesis of **2o**, which was prepared from (*E*)-methyl cinnamate in 96% yield.

As a complement to the complete reduction of alkynes described above, we have developed a one-pot protocol based on Pd/Cu Sonogashira coupling followed by Ru-catalyzed transfer hydrogenation to the desired (*E*)- or (*Z*)-alkenes using only commercially available and inexpensive reagents, i.e., terminal alkynes and aryl iodides. For the Ru-catalyzed reduction step, we replaced the pFA/H_2_O hydrogen source, which is incompatible with the Sonogashira cross-coupling, with the Ru_3_(CO)_12_/alcohol/KO*t*Bu system previously described by Ekeberg et al.^4c^ Initially, we optimized the reaction between phenylacetylene (**3a**) and 4-iodotoluene (**4a**) as model substrates to selectively form (*E*)-alkene by adding a catalytic amount of iodine.^17^ However, this protocol proved to be ineffective when tested with a wider range of substrates, as the Sonogashira couplings performed poorly under these conditions. The use of triethylamine as both a base and a solvent proved to be advantageous, and the replacement of the Ru_3_(CO)_12_ with Grubbs catalyst M102 eliminated the need to use iodine to achieve (*E*)-selective conversion, most likely due to the different coordination sphere in Ru (Figure 4).

**Figure 4:**
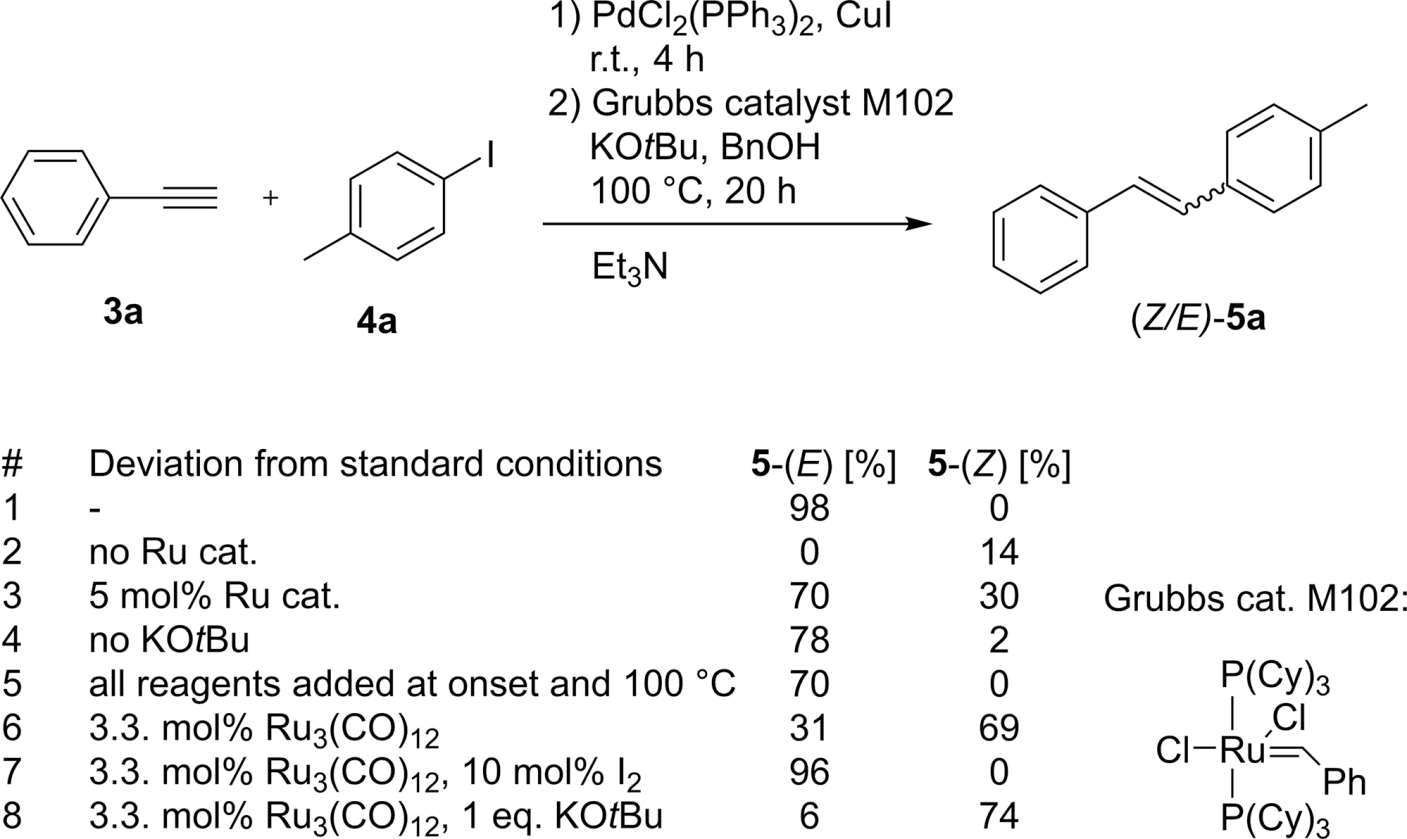
Development of a one-pot protocol for sequential Sonogashira cross-coupling and Ru-catalyzed hydrogenation. Standard conditions: 4-iodotoluene (0.5 mmol), phenylacetylene (0.6 mmol), PdCl_2_(PPh_3_)_2_ (4 mol %), CuI (5 mol %), triethylamine (3 mL), 4 h at room temperature; then Grubbs catalyst M102 (10 mol %), KO*t*Bu (1 mmol), BnOH (5 mmol), and 20 h at 100 °C. Conversions to (*E*)- and (Z)-alkenes were determined by ^1^H NMR.

Application of the developed protocol (Figure 4, entry 1) by addition of the Ru catalyst to the reaction mixture after completion of the Sonogashira coupling leads to (*E*)-stilbene in 98% overall conversion. Combining all reagents at the beginning and heating the reaction mixture to 100 °C overnight also led to (*E*)-stilbene as the main product, albeit with a slightly lower conversion of 70% (Figure 4, entry 5). This shows that Pd/Cu Sonogashira cross-coupling and ruthenium-catalyzed transfer hydrogenation can proceed concurrently. In the absence (Figure 4, entry 2) or in the presence of a smaller amount (Figure 4, entry 3) of the ruthenium catalyst, a minor conversion to (*Z*)-stilbene or a mixture of (*E*)- and (*Z*)-stilbenes, respectively, was observed. When Ru_3_(CO)_12_ was used (Figure 4, entry 6) or in combination with a smaller amount of KO*t*Bu (Figure 4, entry 8), a reversal of the stereoselectivity was observed, with (*Z*)-stilbene being formed as the major product. The selectivity for (*E*)-stilbene with the Ru_3_(CO)_12_ catalyst can be increased by the addition of 10 mol % molecular iodine in the second part of the reaction (Figure 4, entry 7), arguably extending the tandem sequence by the fourth catalytic transformation, i.e., Sonogashira coupling, Ru-catalyzed hydrogen formation, Ru-catalyzed reduction, and iodine-catalyzed alkene isomerization.^18^

Despite the fact that the complex reaction mixture contains several reagents, the developed tandem protocol worked surprisingly well when tested with different substrates (Figure 5). Eighteen products in the (*E*)-configuration were isolated in moderate to good yields, with minute amounts (≤10%) of (*Z*)-alkenes formed in the cases of **5b**, **5e**, **5f**, **5i**, **5k**, and **5p**, which were easily separated by column chromatography. The protocol gave poor conversions to **5** when the substrates contained nitro or ketone functions. Simultaneous dehalogenation occurred in **5j** when using 4-bromoiodobenzene, whereas no dehalogenation was observed with iodochlorobenzene in **5g**. Using the developed method, two naturally occurring stilbenes, namely, pterostilbene^19^**5b** and isorhapontigenin^20^**5i**, were prepared in one step in 64% and 60% isolated yields, respectively. We prepared (*E*)-combretastatin A4 **5p** and medicinally relevant (*Z*)-combretastatin A4 **5s** in 57% isolated yield using (*Z*)-selective reaction conditions.

**Figure 5:**
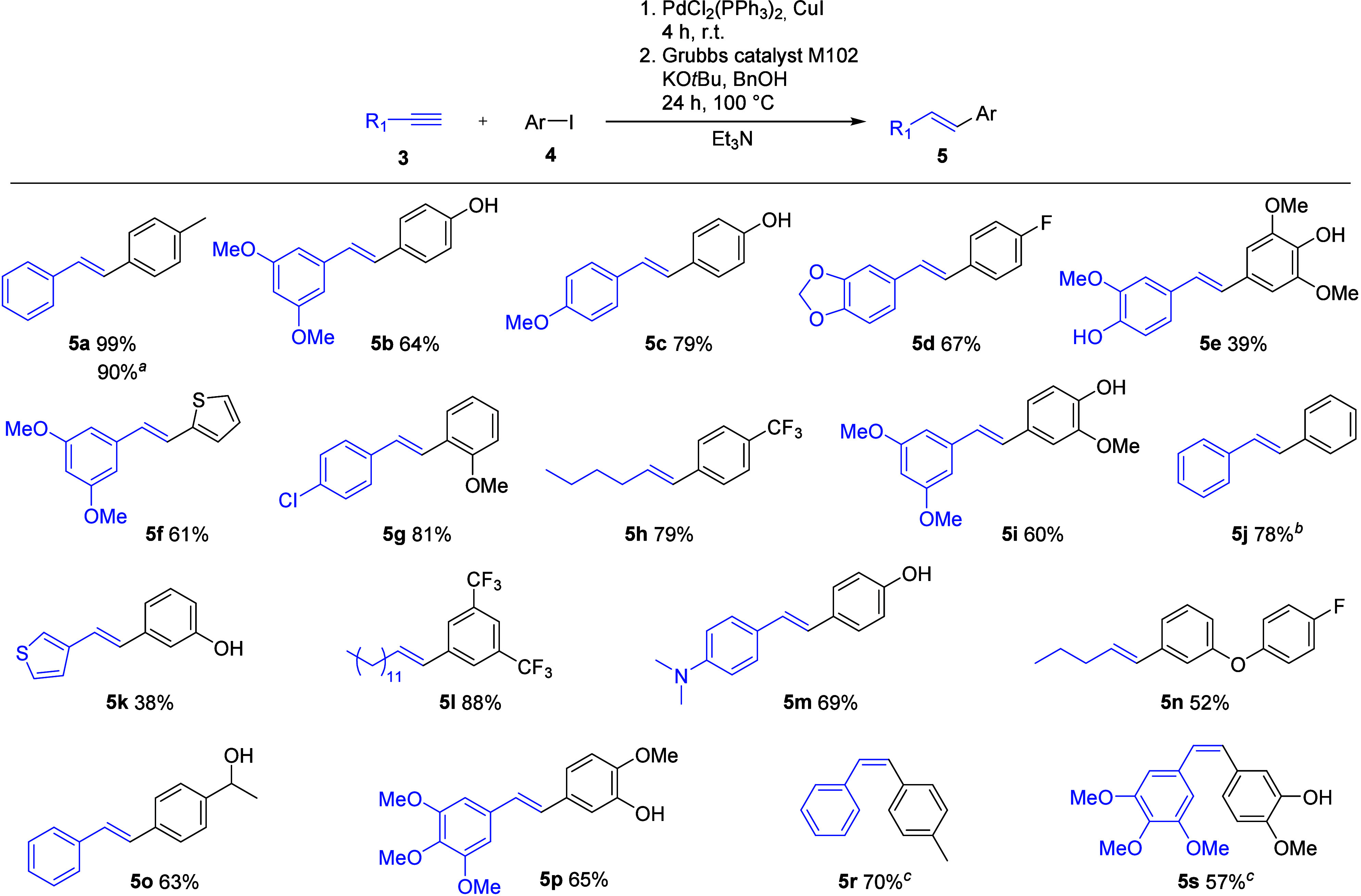
Substrate scope of the sequential Sonogashira cross-coupling–hydrogenation reaction. Reaction conditions: aryl iodide **3** (0.5 mmol, 1 equiv), terminal alkyne **4** (0.6 mmol, 1.2 equiv), PdCl_2_(PPh_3_)_3_ (4 mol %), CuI (5 mol %), and Et_3_N (3 mL) stirred under a nitrogen atmosphere at room temperature for 4 h; then addition of Grubbs catalyst M102 (10 mol %), KO*t*Bu (1 mmol, 2 equiv), and BnOH (5 mmol, 10 equiv) and stirring under a nitrogen atmosphere for 20 h at 100 °C. The yields of the isolated products after column chromatography are given. ^*a*^On a 2.0 mmol scale. ^*b*^4-Iodobromobenzene used. ^*c*^Reaction conditions for (*E*)-selective reduction were employed, i.e., 3.33 mol % Ru_3_(CO)_12_ and KO*t*Bu (0.5 equiv).

In summary, we have developed simple protocols for the complete and partial hydrogenation of alkynes using standard laboratory equipment and commercially available catalysts and reagents. The methods developed are not based on heterogeneous palladium-based catalysis and do not require hydrogen gas or special reaction vessels. The combination of reliable Sonogashira cross-coupling for the preparation of internal alkynes in conjunction with ruthenium catalysis enables the construction of C(sp^3^)–C(sp^3^) and C(sp^2^)–C(sp^2^) motifs directly for aryl iodides and terminal alkynes, an alternative to direct coupling methods and stepwise protocols for the preparation of alkanes and alkenes. The tandem process for the preparation of alkenes, which proceeds via three successive catalytic reactions and uses several reagents in the reaction mixture, can, as we have shown, be extended by additional alkene isomerization and is therefore modular for future investigations.

## Data Availability

The data underlying this study are available in the published article and its Supporting Information.

## Supporting Information Available

A detailed description of experimental procedures and optimization protocols, compound characterization data, and copies of ^1^H and ^13^C NMR spectra (PDF)
