# Supplementary material for: Ruthenium-Catalyzed Transfer Hydrogenation of Alkynes: Access to Alkanes and (E)- or (Z)-Alkenes in Tandem with Pd/Cu Sonogashira Cross-Coupling
Source: J Org Chem. 2025 Feb 27;90(9):3480–4. doi: 10.1021/acs.joc.4c01864 (PMC11894645; doi:10.1021/acs.joc.4c01864)
Supplement: Supplementary file 1 — jo4c01864_si_001.pdf [file jo4c01864_si_001.pdf]

SUPPORTING INFORMATION

Ruthenium-Catalyzed Transfer Hydrogenation of Alkynes: Access to Alkanes and (*E*)- or (*Z*)-Alkenes in Tandem with Pd/Cu Sonogashira Cross-Coupling

Dominik Jankovič, Janez Košmrlj, Martin Gazvoda*

University of Ljubljana, Faculty of Chemistry and Chemical Technology, Večna pot 113, SI-1000 Ljubljana, Slovenia

e-mail: martin.gazvoda@fkkt.uni-lj.si

TABLE OF CONTENT

1. GENERAL INFORMATION S1

1.2. General reagent information S1

1.3. General experimental information S1

1.4. General analytical information........ S2

2. EXPERIMENTAL PROCEDURES AND CHARACTERIZATION DATA S3

2.1. Optimization of full hydrogenation of internal alkynes S3

2.2. Optimization of tandem one-pot partial hydrogenation coupled with Sonogashira cross-coupling in toluene........ S5

2.3. Optimization of sequential one-pot partial hydrogenation coupled with Sonogashira cross coupling in triethylamine S7

2.4. Substrate scope of full hydrogenation........ S8

2.5. Substrate scope of sequential one-pot partial hydrogenation coupled with Sonogashira cross coupling........ S17

2.6. Synthesis of internal alkynes........ S25

2.7. Synthesis of other precursors S30

3. COPIES OF NMR SPECTRA........ S35

4. REFERENCES........ S70

1. GENERAL INFORMATION

1.2. General reagent information

Anhydrous solvents were purchased from Sigma-Aldrich. Other work-up solvents were purchased from Honeywell, Fluorochem, Carlo Erba, J. T. Baker and Fisher Scientific. Deuterated $CDCl_3$ and DMSO-*d*$_6$ were purchased from Euriso-top and were used without further purification. Reagents, ligands and metal sources were obtained from commercial sources (Sigma-Aldrich, TCI, Doug Discovery, BLDPharm).

1.3. General experimental information

Caution! Internal pressurre of hydrogen builds up during the full hydrogenation reactions.

In full hydrogenation reactions of alkynes to alkanes using pFA/H_2O/Ru system pressure builds up in the reaction tube due to hydrogen generation. After the reaction, the reaction mixture is cooled to room temperature before the tube is opened and pressure is released. No incidents occured during experimental work, however caution is required.

Complete hydrogenation reactions of alkynes to alkanes at 0.5 mmol scale were performed in Fisherbrand Borosilicate Glass Tubes (Fisherbrand™ FS73750-13100) with black polypropylene screw cap, 8.5mm center hole, 13mm diameter, with red PTFE/white silicone/red PTFE septum (cat. no. TSH-CV1946 from Premium vials) or Ace pressure tubes (Sigma Aldrich Z564559), however we recommend using the latter. The 2 mmol scale reactions were performed in an Ace pressure tube with PTFE cap (Sigma Aldrich Z564621).

No precaution was taken to exclude air. The reactions were heated in an oil bath, preheated to the specified temperature.

Sequential one-pot reactions from iodo arenes and terminal alkynes on 0.5 or 2.0 mmol scale were performed in oven-dried 10 mL or 100 mL round-bottom flasks under argon atmosphere, respectively. Rubber septum and nitrogen balloon were used for first 4 hours of the reaction at room temperature. Greased glass joint attached with metal clamp was used for the subsequent hydrogenation reaction. The reactions were heated in an oil bath, preheated to the specified temperature.

Column chromatography was performed using Interchim puriFlash XS520 Plus automated system with petroleum ether/ethyl acetate gradient elution using Silica gel columns (15 µm, 40 g SiO_2). Elution programme was determined by software based on TLC retention factors. TLC analyses were performed using TLC Silica gel 60 F_{254} plates using petroleum ether/ethyl acetate mobile phases in rations 1/1, 3/1, 5/1 and 10/1.

1.4. General analytical information

NMR spectra were recorded with Bruker Avance III 500 MHz NMR instrument at 296 K operating at 500 MHz (^{1}H) and 126 MHz (^{13}C{^{1}H}) and Bruker Avance NEO 600 MHz at 296 K operating at 600 MHz (^{1}H) and 150 MHz (^{13}C{^{1}H}). Proton spectra were referenced to residual $CHCl_3$ in deuterated chloroform (δ = 7.26 ppm) and DMSO-d_5 in DMSO-d_6. Carbon spectra were referenced to the central line of ^{13}C signal of $CDCl_3$ (δ = 77.2 ppm) in deuterated chloroform and $(CD_3)_2SO$ (δ = 39.52 ppm) in DMSO-d_6. Chemical shifts (δ) are given in ppm. Coupling constants are given in Hz. Multiplicities are indicated as: s (singlet), d (doublet), t (triplet), q (quartet), m (multiplet) and br (broadened).

High resolution mass spectra (HRMS) were recorded on time-of-flight Agilent Accurate Mass TOF LC/MS spectrometer (Agilent 6224) equipped with a double orthogonal electrospray source at atmospheric pressure ionization (ESI+).

Chiral analysis of product **2d** was preformed with Agilent Infinity 1260 II HPLC system using Chiralcel AD-H column (4.6 x 250 mm, 5µm), 25 °C column temperature. Isocratic elution: 95/5; hexane/isopropanol, 1 mL/min flow rate, detection at UV 220 nm, 1 µL injection volume of sample (1 mg/mL in acetonitrile).

2. EXPERIMENTAL PROCEDURES AND CHARACTERIZATION DATA

2.1. Optimization of full hydrogenation of internal alkynes to alkanes

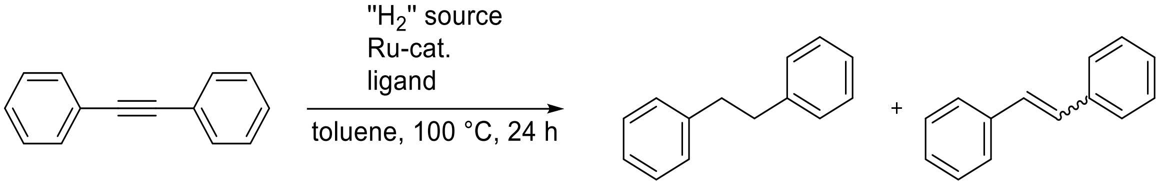

Into a screw-capped glass tube were added in the following order: diphenylacetylene (89 mg, 0.5 mmol, 1 equiv.), ruthenium catalyst, ligand, toluene (2 mL) and hydrogen source. Glass tube was tightly closed with a screw cap and the reaction mixture was stirred in an oil bath at 100 °C for 24 hours. No precaution was taken to exclude air. Then, the reaction tube was taken from the oil bath and was cooled to room temperature to decrease the internal pressure, opened and the reaction mixture was filtered through a pad of SiO_2 using ethyl acetate as an eluent. Into filtrate defined abount of 1,3,5-trimethoxybenzene (TMB) was added and dissolved, an aliquote was taken from the solution and solvent was removed under reduced pressure using rotary evaporator. The aliquote was dissolved in $CDCl_3$ and subjected to ^{1}H NMR analysis. The conversion to desired product was determined by comparing characteristic proton resonance of 1,3,5-trimethoxybenzene (TMB) at δ 6.08 ppm, s, 3H with proton resonance of bibenzyl resonance at δ 2.92 ppm, s, 4H, (*E*)-olefin resonance at δ 7.11 ppm, s, 2H and (*Z*)-olefin resonance at δ 6.59 ppm, s, (2H). The conversion to the olefin product is given as a sum of *E*- and *Z*-isomer.

Supporting Table S1: Optimization of full hydrogenation of internal alkynes

Entry	"H_2" source [equiv.]	Ru cat. [mol% of Ru]	Ligand [mol%]	Conversion to bibenzyl [%]	Conversion to olefin [%]
1	1-methoxy-2-propanol [20] + KOtBu [2]	$Ru(H_2)(CO)(PPh_3)_3$ [10]	/	0	100
2	1-methoxy-2-propanol [20] + KOtBu [2]	$Ru_3(CO)_{12}$ [15]	/	55	30
3	1-methoxy-2-propanol [20] + KOtBu [2]	$Ru_3(CO)_{12}$ [15]	PCy_3 [30]	1	99
4	1-methoxy-2-propanol [20] + KOtBu [2]	Grubbs catalyst [5]	/	0	41
5	*i*PrOH [20] + KOtBu [2]	Grubbs catalyst [5]	/	26	68
6	MeOH [20] + KOtBu [2]	Grubbs catalyst [5]	/	4	96
7	Ethylene glycol [10] + KOtBu [2]	Grubbs catalyst [5]	/	3	92

8	Ethylene glycol [10] + KOtBu [2]	$Ru_3(CO)_{12}$ [5]	/	5	50
9	1-methoxy-2-propanol [20] + K_2CO_3 [2]	$Ru_3(CO)_{12}$ [15]	/	0	92
10	Glycerol [20] + KOtBu [2]	$Ru_3(CO)_{12}$ [15]	/	3	88
11	1-phenoxy-2-propanol [20] + KOtBu [2]	$Ru_3(CO)_{12}$ [15]	/	12	86
12	BnOH [20]	$Ru_3(CO)_{12}$ [10]	Dppf [10]	8	92
13	pFA [10] + 1mL H_2O + KOtBu [2]	Grubbs catalyst [5]	/	3	97
14	pFA [10] + 1mL H_2O	$Ru_3(CO)_{12}$ [5]	/	0	82
15	pFA [10] + 1mL H_2O	$[RuCl_2(p\text{-}cy)]_2$ [5]	PCy_3 [5]	15	80
16	pFA [10] + 1mL H_2O + KOtBu [2]	$[RuCl_2(p\text{-}cy)]_2$ [5]	Dppf [5]	3	70
17	pFA [10] + 1mL H_2O + KOtBu [2]	$[RuCl_2(p\text{-}cy)]_2$ [5]	DavePhos [5]	16	84
18	pFA [10] + 1mL H_2O	$[RuCl_2(p\text{-}cy)]_2$ [5]	BINAP [5]	87	13
19	pFA [10] + 1mL H_2O + KOtBu [2]	$[RuCl_2(p\text{-}cy)]_2$ [5]	BINAP [5]	19	81
20	pFA [10] + 1mL H_2O	$[RuCl_2(p\text{-}cy)]_2$ [5]	XantPhos [5]	5	95
21	pFA [10] + 1mL H_2O	$[RuCl_2(p\text{-}cy)]_2$ [10]	BINAP [10]	35	65
22	pFA [10] + 1mL H_2O	$Ru_3(CO)_{12}$ [5]	BINAP [5]	0	57
23	pFA [10] + 1mL H_2O	$RuCl_3$ [5]	BINAP [5]	0	24
24	pFA [10] + 1mL H_2O	$RuCp(PPh_3)_2Cl$ [5]	BINAP [5]	0	26
25	pFA [10] + 1mL H_2O	$RuCp^*(PPh_3)Cl$ [5]	BINAP [5]	0	58
26	37% formaldehyde solution [10]	$[RuCl_2(p\text{-}cy)]_2$ [5]	BINAP [5]	10	80
27	pFA [10]	$[RuCl_2(p\text{-}cy)]_2$ [5]	BINAP [5]	0	4
28	pFA [10] + 1mL H_2O + KOH [2]	$[RuCl_2(p\text{-}cy)]_2$ [5]	BINAP [5]	4	96
29	pFA [20] + 1 mL H_2O	$[RuCl_2(p\text{-}cy)]_2$ [5]	BINAP [5]	52	48
30	pFA [10] + 1 mL H_2O	$[RuCl_2(p\text{-}cy)]_2$ [5]	iPr.HCl [5]	66	34
31	pFA [10] + 1 mL H_2O	$[RuCl_2(p\text{-}cy)]_2$ [5]	1,2-dppbenzene [5]	71	29
32	pFA [10] + 1 mL H_2O	$[RuCl_2(p\text{-}cy)]_2$ [5]	Dppm [5]	17	83
33	pFA [10] + 1 mL H_2O	$[RuCl_2(p\text{-}cy)]_2$ [5]	Dppe [5]	40	60

34	pFA [10] + 1 mL H_2O	$[RuCl_2(p\text{-}cy)]_2$ [5]	*Cis*-dppethylene [5]	21	79
35	pFA [10] + 1 mL H_2O	$[RuCl_2(p\text{-}cy)]_2$ [5]	(S)-SEGPHOS [5]	62	38
36	pFA [10] + 1 mL H_2O	$[RuCl_2(p\text{-}cy)]_2$ [5]	Dppp [5]	22	78
37	pFA [10] + 1 mL H_2O	$[RuCl_2(p\text{-}cy)]_2$ [5]	Dppb [5]	16	84
38	pFA [10] + 1 mL H_2O	$[RuCl_2(p\text{-}cy)]_2$ [5]	2,2'-dppbiphenyl [5]	45	55
**39**	**pFA [10] + 1 mL H_2O**	**$[RuCl_2(p\text{-}cy)]_2$ [5]**	**(S)-DTBM-SEGPHOS [5]**	**100**	**0**
**40[a]**	**pFA [10] + 1 mL H_2O**	**$[RuCl_2(p\text{-}cy)]_2$ [5]**	**(S)-DTBM-SEGPHOS [5]**	**100**	**0**
41[b]	pFA [10] + 1 mL H_2O	$[RuCl_2(p\text{-}cy)]_2$ [5]	(S)-DTBM-SEGPHOS [5]	80	20

[a]Benzene used as solvent, [b]80 °C reaction temperature. iPr.HCl = 1,3-Bis-(2,6-diisopropylphenyl)imidazolinium chloride, Dppf = 1,1′-Ferrocenediyl-bis(diphenylphosphine), Dppm = Bis(diphenylphosphino)methane, Dppe = Bis(diphenylphosphino)ethane, Dppp = Bis(diphenylphosphino)propane, Dppb = Bis(diphenylphosphino)butane, *C*is-dppethylene = *cis*-1,2-Bis(diphenylphosphino)ethylene, 1,2'-Dppbenzene = 1,2-Bis(diphenylphosphino)benzene, 2,2'-Dppbiphenyl = 2,2'-Bis(diphenylphosphino)-1,1'-biphenyl.

2.2. Optimization of one-pot sequential Pd/Cu Sonogashira cross-coupling and Ru-catalyzed hydrogenation to alkenes in toluene

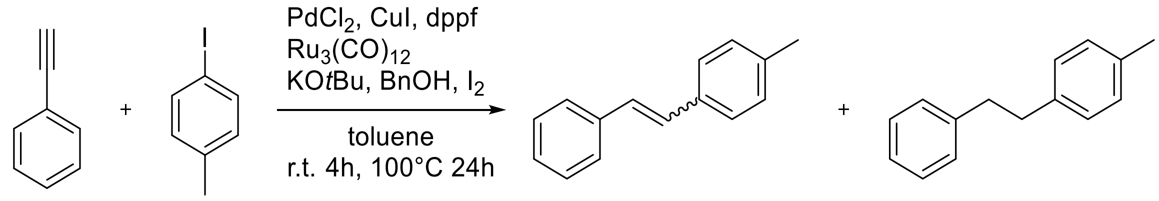

Oven-dried 10 mL round bottom reaction flask was equipped with a rubber septum and purged with nitrogen. Under nitrogen flow and stirring were added in the following order: dry toluene (2 mL), $PdCl_2$ (3.6 mg, 0.02 mmol, 4 mol%), CuI (4.8 mg, 0.025 mmol, 5 mol%), dppf (1,1'-bis(diphenylphosphino)ferrocene) (11 mg, 0.02 mmol, 4 mol%), $Ru_3(CO)_{12}$ (10.9 mg, 0.017 mmol, 3.33 mol%, total 10 mol% Ru), KO*t*Bu (112 mg, 1 mmol, 2 equiv.), I_2 (12.7 mg, 0.05 mmol, 10 mol%), 4-iodotoluene (109 mg, 0.5 mmol, 1 equiv.), phenylacetylene (61 mg, 66 µL, 0.6 mmol, 1.2 equiv.), and benzyl alcohol (521 mg, 499 µL, 5 mmol, 10 equiv.). The reaction flask was capped with a glass stopper and stirred at room temperature for 4 hours. After 4 hours, the reaction flask was placed into oil bath preheated to 100 °C. After 24 hours at 100 °C, the reaction mixture was cooled to room temperature,

the flask was opened and the reaction mixture was filtered through a pad of SiO_2 using dichloromethane as an eluent. A defined amount of 1,3,5-trimethoxybenzene (TMB) was added into the filtrate, TMB was dissolved, and an aliquote was taken from the solution, and solvent was removed under reduced pressure using rotary evaporator. The aliquote was dissolved in $CDCl_3$ and subjected to ^{1}H NMR analysis. The conversion to the products was determined by comparing characteristic proton resonance of TMB at δ 6.08 ppm, s, 3H, with the (*E*)-olefin resonance at δ 7.08 ppm, m, 2H, the (*Z*)-olefin resonance at δ 6.54 ppm, m, 2H, and the bibenzyl resonance at δ 2.92 ppm, s, 4H.

Supporting Table S2: Optimization of sequential one-pot protocol in toluene

Entry	Deviation from standard conditions	Conversion to *E*-olefin [%]	Conversion to *Z*-olefin [%]	Conversion to bibenzyl [%]
1	None	100	0	0
2	Without I_2	54	41	7
3	Without $Ru_3(CO)_{12}$	0	45	0
4	Direct heating to 100 °C for 24 hours	100	0	0
5	5 mol% $PdCl_2(PPh_3)_2$ and without dppf	94	0	0
6	1 mL of Et_3N instead of 2 equiv. KO*t*Bu	8	36	0
7	20 equiv. of BnOH	96	0	0
8	20 equiv. of BnOH and direct heating to 100 °C for 24 hours	95	0	0
9	20 equiv. of MeOH and direct heating to 100 °C for 24 hours	18	30	0
10	2 mol% $PdCl_2$, 3 mol% CuI, 2 mol% dppf, 20 equiv. of BnOH	87	0	0
11	2 mol% $PdCl_2$, 3 mol% CuI, 2 mol% dppf, 20 equiv. of 1-MeO-2-propanol and direct heating to 100 °C for 24 hours	75	2	7
12	2 mol% $PdCl_2(PPh_3)_2$, 3 mol% CuI, without dppf, 20 equiv. of 1-MeO-2-propanol, 4 hours at 40 °C then 100 °C for 20 hours	54	0	13
13	2 mol% $PdCl_2(PPh_3)_2$, 3 mol% CuI, without dppf, 20 equiv. of 1-MeO-2-propanol	63	0	7
14	2 mol% $PdCl_2(PPh_3)_2$, 3 mol% CuI, 10 mol% $Ru(H_2)(CO)(PPh_3)_3$, without dppf, 20 equiv. of 1-MeO-2-propanol	30	54	0

Standard conditions: phenylacetylene (1.2 equiv.), 4-iodotoluene (1 equiv.), $PdCl_2$ (4 mol%), CuI (5 mol%), dppf (4 mol%), $Ru_3(CO)_{12}$ (3.33 mol%, 10 mol% Ru), KO*t*Bu (2 equiv.), benzyl alcohol (10 equiv.), I_2 (10 mol%), toluene (2 mL). 4 Hours at room temperature then 20 hours at 100 °C.

2.3. Optimization of one-pot sequential Pd/Cu Sonogashira cross-coupling and Ru-catalyzed hydrogenation to alkenes in triethylamine

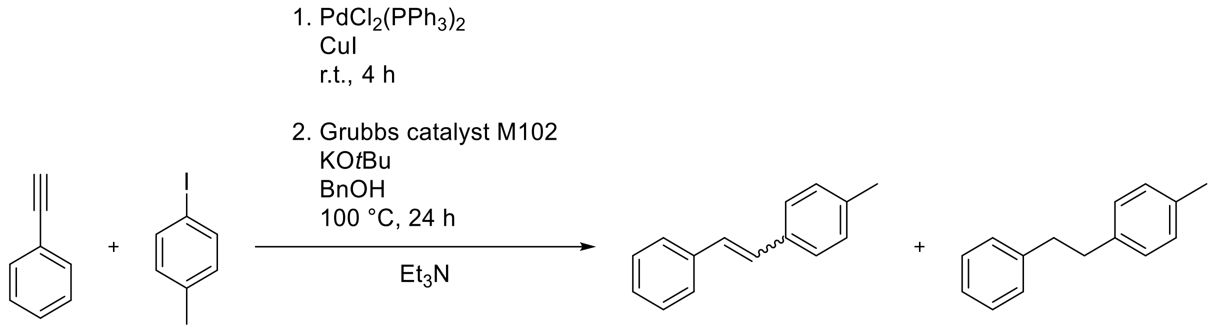

Oven-dried 10 mL round bottom reaction flask was equipped with a rubber septum and purged with nitrogen. Under nitrogen flow and stirring were added in the following order: anhydrous Et_3N (3 mL), $PdCl_2(PPh_3)_2$ (14 mg, 0.02 mmol, 4 mol%), CuI (4.8 mg, 0.025 mmol, 5 mol%), 4-iodotoluene (109 mg, 0.5 mmol, 1 equiv.), and phenylacetylene (61 mg, 66 µL, 0.6 mmol, 1.2 equiv.). Reaction flask was capped with septum equipped with balloon filled with nitrogen and the reaction mixture was stirred at room temperature for 4 hours. Then, under nitrogen flow were added in the following order: Grubbs catalyst M102 (41 mg, 0.05 mmol, 10 mol%), KO*t*Bu (112 mg, 1 mmol, 2 equiv.), and benzyl alcohol (521 mg, 499 µL, 5 mmol, 10 equiv.). The reaction flask was capped with a glass stopper and placed into a oil bath, pre-heated to 100 °C, and was stirred at for 24 hours. After 24 hours at 100 °C, the reaction flask was removed from oil bath and the reaction mixture was cooled to room temperature and opened. The reaction mixture was filtered through a pad of SiO_2 using dichloromethane as an eluent.

A defined amount of 1,3,5-trimethoxybenzene (TMB) was added into the filtrate, TMB was dissolved, an aliquote was taken from the solution, and solvent was removed under reduced pressure using rotary evaporator. The aliquote was dissolved in $CDCl_3$ and subjected to ^1H NMR analysis. The conversion to the products was determined by comparing characteristic proton resonance of TMB at δ 6.08 ppm, s, 3H, with the (*E*)-olefin resonance at δ 7.08 ppm, m, 2H, the (*Z*)-olefin resonance at δ 6.54 ppm, m, 2H, and the bibenzyl resonance at δ 2.92 ppm, s, 4H.

Supporting Table S3: Optimization of sequential one-pot protocol in triethylamine

Entry	Deviation from standard conditions	Conversion to E-olefin [%]	Conversion to Z-olefin [%]	Conversion to bibenzyl [%]
1	None	98	0	0
2	5 mol% of Ruthenium catalyst	70	30	0
3	All reagents added in the beginning and stirred at 100 °C for 24 hours	70	0	0
4	Without KO*t*Bu	78	2	0

5	Without Ruthenium catalyst	0	14	0
6	3.33 mol% of $Ru_3(CO)_{12}$, 1 equiv. KO*t*Bu and 10 mol% I_2 (all reagents were added in the begining, 4 hours at r.t., then 100 °C for 24 hours)	14	66	0
7	3.33 mol% of $Ru_3(CO)_{12}$	31	69	0
8	3.33 mol% of $Ru_3(CO)_{12}$ and 2-MeO-1-propanol (10 equiv.)	26	47	0
9	3.33 mol% of $Ru_3(CO)_{12}$ and 10 mol% I_2	96	0	0
10	4 mol% $PdCl_2$, 4 mol% dppf and 3.33 mol% of $Ru_3(CO)_{12}$	90	0	7
11	10 mol% $Ru(H_2)(CO)(PPh_3)_3$	79	21	0
12	3.33 mol% of $Ru_3(CO)_{12}$ and ethylene glycol (20 equiv.)	24	51	0
13	3.33 mol% of $Ru_3(CO)_{12}$ and gylcerol (20 equiv.)	0	22	0
14	3.33 mol% of $Ru_3(CO)_{12}$ and 0.5 equiv. KO*t*Bu	6	74	0
15	6.66 mol% of $Ru_3(CO)_{12}$ and 0.25 equiv. KO*t*Bu	12	60	0
16	6.66 mol% of $Ru_3(CO)_{12}$ and 0.5 equiv. KO*t*Bu	28	55	0
17	3.33 mol% of $Ru_3(CO)_{12}$ and without KO*t*Bu	6	68	0
18	3.33 mol% of $Ru_3(CO)_{12}$ and HCOOH instead of alcohol (44 equiv.)	50	36	0

Standard conditions: phenylacetylene (0.6 mmol, 1.2 equiv.), 4-iodotoluene (0.5 mmol, 1 equiv.), $PdCl_2(PPh_3)_2$ (4 mol%), CuI (5 mol%), triethylamine (3 mL), stirring at room temperature for 4 hours. Then added: Grubbs catalyst M102 (10 mol% Ru), KO*t*Bu (2 equiv.), benzyl alcohol (10 equiv.). Stirred at 100 °C for 24 hours.

2.4. Substrate scope of full hydrogenation

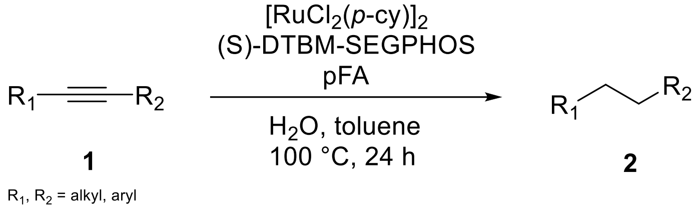

Optimized reaction protocol (Supporting Table S1, entry 39) was taken for substrate scope screening.

Into a screw-capped glass tube were added in the following order: internal alkyne (or olefin) (0.5 mmol, 1 equiv.), dichloro(*p*-cymene)ruthenium(II) ($[RuCl_2(p\text{-cy})]_2$, 7.7 mg, 0.0125 mmol, 2.5 mol%, total 5 mol% Ru), (S)-DTBM-SEGPHOS (29.5 mg, 0.025 mmol, 5 mol%), paraformaldehyde (pFA, 150 mg, 5 mmol, 10 equiv.), toluene (2 mL) and water (1 mL). Glass tube was tightly closed with a stirr cap and placed into an oil bath pre-heated to 100 °C and the reaction mixture was stirred for 24 hours. No precaution was taken to exclude air. Then, the reaction tube was taken out of oil bath and reaction mixture was cooled to room temperature to decrease internal pressure and opened.. The reaction mixture was filtered through a pad of SiO_2 using ethyl acetate as an eluent. Ethyl acetate was removed under reduced pressure and crude product was purified with SiO_2 column chromatography with Interchim puriFlash XS520 Plus using petroleum ether/ethyl acetate as eluent (unless noted otherwise). 40 g SiO_2 cartridges were used with elution program at 26 mL/min: 2 CV at 100% petroleum ether followed by 15 CV gradient to 50/50 petroleum ether/ethyl acetate.

1,2-Diphenylethane (2a)

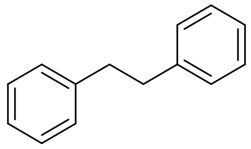

The product was isolated as a white crystalline solid: on a 0.5 mmol scale 90 mg (99 %); on a 2.0 mmol scale 287 mg (79 %).

With benzene/H_2O (1/1 mL) solvent mixture: Conversion to product: 100%.

Product was also prepared with 100% conversion from either (*E*)- or (*Z*)-stilbene.

^1H NMR ($CDCl_3$, 600 MHz) δ 7.31–7.26 (m, 4H), 7.22–7.18 (m, 6H), 2.93 (s, 4H). ^1H NMR data is in agreement with literature reports.[1]

1-Methyl-4-phenethylbenzene (2b)

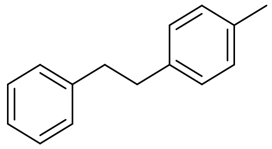

Product was isolated as a colourless oil, 90 mg (92%).

^1H NMR ($CDCl_3$, 600 MHz) δ 7.34–7.29 (m, 2H), 7.25–7.20 (m, 3H), 7.15–7.10 (m, 4H), 2.96–2.89 (m, 4H), 2.36 (s, 3H). ^1H NMR data is in agreement with literature reports.[2]

1-Methyl-4-(4-(trifluoromethyl)phenethyl)benzene (2c)

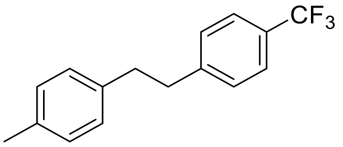

Product was isolated as a white crystalline solid, 114 mg (86%).

^{1}H NMR ($CDCl_3$, 600 MHz) δ 7.53 (d, *J* = 8.0 Hz, 2H), 7.28 (d, *J* = 8.0 Hz, 2H), 7.11 (d, *J* = 7.8 Hz, 2H), 7.06 (d, *J* = 7.8 Hz, 2H), 2.99–2.94 (m, 2H), 2.93–2.88 (m, 2H), 2.34 (s, 3H). ^{1}H NMR data is in agreement with literature reports.[3]

1-(4-Phenethylphenyl)ethan-1-ol (2d)

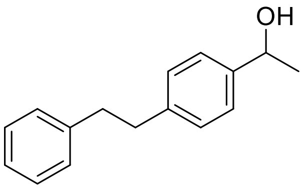

Product was prepared from corresponding internal alkyne with acetophenone functional group ((1-(4-(phenylethynyl)phenyl)ethan-1-one (**1d**)). Carbonyl group was reduced into alcohol during the reaction affording title compound **2d**. Product was isolated as a white solid, 93 mg, 82%.

^{1}H NMR ($CDCl_3$, 600 MHz) δ 7.32–7.28 (m, 4H), 7.23–7.17 (m, 5H), 4.89 (q, *J* = 6.5 Hz, 1H), 2.93 (s, 4H), 1.78 (br, 1H), 1.50 (d, *J* = 6.5 Hz, 3H).

^{13}C{^{1}H} NMR ($CDCl_3$, 150 MHz) δ 143.6, 141.9, 141.3, 128.7, 128.6, 128.5, 126.1, 125.6, 70.4, 38.1, 37.7, 25.3.

HRMS–ESI (*m/z*): $[M + H]^+$ calcd for $C_{16}H_{17}O^+$, 209.1325; found, 209.1327, which corresponds to [M-$H_2O]^+$.

CHIRAL HPLC ANALYSIS:

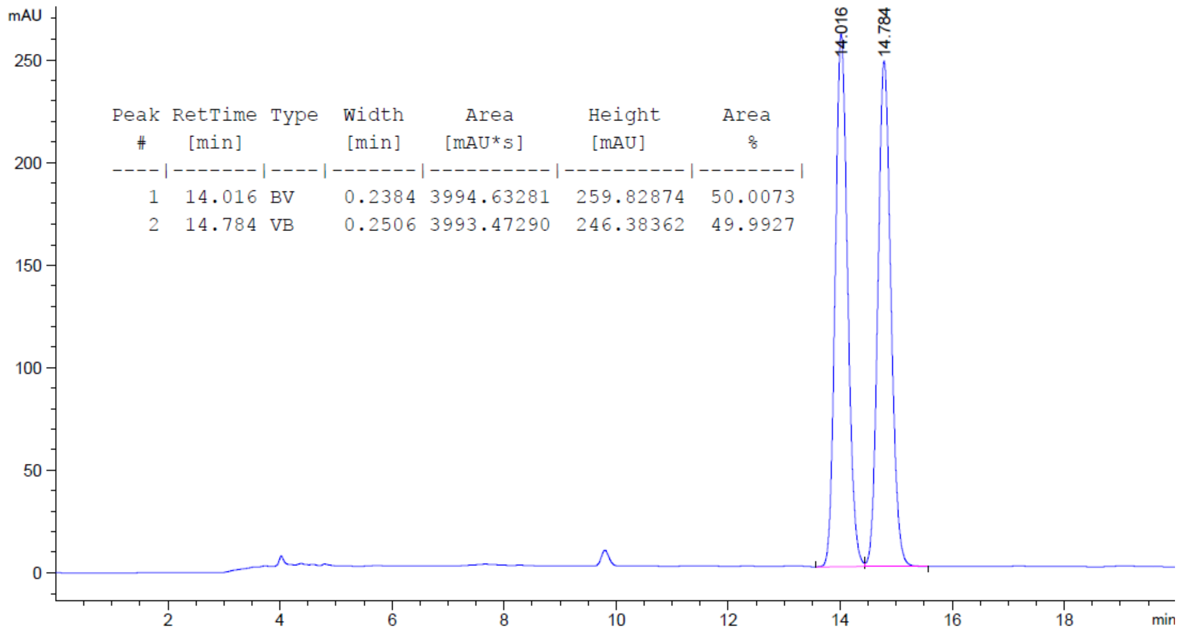

Chiral HPLC analysis of 1-(4-Phenethylphenyl)ethan-1-ol (**2d**) using Chiralcel AD-H.

4-(4-Methoxyphenethyl)phenol (2e)

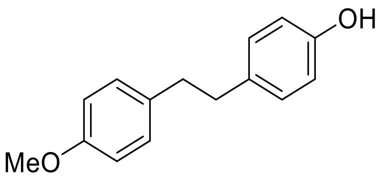

Product was isolated as a white crystalline solid, 96 mg, 84%.

^{1}H NMR (CDCl$_3$, 600 MHz) δ 7.07 (d, *J* = 8.5 Hz, 2H), 7.02 (d, *J* = 8.5 Hz, 2H), 6.82 (d, *J* = 8.5 Hz, 2H), 6.74 (d, *J* = 8.5 Hz, 2H), 4.62 (br, 1H), 3.79 (s, 3H), 2.81 (s, 4H).

^{13}C{^{1}H} NMR (CDCl$_3$, 150 MHz) δ 157.9, 154.8, 134.3, 134.1, 129.8, 129.6, 115.3, 113.9, 55.5, 37.46, 37.44.

HRMS–ESI (*m/z*): [M + H]$^+$ calcd for $C_{15}H_{17}O_2^+$, 229.1223; found, 229.1228.

1-(2-Cyclohexylethyl)-4-methoxybenzene (2f)

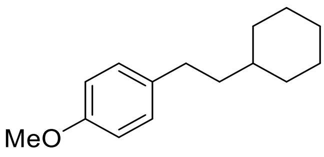

Product was isolated as a colourless oil, 67 mg, 61%.

^{1}H NMR ($CDCl_3$, 600 MHz) δ 7.11–7.08 (m, 2H), 6.85–6.80 (m, 2H), 3.79 (s, 3H), 2.58–2.53 (m, 2H), 1.79–1.73 (m, 2H), 1.73–1.67 (m, 2H), 1.67–1.62 (m, 1H), 1.50–1.44 (m, 2H), 1.31–1.10 (m, 4H), 0.97–0.88 (m, 2H). ^{1}H NMR data is in agreement with literature reports.[4]

5-(4-Fluorophenethyl)benzo[d][1,3]dioxole (2g)

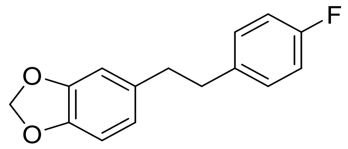

Product isolated as a white crystalline solid, 79 mg, 65%.

^{1}H NMR ($CDCl_3$, 600 MHz) δ 7.12–7.07 (m, 2H), 6.98–6.93 (m, 2H), 6.71 (d, J = 7.9 Hz, 1H), 6.65 (d, J = 1.7 Hz, 1H), 6.60–6.56 (m, 1H), 5.92 (s, 2H), 2.87–2.78 (m, 4H). ^{1}H NMR data is in agreement with literature reports.[5]

2-Chloro-1-(4-chlorophenethyl)-4-nitrobenzene (2h)

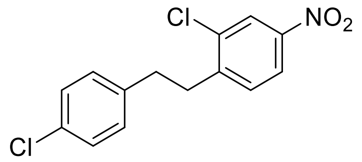

Product isolated as a white crystalline solid, 132 mg, 89%.

^{1}H NMR ($CDCl_3$, 600 MHz) δ 8.25 (d, J = 2.3 Hz, 1H), 8.01 (dd, J = 8.4, 2.3 Hz, 1H), 7.27–7.24 (m, 3H), 7.09–7.07 (m, 2H), 3.10 (dd, J = 9.0, 6.7 Hz, 2H), 2.92 (dd, J = 9.0, 6.7 Hz, 2H).

$^{13}C\{^1H\}$ NMR ($CDCl_3$, 150 MHz) δ 147.1, 146.5, 138.8, 135.0, 132.4, 131.2, 130.0, 128.9, 125.0, 121.9, 35.9, 34.8.

HRMS–ESI (*m/z*): $[M + H]^+$ calcd for $C_{14}H_{12}Cl_2NO_2^+$, 296.0240; found, 296.0240.

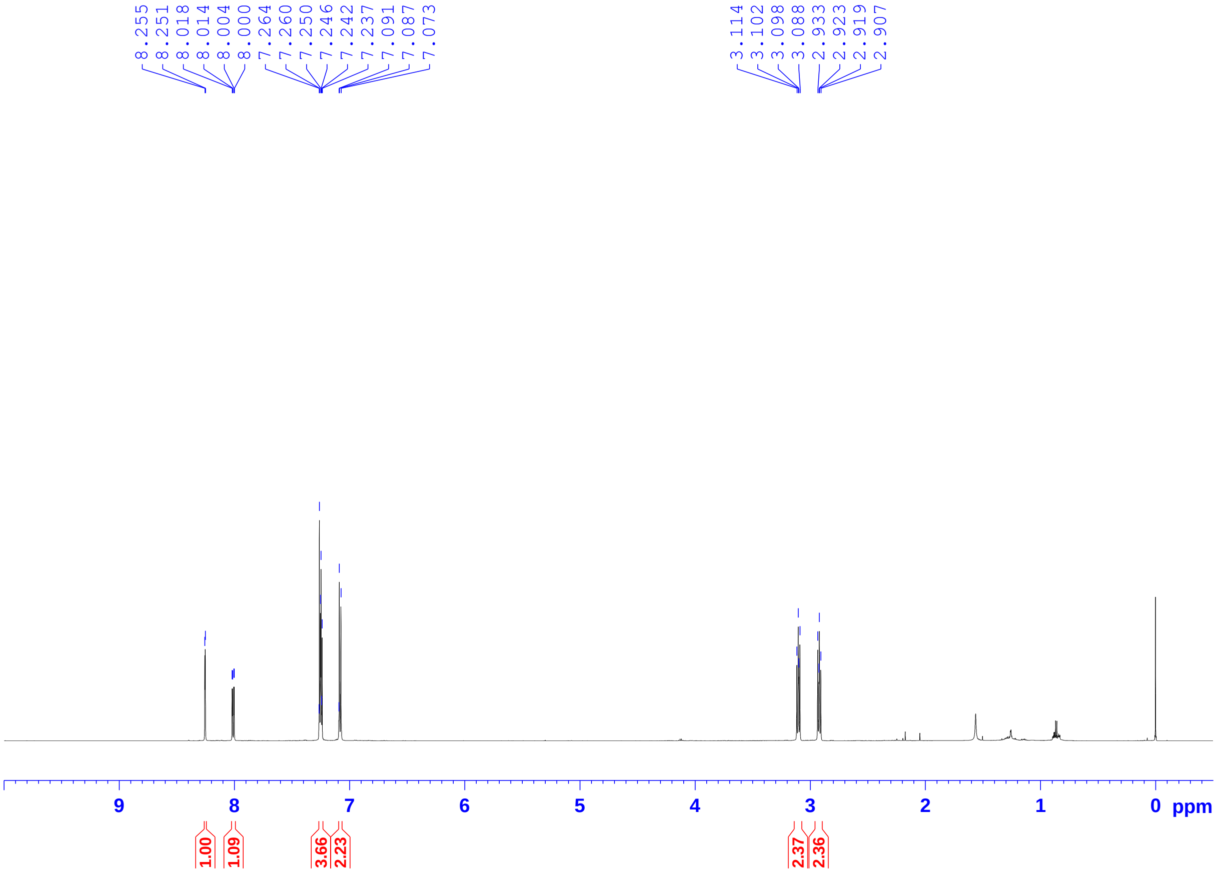

Ethyl 3-phenylpropanoate (2i)

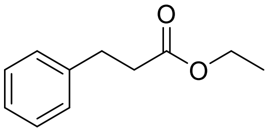

Product isolated as a yellow oil, 79 mg, 89%.

^{1}H NMR ($CDCl_3$, 600 MHz) δ 7.31–7.27 (m, 2H), 7.23–7.19 (m, 3H), 4.13 (q, *J* = 7.1 Hz, 2H), 2.96 (t, *J* = 7.9 Hz, 2H), 2.62 (t, *J* = 7.9 Hz, 2H), 1.24 (t, *J* = 7.1 Hz, 3H). ^{1}H NMR data is in agreement with literature reports.[6]

1-(6-Chlorohexyl)-4-nitrobenzene (2j)

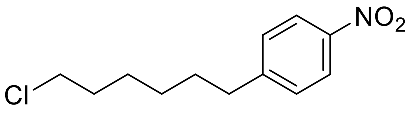

Product isolated as a yellow oil, 110 mg, 91%.

^{1}H NMR ($CDCl_3$, 600 MHz) δ 8.17–8.11 (m, 2H), 7.35–7.30 (m, 2H), 3.53 (t, *J* = 6.6 Hz, 2H), 2.75–2.69 (m, 2H), 1.81–1.73 (m, 2H), 1.70–1.64 (m, 2H), 1.51–1.45 (m, 2H), 1.40–1.33 (m, 2H).

^{13}C{^{1}H} NMR ($CDCl_3$, 150 MHz) δ 150.6, 146.5, 129.3, 123.8, 45.2, 35.9, 32.6, 31.0, 28.6, 26.8.

HRMS–ESI (*m/z*): $[M + H]^+$ calcd for $C_{12}H_{17}ClNO_2^+$, 242.0942; found, 242.0943.

(4-(2-(Naphthalen-1-yl)ethyl)phenyl)methanol (2k)

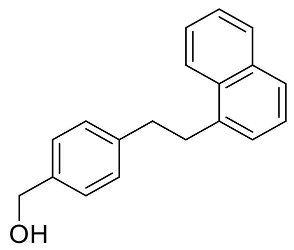

Product was prepared from corresponding internal alkyne with aldehyde functional group (4-(naphthalen-1-ylethynyl)benzaldehyde (**1k**)). Aldehyde moiety was reduced during the reaction into hydroxy group affording title compound 2k. White crystalline solid, 60 mg, 46%.

With benzene/H_2O (1/1 mL) solvent mixture: Conversion to product: 62%.

^{1}H NMR ($CDCl_3$, 600 MHz) δ 8.09 (d, *J* = 8.4 Hz, 1H), 7.87 (dd, *J* = 8.1, 1.5 Hz, 1H), 7.72 (d, *J* = 8.2 Hz, 1H), 7.53 (ddd, *J* = 8.4, 6.8, 1.5 Hz, 1H), 7.48 (ddd, *J* = 8.0, 6.7, 1.3 Hz, 1H), 7.39–7.36 (m, 1H), 7.32–7.27 (m, 3H), 7.25–7.22 (m, 2H), 4.66 (s, 2H), 3.38–3.34 (m, 2H), 3.07–3.03 (m, 2H). The hydroxyl proton resonance is missing.

^{13}C{^{1}H} NMR ($CDCl_3$, 150 MHz) δ 141.7, 138.8, 137.8, 134.1, 131.9, 129.0, 128.8, 127.4, 127.0, 126.2, 126.1, 125.71, 125.65, 123.8, 65.4, 36.9, 35.3.

HRMS–ESI (*m/z*): $[M + H]^+$ calcd for $C_{19}H_{17}O^+$, 245.1325; found, 245.1324 which corresponds to $[M-H_2O]^+$.

Di-*tert*-butyl succinate (2l)

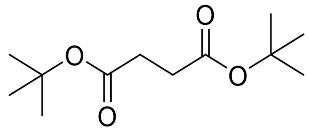

Product isolated as a colourless oil, 49 mg, 43%.

With benzene/H_2O (1/1 mL) solvent mixture: Conversion to product: 55%.

^1H NMR ($CDCl_3$, 600 MHz) δ 2.48 (s, 4H), 1.44 (s, 18H). ^1H NMR data is in agreement with literature reports.[7]

2-Chloro-1-(2-(4-chlorophenyl)ethyl-1,1,2,2-d_4)-4-nitrobenzene (2m)

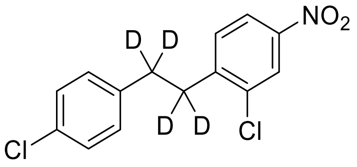

Deuterated paraformaldehyde (pFA-d_2) and deuterated water (D_2O) were used. Product was isolated as a yellow crystalline solid, 121 mg, 81%.

^1H NMR ($CDCl_3$, 600 MHz) δ 8.25 (d, J = 2.2 Hz, 1H), 8.01 (dd, J = 8.4, 2.2 Hz, 1H), 7.26–7.23 (m, 2H), 7.09–7.06 (m, 2H).

$^{13}C\{^1H\}$ NMR ($CDCl_3$, 150 MHz) δ 147.1, 146.4, 138.8, 135.0, 132.4, 131.2, 130.0, 128.9, 124.9, 121.9. Aliphatic carbons are not visible in spectrum.

HRMS–ESI (*m/z*): $[M + H]^+$ calcd for $C_{14}H_8D_4Cl_2NO_2^+$, 300.0491; found, 300.0491.

4-(4-hydroxy-3-methoxyphenethyl)-2,6-dimethoxyphenol (moscatilin) (2n)

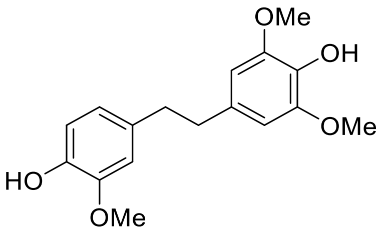

Benzene/H_2O (1/1 mL) solvent mixture was used. Reaction time was 48 hours. After 48 hours, reaction mixture was transferred to a separatory funnel. Water (30 mL) was added and acidified to pH 2 with 2M HCl. Product was extracted with ethyl acetate (3 x 30 mL). Ethyl acetate was dried over anhydrous sodium sulphate and removed under reduced pressure. Crude product was loaded onto Celite and purified with SiO_2 column chromatography using automated purification system Interchim puriFlash XS520 with petroleum ether/ethyl acetate as eluent. Product was isolated as a yellow oil, 79 mg, 52%.

^1H NMR ($CDCl_3$, 600 MHz) δ 7.42–7.38 (m, 2H), 7.02 (d, J = 16.2 Hz, 1H), 6.90 (d, J = 16.2 Hz, 1H), 6.85–6.81 (m, 2H), 6.65 (d, J = 2.2 Hz, 2H), 6.39–6.37 (m, 1H), 3.83 (s, 6H). ^1H NMR data is in agreement with the literature reports.[8]

Methyl 3-phenylpropanoate (2o)

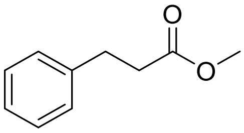

Product **2o** was prepared from (*E*)-olefin starting material, i.e. methyl cinnamate. Isolated as a colourless oil, 79 mg, 96%.

^{1}H NMR ($CDCl_3$, 600 MHz) δ 7.31–7.27 (m, 2H), 7.23–7.19 (m, 3H), 3.68 (s, 3H), 2.96 (t, *J* = 7.9 Hz, 2H), 2.64 (t, *J* = 7.9 Hz, 2H). ^{1}H NMR data is in agreement with the literature reports.[9]

Pyridine substrate limitation observed

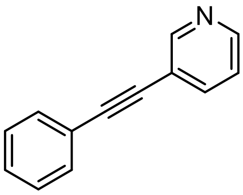

<30 % conversion to product

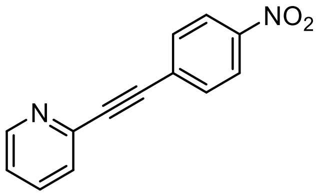

<30 % conversion to product

In the case of pyridine substrates the conversion to fully hydrogenated product was under 30%. The product were not isolated due to poor conversions.

2.5. Substrate scope of one-pot sequential Pd/Cu Sonogashira cross-coupling and Ru-catalyzed hydrogenation to alkenes

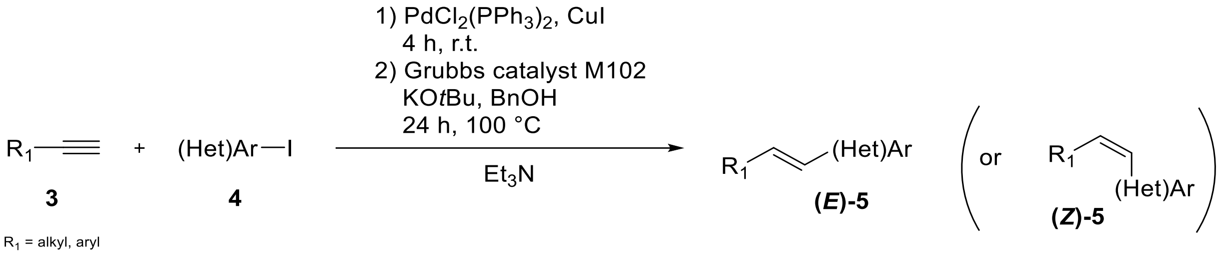

Oven-dried 10 mL round bottom reaction flask was equipped with a rubber septum and purged with nitrogen. Under nitrogen atmosphere were added in the following order: triethylamine (3 mL), bis(triphenylphosphine)palladium(II) dichloride ($PdCl_2(PPh_3)_2$, 14 mg, 0.02 mmol, 4 mol%), copper(I) iodide (CuI, 4.8 mg, 0.025 mmol, 5 mol%), aryl iodide (0.5 mmol, 1 equiv.) and terminalalkyne (0.6 mmol, 1.2 equiv.). Reaction fFlask was equipped with nitrogen balloon and the reaction mixture was stirred at room temperature for 4 hours. After 4 hours, the following reagents were added in the flask under the flow of nitrogen: Grubbs catalyst M102 (benzylidene-bis(tricyclohexylphosphine)dichlororuthenium, 41 mg, 0.05 mmol, 10 mol%), potassium *tert*-butoxide (KO*t*Bu, 112 mg, 1 mmol, 2 equiv.) and benzyl alcohol (BnOH, 521 mg, 499 µL, 5 mmol, 10 equiv.). Reaction flask was capped with a glass stopper and placed into an oil bath pre-heated to 100 °C and the reaction mixture was stirred for 24 hours. After 24 hours, the reaction mixture was filtered through a pad of SiO_2 using ethyl acetate as an eluent. Ethyl acetate was removed under reduced pressure and crude product was purified with SiO_2 column chromatography with Interchim puriFlash XS520 Plus using petroleum ether/ethyl acetate as eluent. 40 g SiO_2 cartridges were used with elution program at 26 mL/min: 2 CV at 100% petroleum ether followed by 15 CV gradient to 50/50 petroleum ether/ethyl acetate.

(*E*)-1-Methyl-4-styrylbenzene (5a)

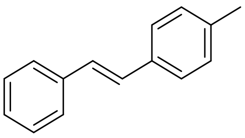

On a 0.5 mmol scale: 4-Iodotoluene (109 mg, 0.5 mmol, 1 equiv.), phenylacetylene (66 µL, 61 mg, 0.6 mmol, 1.2 equiv.). Product was isolated in form of white crystals, 96 mg (99%).

On a 2.0 mmol scale: 4-Iodotoluene (437 mg, 2.0 mmol, 1 equiv.), phenylacetylene (263 µL, 245 mg, 2.4 mmol, 1.2 equiv.). Product was isolated in form of white crystals, 349 mg (90%).

^{1}H NMR ($CDCl_3$, 600 MHz) δ 7.53–7.50 (m, 2H), 7.44–7.41 (m, 2H), 7.36 (t, J = 7.7 Hz, 2H), 7.27–7.24 (m, 1H), 7.18 (d, J = 7.7 Hz, 2H), 7.13–7.04 (m, 2H), 2.37 (s, 3H). ^{1}H NMR data is in agreement with reports from literature.[10]

(*E*)-4-(3,5-Dimethoxystyryl)phenol (pterostilbene) (5b)

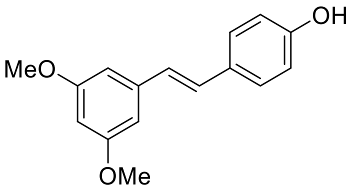

4-Iodophenol (110 mg, 0.5 mmol, 1 equiv.), 1-ethynyl-3,5-dimethoxybenzene (97 mg, 0.6 mmol, 1.2 equiv.). Product was isolated as a colourless oil, 82 mg, 64%.

^{1}H NMR ($CDCl_3$, 600 MHz) δ 7.42–7.38 (m, 2H), 7.02 (d, J = 16.2 Hz, 1H), 6.90 (d, J = 16.2 Hz, 1H), 6.84–6.81 (m, 2H), 6.65 (d, J = 2.2 Hz, 2H), 6.38 (t, J = 2.2 Hz, 1H), 3.83 (s, 6H). ^{1}H NMR data is in agreement with reports from literature.[11]

(*E*)-4-(4-Methoxystyryl)phenol (5c)

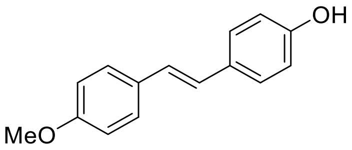

4-Iodophenol (110 mg, 0.5 mmol, 1 equiv.), 4-methoxyphenylacetylene (79 mg, 0.6 mmol, 1.2 equiv.). Product was isolated as an off-white solid, 89 mg, 79%.

^{1}H NMR ($CDCl_3$, 600 MHz) δ 7.44–7.41 (m, 2H), 7.40–7.37 (m, 2H), 6.92 (s, 2H), 6.90–6.87 (m, 2H), 6.83–6.80 (m, 2H), 3.83 (s, 3H). ^{1}H NMR data is in agreement with reports from literature.[12]

(*E*)-5-(4-Fluorostyryl)benzo[d][1,3]dioxole (5d)

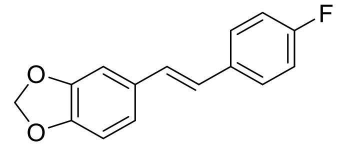

1-Fluoro-4-iodobenzene (111 mg, 58 µL, 0.5 mmol, 1 equiv.), 5-Ethynyl-1,3-benzodioxole (88 mg, 71 µL, 0.6 mmol, 1.2 equiv.). Product was isolated as a white crystalline solid, 81 mg, 67%.

^{1}H NMR ($CDCl_3$, 600 MHz) δ 7.46–7.41 (m, 2H), 7.06–7.01 (m, 3H), 6.96–6.87 (m, 3H), 6.80 (d, *J* = 8.0 Hz, 1H), 5.98 (s, 2H).

^{13}C{^{1}H} NMR ($CDCl_3$, 150 MHz) δ 163.2, 161.5, 148.4, 147.5, 133.8, 131.9, 128.3, 127.9, 126.0, 121.6, 115.9, 115.7, 108.6, 105.7, 101.3.

HRMS–ESI (*m/z*): $[M + H]^+$ calcd for $C_{15}H_{12}FO_2^+$, 243.0816; found, 243.0817.

(*E*)-4-(4-Hydroxy-3-methoxystyryl)-2,6-dimethoxyphenol (5e)

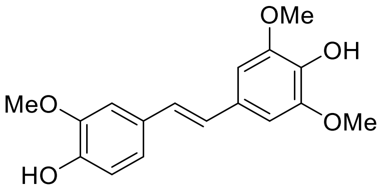

4-Iodo-2,6-dimethoxyphenol (**4a**) (140 mg, 0.5 mmol, 1 equiv.), 4-ethynyl-2-methoxyphenol (**3a**) (89 mg, 0.6 mmol, 1.2 equiv.). Product was isolated as a red crystalline solid, 59 mg, 39%.

^{1}H NMR ($CDCl_3$, 600 MHz) δ 7.03–6.99 (m, 2H), 6.91–6.87 (m, 3H), 6.73 (s, 2H), 5.64 (s, 1H), 5.55 (s, 1H), 3.95 (s, 3H), 3.94 (s, 6H).

^{13}C{^{1}H} NMR ($CDCl_3$, 150 MHz) δ 137.4, 146.9, 145.6, 134.7, 130.3, 129.4, 127.0, 126.8, 120.4, 114.8, 108.2, 103.2, 56.5, 56.1.

HRMS–ESI (*m/z*): $[M + H]^+$ calcd for $C_{17}H_{19}O_5^+$, 303.1227; found, 303.1229.

(*E*)-2-(3,5-Dimethoxystyryl)thiophene (5f)

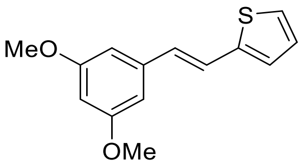

2-Iodotiophene (105 mg, 55 µL, 0.5 mmol, 1 equiv.), 1-ethynyl-3,5-dimethoxybenzene (97 mg, 0.6 mmol, 1.2 equiv.). Product was isolated as a colourless oil, 75 mg, 61%.

^{1}H NMR ($CDCl_3$, 600 MHz) δ 7.23–7.19 (m, 2H), 7.08 (d, *J* = 3.7 Hz, 1H), 7.02–7.00 (m, 1H), 6.86 (d, *J* = 16.1 Hz, 1H), 6.63 (d, *J* = 2.2 Hz, 2H), 6.39 (t, *J* = 2.2 Hz, 1H), 3.83 (s, 6H). ^{1}H NMR data is in agreement with reports from literature.[13]

(*E*)-1-(4-Chlorostyryl)-3-methoxybenzene (5g)

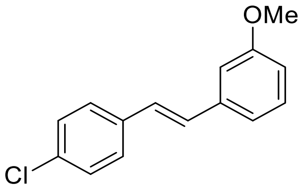

3-Iodoanisole (117 mg, 60 µL, 0.5 mmol, 1 equiv.), 4-ethynylchlorobenzene (82 mg, 0.6 mmol, 1.2 equiv.). Product was isolated as a yellow oil, 99 mg, 81%.

^{1}H NMR ($CDCl_3$, 600 MHz) δ 7.46–7.42 (m, 2H), 7.34–7.31 (m, 2H), 7.31–7.26 (m, 1H), 7.12–7.09 (m, 1H), 7.05 (s, 2H), 7.05–7.03 (m, 1H), 6.86–6.82 (m, 1H), 3.85 (s, 3H). ^{1}H NMR data is in agreement with reports from literature.[14]

(*E*)-1-(Hex-1-en-1-yl)-4-(trifluoromethyl)benzene (5h)

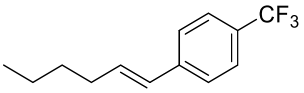

4-Iodobenzotrifluoride (136 mg, 74 µL, 0.5 mmol, 1 equiv.), 1-hexyne (49 mg, 69 µL, 0.6 mmol, 1.2 equiv.). Product was isolated as a colourless oil, 91 mg, 79%.

^{1}H NMR ($CDCl_3$, 600 MHz) δ 7.53 (d, J = 8.0 Hz, 2H), 7.42 (d, J = 8.0 Hz, 2H), 6.40 (d, J = 15.9 Hz, 1H), 6.37–6.30 (m, 1H), 2.24 (q, J = 7.2 Hz, 2H), 1.50–1.44 (m, 2H), 1.42–1.34 (m, 2H), 0.93 (t, J = 7.3 Hz, 3H). ^{1}H NMR data is in agreement with reports from literature.[15]

(*E*)-4-(3,5-Dimethoxystyryl)-2-methoxyphenol (isorhapontigenin) (5i)

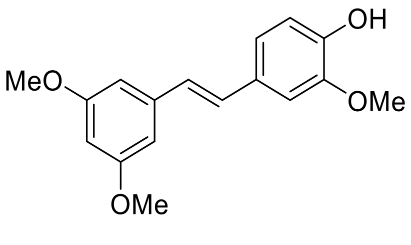

4-Iodo-2-methoxyphenol (125 mg, 0.5 mmol, 1 equiv.), 3,5-dimethoxyphenylacetylene (97 mg, 0.6 mmol, 1.2 equiv.). Product was isolated as a yellow solid/paste, 86 mg, 60%.

^{1}H NMR ($CDCl_3$, 500 MHz) δ 7.05–7.00 (m, 3H), 6.92–6.86 (m, 2H), 6.65 (d, J = 2.2 Hz, 2H), 6.38 (t, J = 2.2 Hz, 1H), 5.67 (s, 1H), 3.96 (s, 3H), 3.83 (s, 6H). ^{1}H NMR data is in agreement with reports from literature.[16]

(*E*)-1,2-Diphenylethene (5j)

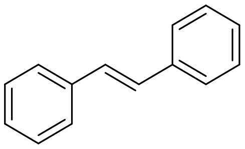

4-Iodobromobenzene (142 mg, 0.5 mmol, 1 equiv.), phenylacetylene (61 mg, 66 µL, 0.6 mmol, 1.2 equiv.). Under these reaction conditions dehalogenation of aryl bromide occurs. Product was isolated as a pale yellow crystalline solid, 70 mg, 78%.

^{1}H NMR ($CDCl_3$, 600 MHz) δ 7.54–7.51 (m, 4H), 7.38–7.34 (m, 4H), 7.28–7.25 (m, 2H), 7.12 (s, 2H). ^{1}H NMR data is in agreement with reports from literature.[17]

(*E*)-3-(2-(Thiophen-3-yl)vinyl)phenol (5k)

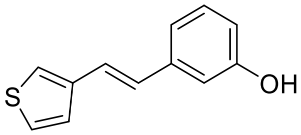

3-Iodophenol (110 mg, 0.5 mmol, 1 equiv.), 3-ethynyltiophene (65 mg, 59 µL, 0.6 mmol, 1.2 equiv.). Product was isolated as a white solid, 38 mg, 38%.

^{1}H NMR ($CDCl_3$, 600 MHz) δ 7.35–7.31 (m, 2H), 7.27–7.26 (m, 1H), 7.22 (t, *J* = 7.9 Hz, 1H), 7.10 (d, *J* = 16.3 Hz, 1H), 7.06–7.04 (m, 1H), 6.97–6.95 (m, 1H), 6.89 (d, *J* = 16.3 Hz, 1H), 6.74–6.71 (m, 1H), 4.82 (s, 1H).

^{13}C{^{1}H} NMR ($CDCl_3$, 150 MHz) δ 155.9, 140.1, 139.3, 130.0, 128.4, 126.4, 125.1, 123.6, 122.8, 119.4, 114.7, 112.9.

HRMS–ESI (*m/z*): $[M + H]^+$ calcd for $C_{12}H_{11}OS^+$, 203.0525; found, 203.0526.

(*E*)-1-(Tetradec-1-en-1-yl)-3,5-bis(trifluoromethyl)benzene (5l)

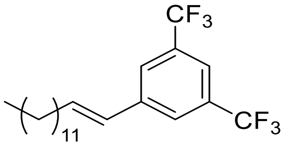

1-Iodo-3,5-bis(trifluoromethyl)benzene (170 mg, 89 µL, 0.5 mmol, 1 equiv.), 1-tetradecyne (117 mg, 148 µL**,** 0.6 mmol, 1.2 equiv). Product isolated as yellow oil, 180 mg, 88%.

^{1}H NMR ($CDCl_3$, 600 MHz) δ 7.74 (s, 2H), 7.67 (s, 1H), 6.46–6.37 (m, 2H), 2.28–2.23 (m, 2H), 1.52–1.45 (m, 2H), 1.33–1.21 (m, 18H), 0.88 (m, 3H).

^{13}C{^{1}H} NMR ($CDCl_3$, 150 MHz) δ 140.1, 135.9, 131.9 (q), 127.4, 125.9, 124.5, 122.7, 120.3 (q), 33.2, 32.1, 29.87, 29.85, 29.84, 29.78, 29.68, 29.55, 29.4, 29.2, 22.9, 14.3.

HRMS–ESI (*m/z*): Does not ionize under ESI+ conditions.

(*E*)-4-(4-(Dimethylamino)styryl)phenol (5m)

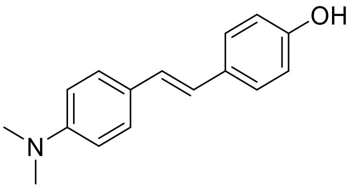

4-Iodophenol (110 mg, 0.5 mmol, 1 equiv.), 4-ethynyl-N,N-dimethylaniline (87 mg, 0.6 mmol, 1.2 equiv.). Product was isolated as a brown solid, 82 mg, 69%.

^{1}H NMR (DMSO-d_6, 600 MHz) δ 9.44 (s, 1H), 7.38–7.31 (m, 4H), 6.91–6.83 (m, 2H), 6.75–6.72 (m, 2H), 6.71–6.68 (m, 2H), 2.91 (s, 6H).

^{13}C{^{1}H} NMR (DMSO-d_6, 150 MHz) δ 156.5, 149.6, 128.9, 127.1, 127.0, 125.6, 125.4, 123.8, 115.5, 112.3, 40.0.

HRMS–ESI (*m/z*): $[M + H]^+$ calcd for $C_{16}H_{18}NO^+$, 240.1383; found, 240.1382.

(*E*)-1-(4-Fluorophenoxy)-3-(pent-1-en-1-yl)benzene (5n)

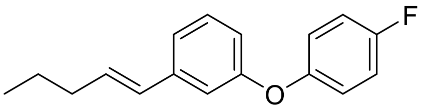

4-Fluoro-3'-iodophenyl ether (157 mg, 0.5 mmol, 1 equiv.), pentyne (41 mg, 60 µL, 0.6 mmol, 1.equiv.). Product was isolated as a yellow oil, 67 mg, 52%.

^{1}H NMR ($CDCl_3$, 600 MHz) δ 7.24 (t, *J* = 7.9 Hz, 1H), 7.09–7.06 (m, 1H), 7.05–7.00 (m, 2H), 7.00–6.94 (m, 3H), 6.81–6.77 (m, 1H), 6.33 (d, *J* = 15.8 Hz, 1H), 6.23–6.17 (m, 1H), 2.20–2.14 (qd, *J* = 7.1, 1.4 Hz, 2H), 1.52–1.44 (m, 2H), 0.94 (t, *J* = 7.4 Hz, 3H).

^{13}C{^{1}H} NMR ($CDCl_3$, 150 MHz) δ 158.0, 140.1, 132.2, 129.9, 129.4, 121.2, 120.62, 120.56, 116.9, 116.5, 116.4, 115.9, 35.2, 22.6, 13.9.

HRMS–ESI (*m/z*): $[M + H]^+$ calcd for $C_{17}H_{18}FO^+$, 257.1336; found, 257.1336.

(*E*)-1-(4-styrylphenyl)ethan-1-ol (5o)

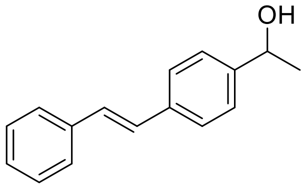

1-(4-Iodophenyl)ethan-1-ol (**4c**) (124 mg, 0.5 mmol, 1 equiv.), phenylacetylene (66 µL, 61 mg, 0.6 mmol, 1.2 equiv.). Product was isolated as a white solid, 71 mg, 63%.

^{1}H NMR ($CDCl_3$, 600 MHz) δ 7.53–7.49 (m, 4H), 7.39–7.34 (m, 4H), 7.28–7.24 (m, 1H), 7.11 (s, 2H), 4.92 (q, *J* = 6.5 Hz, 1H), 1.79 (br, 1H), 1.52 (d, *J* = 6.5 Hz, 3H). ^{1}H NMR data is in agreement with reports from literature.[18]

(*E*)-2-Methoxy-5-(3,4,5-trimethoxystyryl)phenol ("*trans*-combretastatin") (5p)

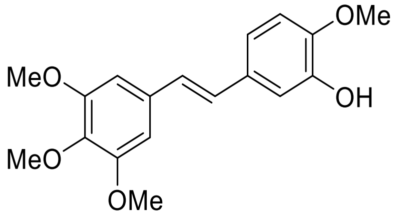

5-Iodo-2-methoxyphenol (**4b**) (125 mg, 0.5 mmol, 1 equiv.), 5-ethynyl-1,2,3-trimethoxybenzene (**3b**) (115 mg, 0.6 mmol, 1.2 equiv.). Product was isolated as an orange solid, 102 mg, 65%.

^{1}H NMR ($CDCl_3$, 600 MHz) δ 7.14 (d, *J* = 2.1 Hz, 1H), 6.97 (dd, *J* =8.3, 2.1 Hz, 1H), 6.94–6.87 (m, 2H), 6.71 (s, 2H), 5.63 (s, 1H), 3.92–3.91 (m, 9H), 3.86 (s, 3H). ^{1}H NMR data is in agreement with reports from literature.[19]

(Z)-1-Methyl-4-styrylbenzene (5r)

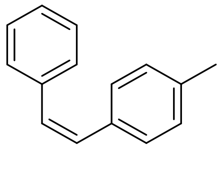

Reaction conditions from Supporting Table 3, entry 14.

4-Iodotoluene (109 mg, 0.5 mmol, 1 equiv.), phenylacetylene (61 mg, 66 µL, 0.6 mmol, 1.2 equiv.). Triruthenium dodecacarbonyl (10.9 mg, 0.017 mmol, 3.33 mol% (10 mol% Ru)) used with reduced amount of KO*t*Bu (28 mg, 0.25 mmol, 0.5 equiv.). Product was isolated as a colourless oil, 68 mg, 70%.

^{1}H NMR ($CDCl_3$, 600 MHz) δ 7.28–7.24 (m, 2H), 7.24–7.21 (m, 2H), 7.20–7.16 (m, 1H), 7.15–7.13 (m, 2H), 7.04–7.00 (m, 2H), 6.55 (s, 2H), 2.31 (s, 3H). ^{1}H NMR data is in agreement with reports from literature.[20]

(*Z*)-2-Methoxy-5-(3,4,5-trimethoxystyryl)phenol (combretastatin A4) (5s)

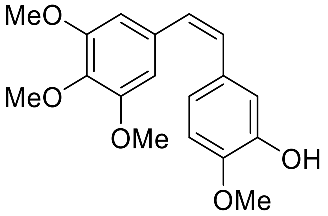

Reaction conditions from Supporting Table 3, entry 14.

5-Iodo-2-methoxyphenol (**4b**) (125 mg, 0.5 mmol, 1 equiv.), 5-ethynyl-1,2,3-trimethoxybenzene (**3b**) (115 mg, 0.6 mmol, 1.2 equiv.). Triruthenium dodecacarbonyl (10.9 mg, 0.017 mmol, 3.33 mol% (10 mol% Ru)) used with reduced amount of KO*t*Bu (28 mg, 0.25 mmol, 0.5 equiv.). Product was isolated as a slightly yellow solid, 90 mg, 57%.

^{1}H NMR ($CDCl_3$, 600 MHz) δ 6.92 (d, *J* = 2.0 Hz, 1H), 6.90 (dd, *J* = 8.3, 2.0 Hz, 1H), 6.73 (d, *J* = 8.3 Hz, 1H), 6.52 (s, 2H), 6.48–6.40 (m, 2H), 5.52 (s, 1H), 3.87 (s, 3H), 3.84 (s, 3H), 3.70 (s, 6H). ^{1}H NMR data is in agreement with reports from literature.[19]

Ketone and nitro group limitations observed

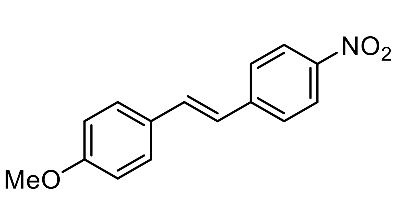

<30% conversion

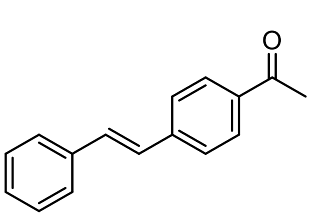

<30% conversion

In the case of 4-nitroiodobenzene and 4-iodoacetophenone the conversion to (E)-alkene was under 30%. Reaction mixture was complex due to unknown side reactions. The products were not isolated.

2.6. Synthesis of internal alkynes

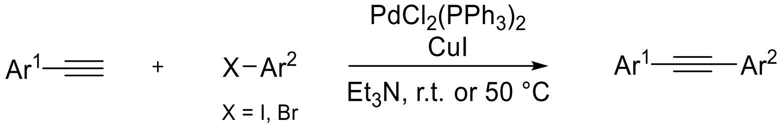

The synthesis of starting internal alkynes was done following modified protocol from the literature.[21]

Oven-dried 25 mL round bottom flask was equipped with a septum and purged with nitrogen. Under nitrogen flow were added in the following order: triethylamine (5 mL), $PdCl_2(PPh_3)_2$ (70 mg, 0.1 mmol, 5 mol%), CuI (19 mg, 0.1 mmol, 5 mol%), aryl halide (2 mmol, 1 equiv.) and terminal alkyne (2.4 mmol, 1.2 equiv.). Reaction was stirred at room temperature for reactions with aryl iodides and at 50 °C for reactions with aryl bromides and monitored with TLC. Upon consumption of starting aryl halide, triethylamine was removed under reduced pressure and crude product was loaded onto Celite (unless otherwise noted). Product was purified with SiO_2 column chromatography using automated purification system Interchim puriFlash XS520 with petroleum ether/ethyl acetate as eluent. 40 g SiO_2 cartridges were used with elution program at 26 mL/min: 2 CV at 100% petroleum ether followed by 15 CV gradient to 50/50 petroleum ether/ethyl acetate.

1-Methyl-4-(phenylethynyl)benzene (1b)

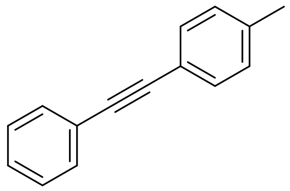

4-Iodotoluene (437 mg, 2 mmol, 1 equiv.), phenylacetylene (264 µL, 245 mg, 2.4 mmol, 1.2 equiv.). Product was isolated as a white crystalline solid, 362 mg, 94%.

^{1}H NMR ($CDCl_3$, 600 MHz) δ 7.55–7.51 (m, 2H), 7.45–7.41 (m, 2H), 7.37–7.29 (m, 3H), 7.18–7.14 (m, 2H), 2.37 (s, 3H). ^{1}H NMR data is in agreement with the literature.[22]

1-Methyl-4-((4-(trifluoromethyl)phenyl)ethynyl)benzene (1c)

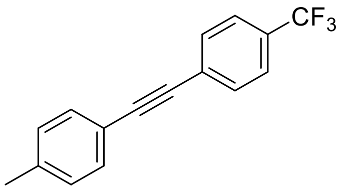

4-Iodobenzotrifluoride (544 mg, 296 µL, 2 mmol, 1 equiv.), 4-methylphenylacetylene (279 mg, 2.4 mmol, 1.2 equiv.). Product was isolated as a white crystalline solid, 502 mg, 96%.

^{1}H NMR ($CDCl_3$, 600 MHz) δ 7.64–7.58 (m, 4H), 7.45 (d, J = 8.2 Hz, 2H), 7.18 (d, J = 8.2 Hz, 2H), 2.38 (s, 3H). ^{1}H NMR data is in agreement with the literature.[23]

1-(4-(Phenylethynyl)phenyl)ethan-1-one (1d)

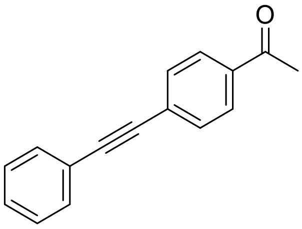

4-Iodoacetophenone (492 mg, 2 mmol, 1 equiv.), phenylacetylene (264 µL, 245 mg, 2.4 mmol, 1.2 equiv.). Product isolated as an off-white crystalline solid, 428 mg, 97%.

^{1}H NMR ($CDCl_3$, 600 MHz) δ 7.94 (d, J = 8.3 Hz, 2H), 7.61 (d, J = 8.3 Hz, 2H), 7.58–7.53 (m, 2H), 7.39–7.36 (m, 3H), 2.62 (s, 3H). ^{1}H NMR data is in agreement with the literature.[22]

4-((4-Methoxyphenyl)ethynyl)phenol (1e)

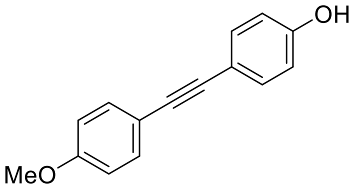

4-Iodophenol (440 mg, 2 mmol, 1 equiv.), 4-methoxyphenylacetylene (317 mg, 2.4 mmol, 1.2 equiv.). After removal of triethylamine crude product was dissolved in dichloromethane (30 mL) and transferred to a separatory funnel. Water (30 mL) was added and acidified to pH 6 with 2M HCl. Product was extracted with dichloromethane two more times (30 mL). Dichloromethane was dried over anhydrous sodium sulphate and removed under reduced pressure. Crude product was loaded onto Celite and purified with SiO_2 column chromatography using automated purification system Interchim puriFlash XS520 with petroleum ether/ethyl acetate as eluent. Product was isolated as an off-white crystalline solid, 408 mg, 91%.

^{1}H NMR ($CDCl_3$, 600 MHz) δ 7.46–7.43 (m, 2H), 7.42–7.39 (m, 2H), 6.88–6.85 (m, 2H), 6.81–6.78 (m, 2H), 4.89 (br, 1H), 3.83 (s, 3H). ^{1}H NMR data is in agreement with the literature.[24]

1-(Cyclohexylethynyl)-4-methoxybenzene (1f)

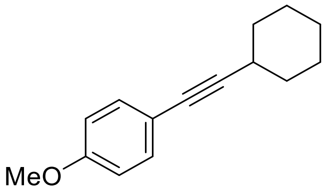

4-Iodoanisole (486 mg, 2 mmol, 1 equiv.), ethynylcyclohexane (260 mg, 314 µL, 2.4 mmol, 1.2 equiv.). Product was isolated as a yellow oil, 410 mg, 96%.

^{1}H NMR ($CDCl_3$, 600 MHz) δ 7.36–7.30 (m, 2H), 6.82–6.79 (m, 2H), 3.79 (s, 3H), 2.60–2.53 (m, 1H), 1.91–1.85 (m, 2H), 1.79–1.73 (m, 2H), 156–1.49 (m, 3H), 1.38–1.31 (m, 3H). ^{1}H NMR data is in agreement with the literature.[25]

5-((4-Fluorophenyl)ethynyl)benzo[d][1,3]dioxole (1g)

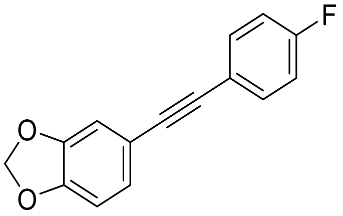

3,4-Methylenedioxybromobenzene (402 mg, 241 µL, 2 mmol, 1 equiv.), 4-fluorophenylacetylene (288 mg, 275 µL, 2.4 mmol, 1.2 equiv.). Reaction was preformed at 50 °C. Product was isolated as a white crystalline solid, 142 mg, 30%.

^{1}H NMR ($CDCl_3$, 600 MHz) δ 7.50–7.45 (m, 2H), 7.06–7.01 (m, 3H), 6.96 (d, *J* = 1.6 Hz, 1H), 6.79 (d, *J* = 8.0 Hz, 1H), 5.99 (s, 2H).

^{13}C{^{1}H} NMR ($CDCl_3$, 150 MHz) δ 163.4, 161.7, 148.1, 147.7, 133.5, 126.4, 119.6, 116.5, 115.9, 115.7, 111.7, 108.7, 101.5, 89.1, 86.9.

HRMS–ESI (*m/z*): $[M + H]^+$ calcd for $C_{15}H_{10}FO_2^+$, 241.0659; found, 241.0663.

2-Chloro-1-((4-chlorophenyl)ethynyl)-4-nitrobenzene (1h)

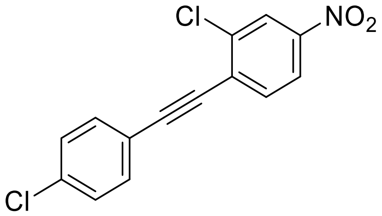

2-Chloro-4-nitroiodobenzene (567 mg, 2 mmol, 1 equiv.), 4-chlorophenylacetylene (328 mg, 2.4 mmol, 1.2 equiv.). Product was isolated as a yellow crystalline solid, 572 mg, 98%.

^{1}H NMR ($CDCl_3$, 600 MHz) δ 8.33 (s, 1H), 8.12 (d, *J* = 8.7 Hz, 1H), 7.70 (d, *J* = 8.7 Hz, 1H), 7.55–7.51 (m, 2H), 7.40–7.37 (m, 2H).

^{13}C{^{1}H} NMR ($CDCl_3$, 150 MHz) δ 147.4, 137.1, 136.1, 133.9, 133.6, 133.4, 129.8, 129.2, 129.1, 124.8, 121.8, 120.5, 98.9, 85.9.

HRMS–ESI (*m/z*): $[M + H]^+$ calcd for $C_{14}H_8Cl_2NO_2^+$, 291.9932; found, 291.9925.

1-(6-chlorohex-1-yn-1-yl)-4-nitrobenzene (1j)

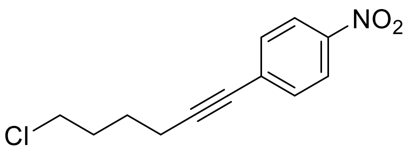

4-Iodonitrobenzene (498 mg, 2 mmol, 1 equiv.), 6-chloro-1-hexyne (280 mg, 291 µL, 2.4 mmol, 1.2 equiv.). Product was isolated as a yellow oil, 391 mg, 82%.

^{1}H NMR ($CDCl_3$, 600 MHz) δ 8.18–8.14 (m, 2H), 7.53–7.49 (m, 2H), 3.61 (t, *J* = 6.5 Hz, 2H), 2.51 (m, 2H), 2.00–1.93 (m, 2H), 1.83–1.76 (m, 2H).

^{13}C{^{1}H} NMR ($CDCl_3$, 150 MHz) δ 146.9, 132.5, 131.0, 123.7, 95.6, 80.0, 44.6, 31.8, 25.8, 19.1.

HRMS–ESI (*m/z*): $[M + H]^+$ calcd for $C_{12}H_{13}ClNO_2^+$, 238.0629; found, 238.0628.

4-(Naphthalen-1-ylethynyl)benzaldehyde (1k)

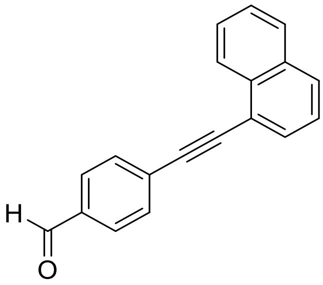

1-Iodonaphthalene (508 mg, 292 µL, 2 mmol, 1 equiv.), 4-ethynylbenzaldehyde (312 mg, 2.4 mmol, 1.2 equiv.). Product was isolated as an off-white crystalline solid, 501 mg, 98%.

^{1}H NMR ($CDCl_3$, 600 MHz) δ 10.05 (s, 1H), 8.43–8.40 (m, 1H), 7.93–7.88 (m, 4H), 7.81–7.78 (m, 3H), 7.63 (ddd, J = 8.3, 6.8, 1.3 Hz, 1H), 7.56 (ddd, J = 8.1, 6.8, 1.3 Hz, 1H), 7.49 (dd, J = 8.3, 7.1 Hz, 1H). ^{1}H NMR data is in agreement with the literature.[26]

4-((4-hydroxy-3-methoxyphenyl)ethynyl)-2,6-dimethoxyphenol (1n)

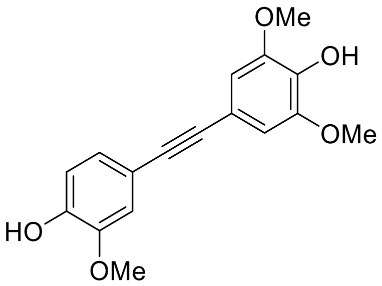

4-iodo-2,6-dimethoxyphenol (**4a**) (210 mg, 0.75 mmol, 1 equiv.), 4-ethynyl-2-methoxyphenol (**3a**) (134 mg, 0.9 mmol, 1.2 equiv.), $PdCl_2(PPh_3)_2$ (26 mg, 0.0375 mmol, 5 mol%), CuI (7.1 mg, 0.0375 mmol, 5 mol%). After removal of triethylamine crude product was dissolved in ethyl acetate (30 mL) and transferred to a separatory funnel. Water (30 mL) was added and acidified to pH 2 with 2M HCl. Product was extracted with ethyl acetate two more times (30 mL). Ethyl acetate was dried over anhydrous sodium sulphate and removed under reduced pressure. Crude product was loaded onto Celite and purified with SiO_2 column chromatography using automated purification system Interchim puriFlash XS520 with petroleum ether/ethyl acetate as eluent. Product isolated as a brown solid, 171 mg, 76%.

^{1}H NMR ($CDCl_3$, 600 MHz) δ 7.08 (dd, J = 8.2, 1.8 Hz, 1H), 7.02 (d, J = 1.8 Hz, 1H), 6.88 (d, J = 8.2 Hz, 1H), 6.77 (s, 2H), 5.74 (s, 1H), 3.91 (m, 9H).

$^{13}C\{^1H\}$ NMR ($CDCl_3$, 150 MHz) δ 147.0, 146.4, 146.3, 135.7, 125.6, 115.1, 114.7, 114.2, 113.9, 108.6, 88.0, 87.9, 56.5, 56.2.

HRMS–ESI (m/z): $[M + H]^+$ calcd. for $C_{17}H_{17}O_5^+$, 301.1071; found, 301.1075.

2.7. Synthesis of other precursors

Synthesis of 4-iodo-2,6-dimethoxyphenol (4a)

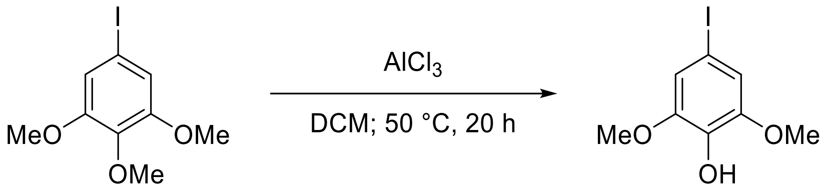

4-Iodo-2,6-dimethoxyphenol (**4a**) was prepared according to modified literature procedure.[27] 100 mL Schlenk flask was attached to schlenk line, heat-gun dried under vacuum and backfilled with argon three times. Under flow of argon were added 5-iodo-1,2,3-trimethoxybenzene (882 mg, 3 mmol, 1 equiv.), 30 mL of dry dichloromethane and anhydrous aluminum chloride ($AlCl_3$, 4.5 mmol, 1.5 equiv.). After addition of $AlCl_3$ flask was equipped with reflux condenser and stirred at 50 °C under argon for 20 hours. Reaction mixture was then cooled to room temperature and quenched with 30 mL of 2M HCl. Product was extracted into dichloromethane (3 x 30 mL). Organic phase was dried over anhydrous sodium sulphate and removed under reduced pressure. Crude product was purified using silica gel column chromatography (InterChim puriFlash XS520 Plus) using petroleum ether/ethyl acetate as eluent. Product was isolated as a yellow solid, 389 mg, 46%.

^{1}H NMR ($CDCl_3$, 600 MHz) δ 6.88 (s, 2H), 5.48 (s, 1H), 3.87 (s, 6H).

Synthesis of 2-methoxy-4-((trimethylsilyl)ethynyl)phenol

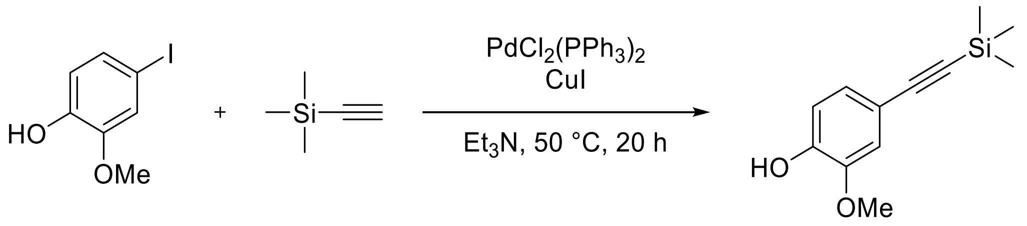

Oven-dried 50 mL reaction flask was equipped with a septum and purged with nitrogen. Under flow of nitrogen were added in the following order: 10 mL of anhydrous triethylamine (Et_3N), bis(triphenylphosphine)palladium chloride ($PdCl_2(PPh_3)_2$, 112 mg, 0.16 mmol, 4 mol%), copper(I) iodide (CuI, 38 mg, 0.2 mmol, 5 mol%), 4-iodo-2-methoxyphenol (1000 mg, 4 mmol, 1 equiv.) and trimethylsilylacetylene (692 µL, 491 mg, 5 mmol, 1.2 equiv.). Flask was equipped with nitrogen balloon and stirred overnight at 50 °C. After 20 hours triethylamine was removed under reduced pressure. Residue was transferred into separatory funnel using 50 mL of dichloromethane. 50 mL of water was added and acidified with 2M HCl to pH 3. Product was extracted into dichloromethane two more times (3 x 50 mL). Organic phases were combined and dried over anhydrous sodium sulphate. Dichloromethane was removed under reduced pressure and the crude product was purified with silica

gel column chromatography (InterChim puriFlash XS520 Plus) using petroleum ether/ethyl acetate as eluent. Product was isolated as a yellow crystalline solid, 821 mg, 93%.

^{1}H NMR ($CDCl_3$, 600 MHz) δ 7.03 (dd, J = 8.2, 1.8 Hz, 1H), 6.96 (d, J = 1.8 Hz, 1H), 6.83 (d, J = 8.2 Hz, 1H), 5.75 (s, 1H), 3.88 (s, 3H), 0.24 (s, 9H).

^{13}C{^{1}H} NMR ($CDCl_3$, 150 MHz) δ 146.6, 146.2, 126.3, 114.9, 144.6, 114.3, 105.5, 92.1, 56.2, 0.25.

HRMS–ESI (*m/z*): $[M + H]^+$ calcd for $C_{12}H_{17}O_2Si^+$, 221.0992; found, 221.0992.

Synthesis of 4-ethynyl-2-methoxyphenol (3a)

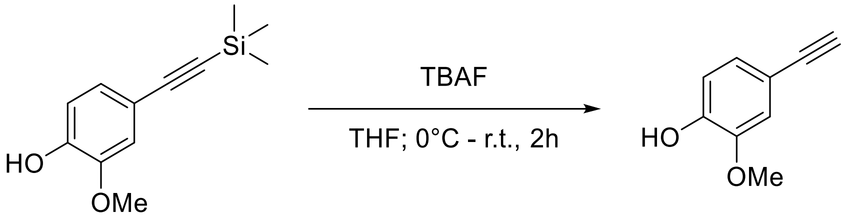

50 mL Schlenk flask was attached to Schlenk line, heat-gun dried and backfilled with argon three times. Then, under flow of argon 2-methoxy-4-((trimethylsilyl)ethynyl)phenol (440 mg, 2 mmol, 1 equiv.) was added, followed by 6 mL of dry tetrahydrofuran. Reaction flask was cooled to 0 °C using ice bath. Then tetrabutylammonium fluoride (TBAF, 6 mL of 1M THF solution, 6 mmol, 3 equiv.) was added dropwise over 5 minutes. Reaction was stirred at 0 °C and monitored with TLC. After 2 h no starting material was observed. THF was removed under reduced pressure and theresidue was transferred into separatory funnel with ethyl acetate (30 mL), 30 mL of water was added and mixture was acidified with 2M HCl to pH 3. Product was extracted into ethyl acetate two more times (3 x 30 mL). Organic phases were combined and dried over anhydrous sodium sulphate. Ethyl acetate was removed under reduced pressure using rotary evaporator. Crude product was NMR pure after extraction and was used as such in the next reaction. Purple oil, 250 mg, 84%.

^{1}H NMR ($CDCl_3$, 600 MHz) δ 7.05 (dd, J = 8.1, 1.8 Hz, 1H), 6.98 (d, J = 1.8 Hz, 1H), 6.85 (d, J = 8.1 Hz, 1H), 5.75 (s, 1H), 3.89 (s, 3H), 2.98 (s, 1H).

^{13}C{^{1}H} NMR ($CDCl_3$, 150 MHz) δ 146.8, 146.3, 126.3, 114.7, 114.5, 113.7, 84.1, 75.4, 56.1.

HRMS–ESI (*m/z*): $[M + H]^+$ calcd for $C_9H_9O_2^+$, 149.0597; found, 149.0599.

Synthesis of 2-methoxyphenyl acetate

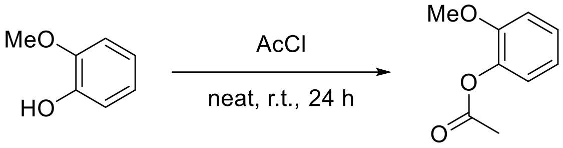

2-Methoxyphenyl acetate was prepared according to the literature procedure.[28] Acetyl chloride (1021 mg, 930 µL, 13 mmol, 1.3 equiv.) was added to 2-methoxyphenol (guaiacol, 1241 mg, 1118 µL, 10 mmol, 1 equiv.) and stirred overnight at room temperature. Product was extracted into dichloromethane (3 x 30 mL) from water (30 mL) which was neutralized with Na_2CO_3 solution. Dichloromethane was dried over anhydrous magnesium sulphate and removed under reduced pressure. Product was obtained as a colourless liquid and was pure after extraction, 1570 mg, 94%.

^{1}H NMR ($CDCl_3$, 600 MHz) δ 7.23–7.18 (m, 1H), 7.04 (dd, J = 7.9, 1.7 Hz, 1H), 7.00–6.92 (m, 2H), 3.84 (s, 3H), 2.32 (s, 3H). ^{1}H NMR spectrum is in agreement with reports from literature.[27]

Synthesis of 5-iodo-2-methoxyphenol (4b)

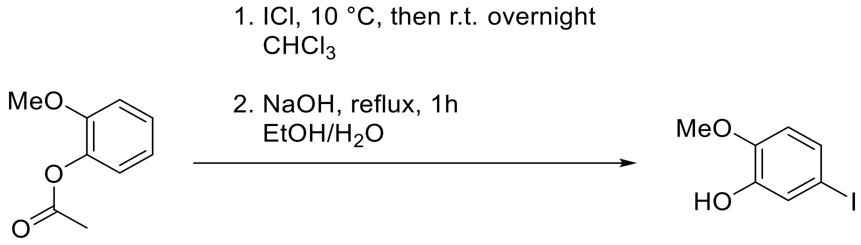

5-Iodo-2-methoxyphenol (**4b**) was prepared according to the procedure from the literature.[28] 2-(Methoxy)phenyl acetate (1.33 g, 8 mmol, 1 equiv.) was dissolved in 10 mL of chloroform and cooled to 10 °C. No precaution was taken to exclude air or moisture. Then iodine monochloride (ICl, 1572 mg, 486 µL, 9.7 mmol, 1.21 equiv.), dissolved in 4 mL of chloroform, was added dropwise while maintaining reaction temperature at 10 °C. After complete addition, the reaction was left to warm up to room temperature and stirred overnight. The reaction mixture was transferred into separatory funnel and washed with sodium metabisulfite until the iodine color disappeared. Chloroform was then removed under reduced pressure and the red-brown oil residue was dissolved in 3 mL of ethanol. Sodium hydroxide (1968 mg, 49.2 mmol, 6.15 equiv.) dissolved in 6 mL of ethanol/water mixture ($EtOH/H_2O$; 5/4) was added and the resulting mixture was refluxed for 1 hour. Then, it was placed into an ice bath and acidified with 40% aqueous H_2SO_4 solution to pH cca 2. Water was added and product was extracted into dichloromethane (3 x 50 mL). Dichloromethane was removed under reduced pressure and the crude product was purified with silica gel column chromatography (InterChim puriFlash XS520 Plus) using petroleum ether/ethyl acetate as eluent. Product was recrystallized using ethyl acetate/petroleum ether mixture to obtain 982 mg (49%) of a yellow crystalline solid.

^{1}H NMR ($CDCl_3$, 600 MHz) δ 7.23 (d, J = 2.1 Hz, 1H), 7.16 (dd, J = 8.4, 2.1 Hz, 1H), 6.60 (d, J = 8.4 Hz, 1H), 5.59 (s, 1H), 3.87 (s, 3H). ^{1}H NMR spectrum is in agreement with reports from literature.[28]

Synthesis of trimethyl-((3,4,5-trimethoxyphenyl)ethynyl)silane

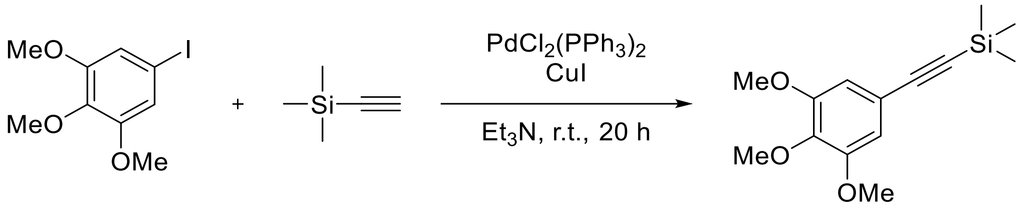

Oven-dried 50 mL flask was equipped with septum and purged with nitrogen. Under flow of nitrogen were added in the following order: 10 mL of anhydrous triethylamine (Et_3N), bis(triphenylphosphine)palladium chloride ($PdCl_2(PPh_3)_2$, 56 mg, 0.08 mmol, 2 mol%), copper(I) iodide (CuI, 23 mg, 0.12 mmol, 3 mol%), 3,4,5-trimethoxyiodobenzene (1176 mg, 4 mmol, 1 equiv.) and trimethylsilylacetylene (609 µL, 432 mg, 4.4 mmol, 1.1 equiv.). Reaction flask was equipped with a nitrogen balloon and stirred overnight at room temperature. After 20 hours, triethylamine was removed under reduced pressure. The residue was filtered through a silica plug using petroleum ether/ethyl acetate (1/1) mixture. Solvents were removed under reduced pressure and crude product was purified with silica gel column chromatography (InterChim puriFlash XS520 Plus) using petroleum ether/ethyl acetate as an Theproduct was isolated as a colourless oil which solidified overnight, 1015 mg, 96%. Product contains traces of starting aryl iodide.

^{1}H NMR ($CDCl_3$, 600 MHz) δ 6.70 (s, 2H), 3.85 (s, 6H), 3.84 (s, 3H), 0.25 (s, 9H). ^{1}H NMR spectrum is in agreement with reports from literature.[29]

Synthesis of 5-ethynyl-1,2,3-trimethoxybenzene (3b)

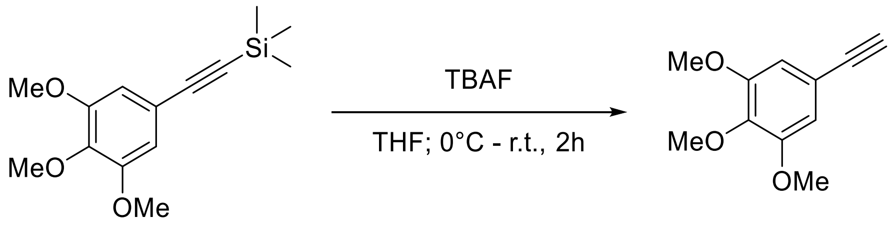

25 mL Schlenk flask was attached to Schlenk line, heat-gun dried and backfilled with argon three times. Then, under flow of argon, trimethyl-((3,4,5-trimethoxyphenyl)ethynyl)silane (792 mg, 3 mmol, 1 equiv.) was added, followed by 5 mL of dry tetrahydrofuran. Reaction mixture was cooled to 0 °C using ice bath. Then tetrabutylammonium fluoride (TBAF, 9 mL of 1M THF solution, 9 mmol, 3 equiv.) was added dropwise over 5 minutes. The reaction mixture was stirred at 0 °C for 30 minutes, warmed to room temperature and monitored with TLC. After 2 h no starting material was observed. THF was then

removed under reduced pressure and the crude product was purified with silica gel column chromatography (InterChim puriFlash XS520 Plus) using petroleum ether/ethyl acetate as an eluent. Product was isolated as a white solid, 463 mg, 80%.

^{1}H NMR ($CDCl_3$, 600 MHz) δ 6.72 (s, 2H), 3.85 (s, 3H), 3.84 (s, 6H), 3.03 (s, 1H). ^{1}H NMR spectrum is in agreement with reports from literature.[30]

Synthesis of 1-(4-iodophenyl)ethan-1-ol (4c)

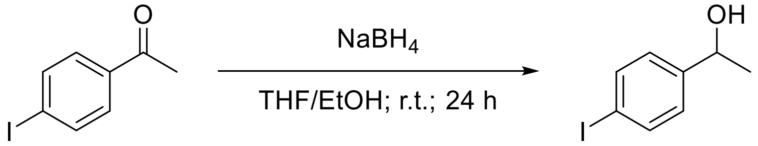

1-(4-Iodophenyl)ethan-1-ol (**4c**) was prepared according to the modified literature procedure.[31] 4-Iodoacetophenone (1230 mg, 5 mmol, 1 equiv.) was dissolved in 10 mL of anhydrous THF and added dropwise to stirring solution of $NaBH_4$ (95 mg, 2.5 mmol, 0.5 equiv.) in anhydrous ethanol (5 mL). After addition, the reaction mixture was left to stir overnight at room temperature. Then, the reaction mixture was poured into 50 mL of 2M HCl solution and extracted with diethyl ether (3 x 40 mL). Organic phases were combined and washed with saturated $NaHCO_3$ solution and brine. Diethyl ether was dried over anhydrous sodium sulphate and removed under reduced pressure. Crude product was loaded onto celite and purified using silica gel column chromatography (InterChim puriFlash XS520 Plus) using petroleum ether/ethyl acetate as eluent. Product was isolated as a yellow oil, 1080 mg, 87% (contains traces of impurity).

^{1}H NMR ($CDCl_3$, 600 MHz) δ 7.67 (d, J = 7.5 hz, 2H), 7.12 (d, J = 7.5 Hz, 2H), 4.85 (q, J = 6.5 Hz, 1H), 1.81 (br, 1H), 1.46 (d, J = 6.5 Hz, 3H). ^{1}H NMR spectrum is in agreement with reports from literature.[32]

3. COPIES OF NMR SPECTRA

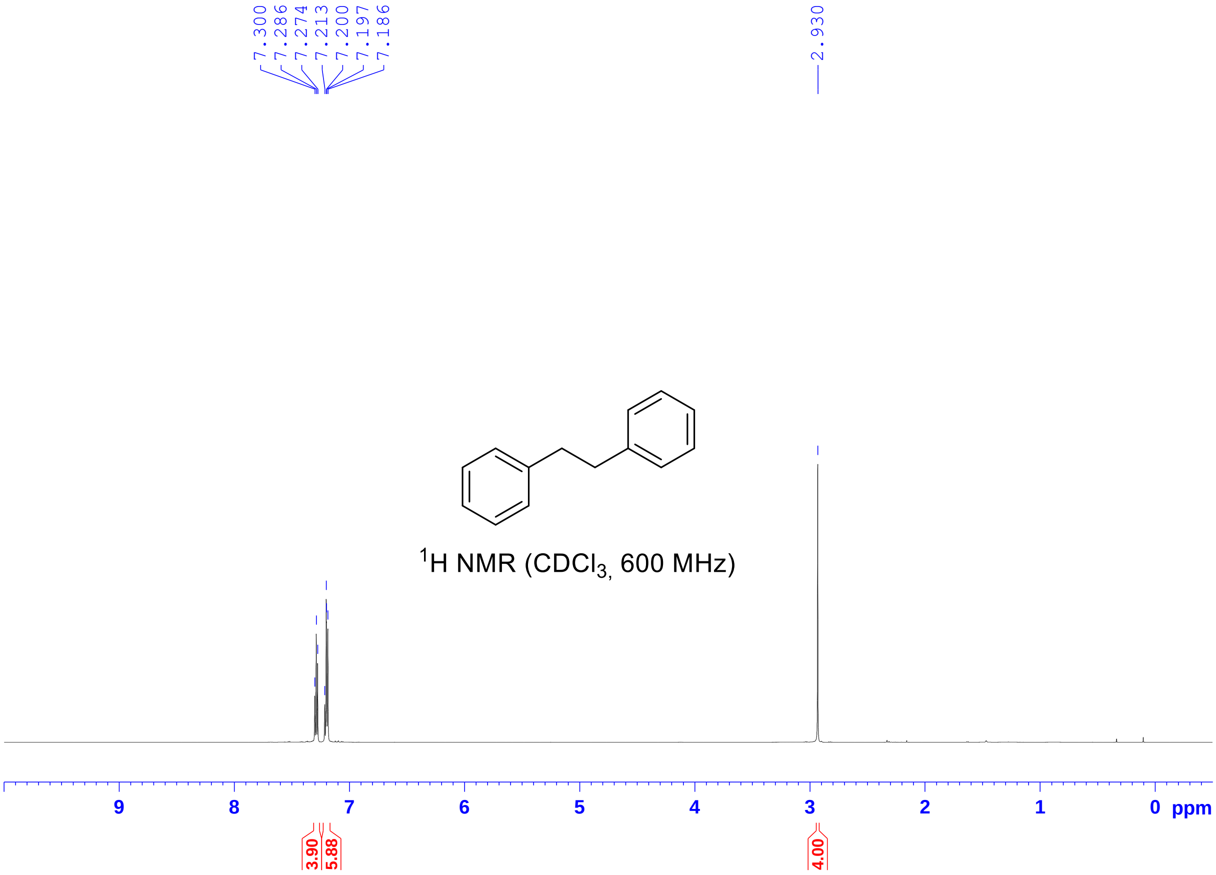

^{1}H NMR spectrum of 1,2-diphenylethane (**2a**) in $CDCl_3$.

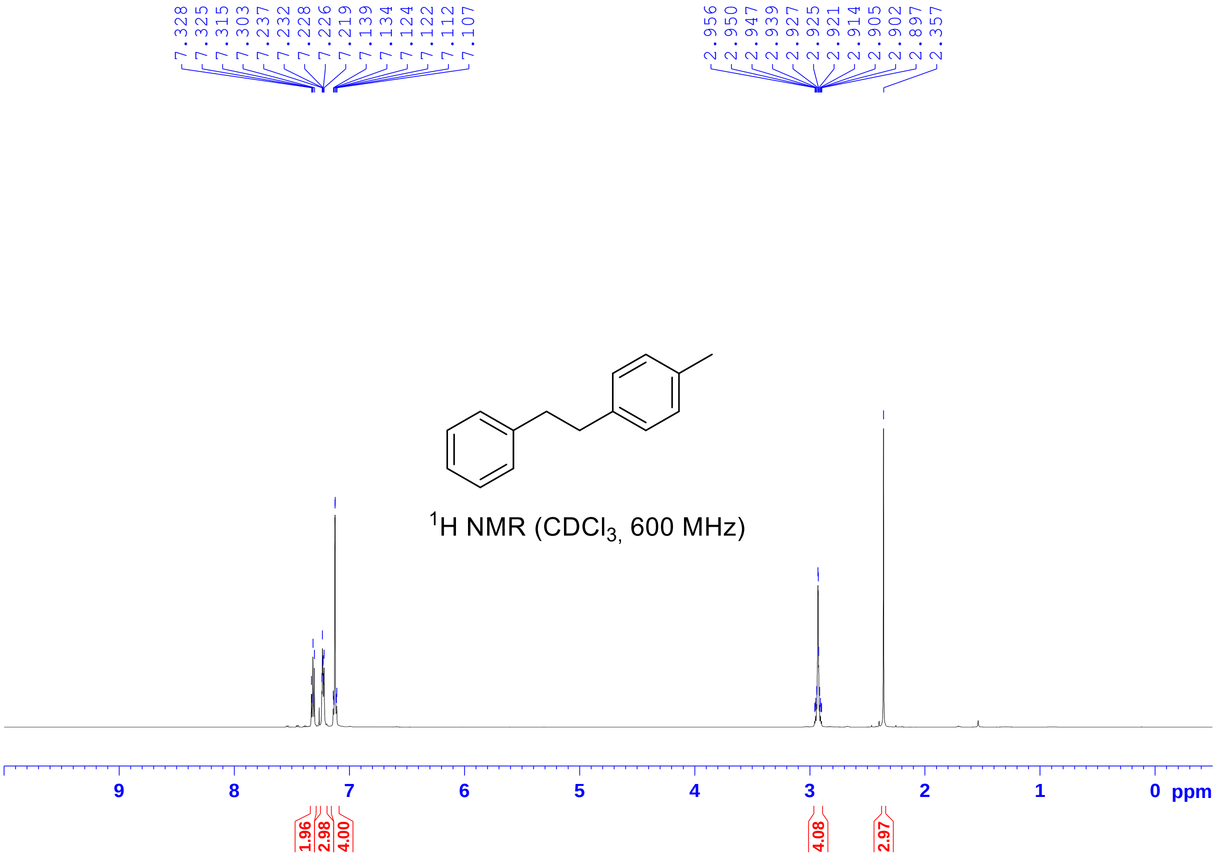

^{1}H NMR spectrum of 1-methyl-4-phenethylbenzene (**2b**) in $CDCl_3$.

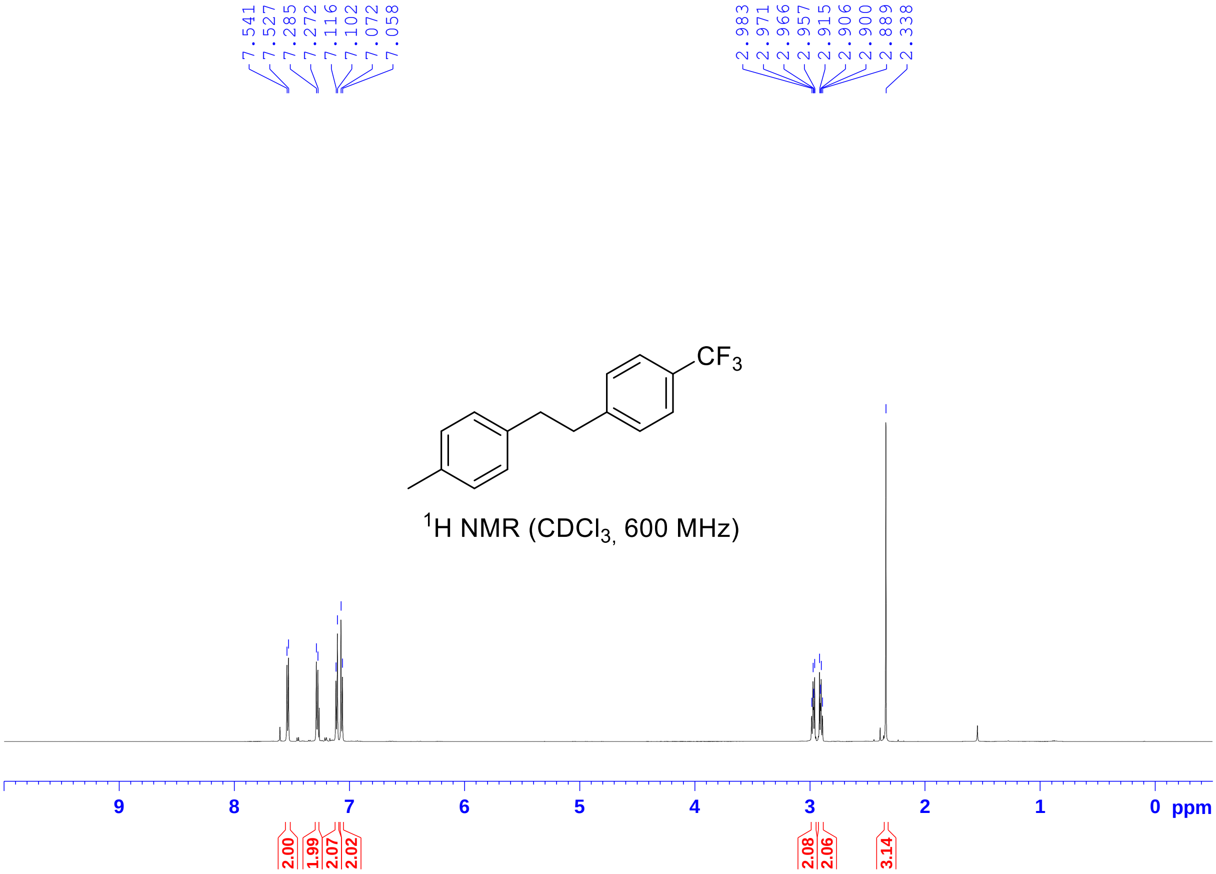

^{1}H NMR spectrum of 1-methyl-4-(4-(trifluoromethyl)phenethyl)benzene (**2c**) in $CDCl_3$.

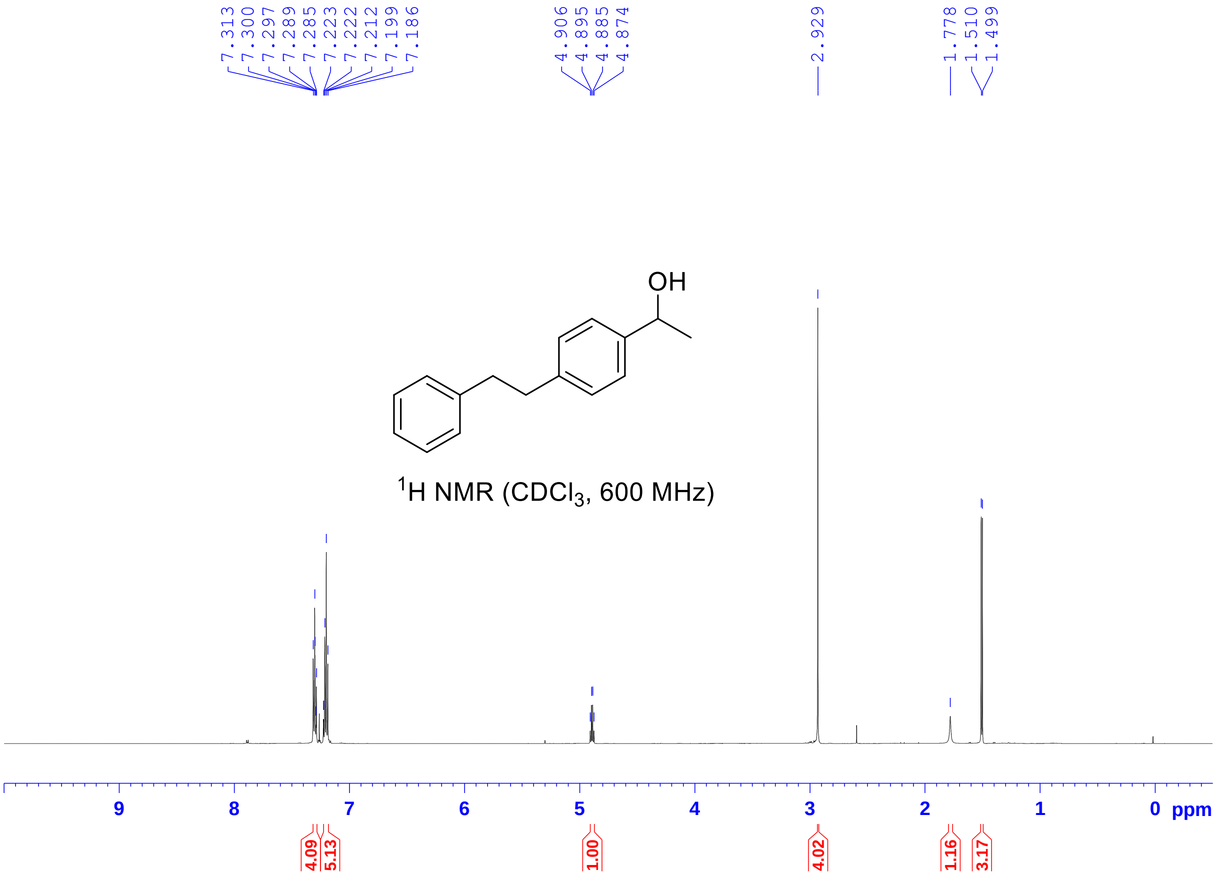

^{1}H NMR spectrum of 1-(4-phenethylphenyl)ethan-1-ol (**2d**) in $CDCl_3$.

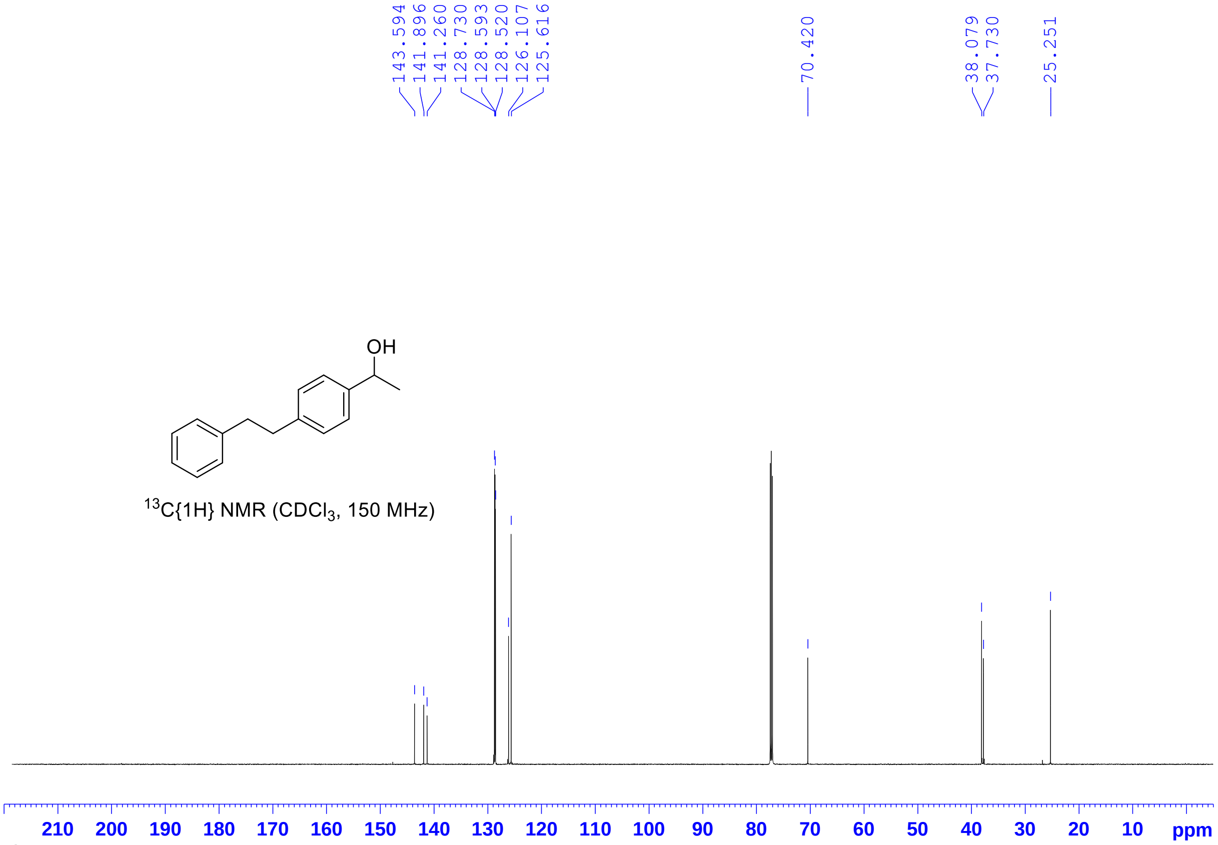

$^{13}C\{^{1}H\}$ NMR spectrum of 1-(4-phenethylphenyl)ethan-1-ol (**2d**) in $CDCl_3$.

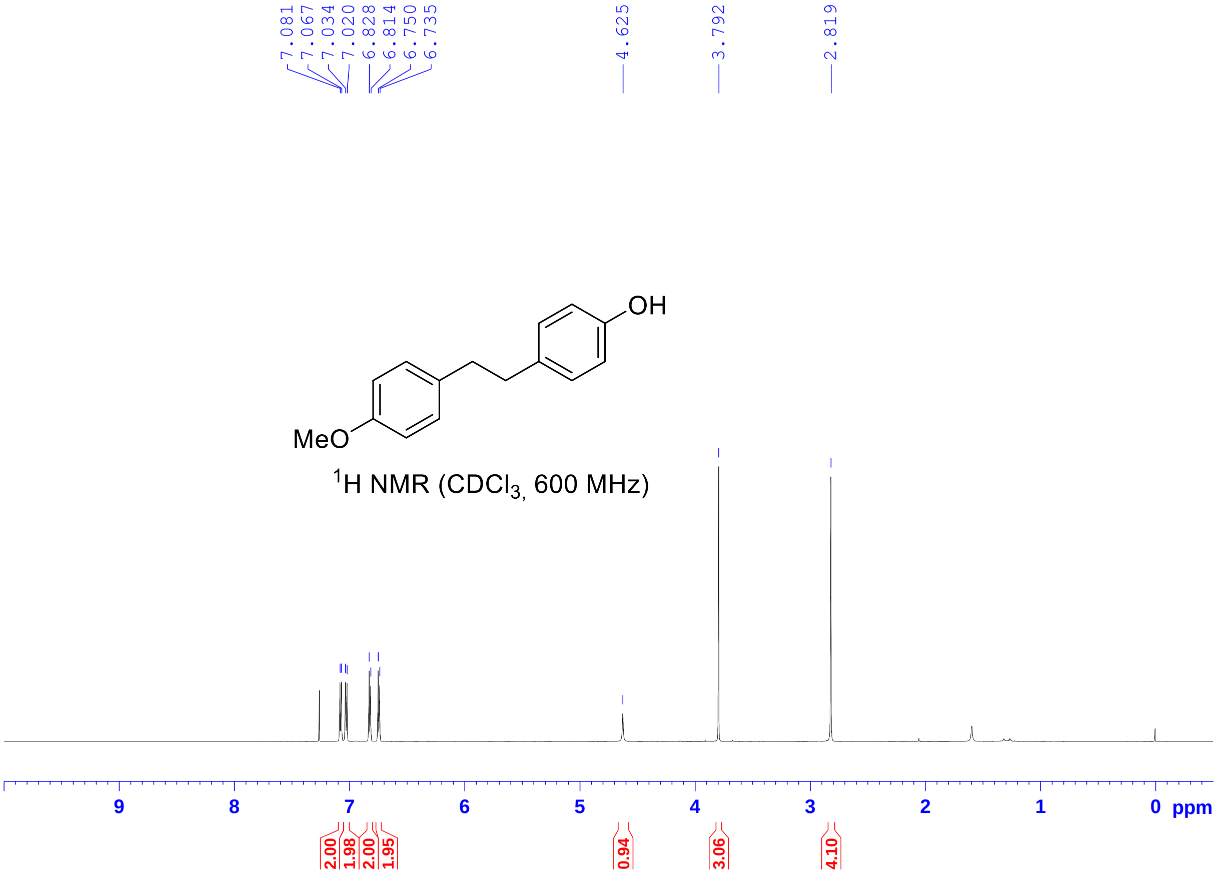

^{1}H NMR spectrum of 4-(4-methoxyphenethyl)phenol (**2e**) in $CDCl_3$.

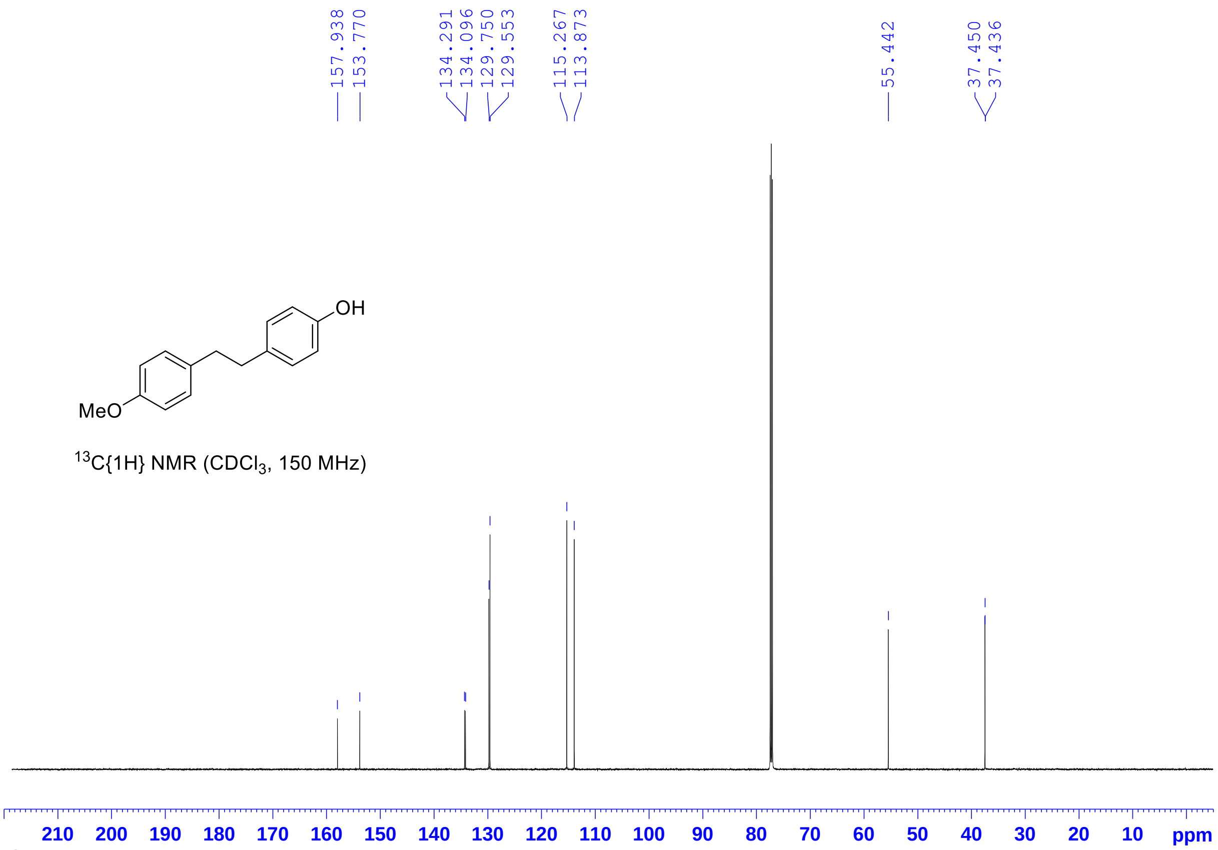

$^{13}C\{^1H\}$ NMR spectrum of 4-(4-methoxyphenethyl)phenol (**2e**) in $CDCl_3$.

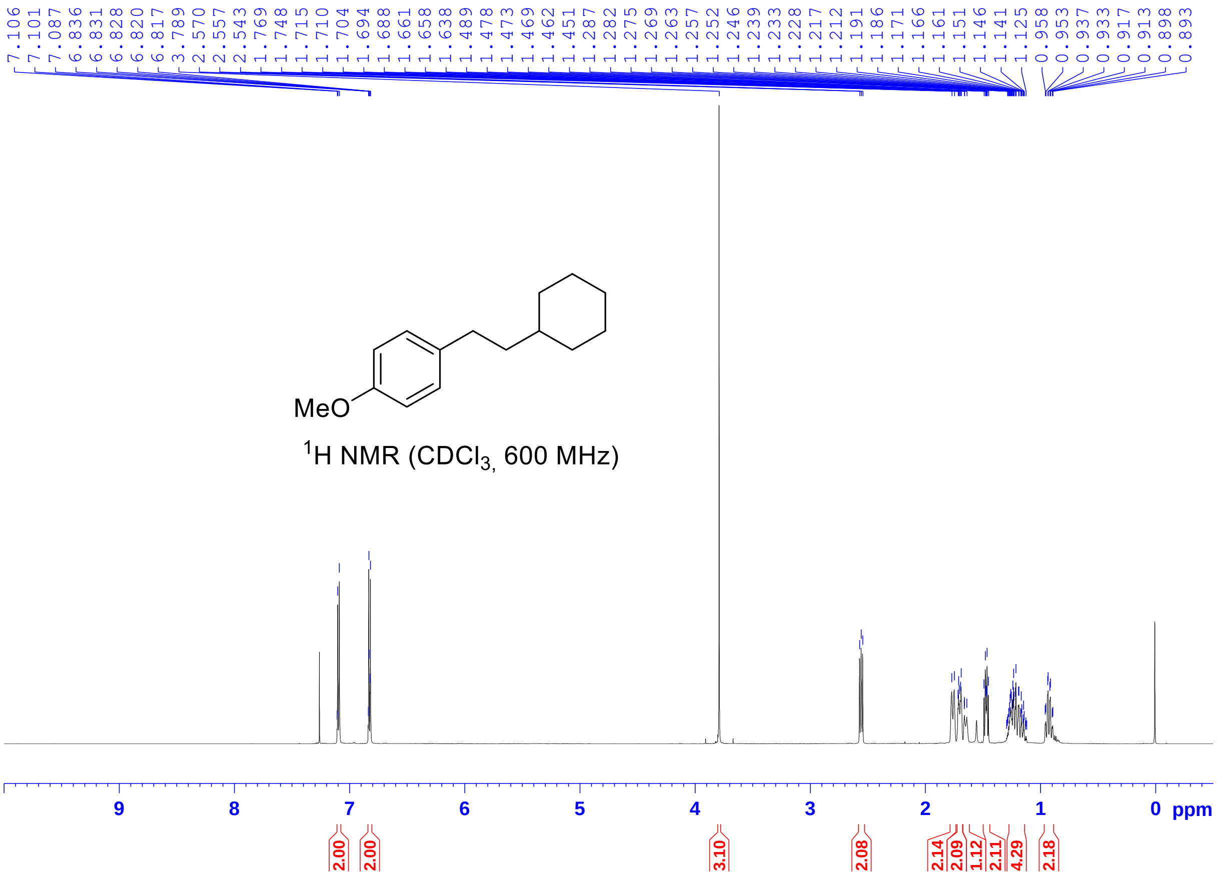

^1H NMR spectrum of 1-(2-Cyclohexylethyl)-4-methoxybenzene (**2f**) in $CDCl_3$.

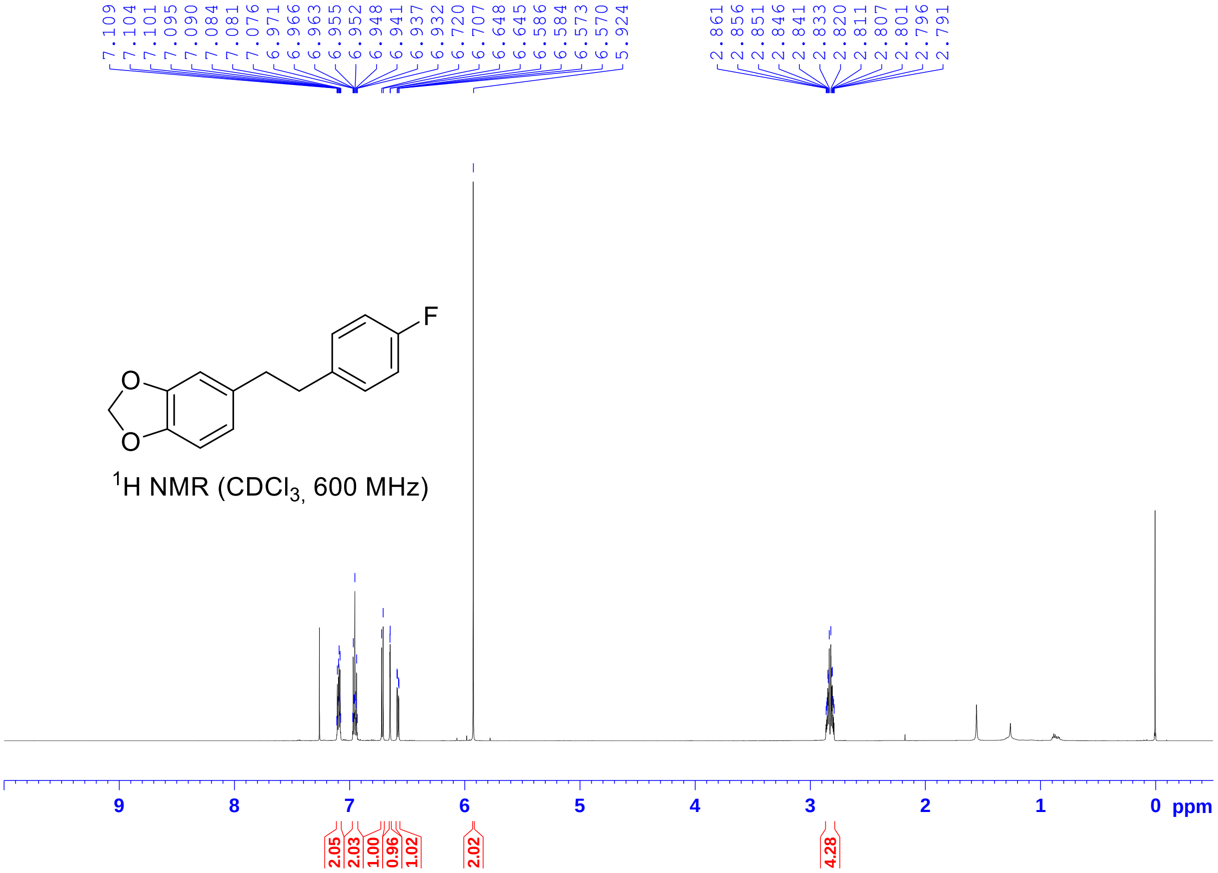

^{1}H NMR spectrum of 5-(4-Fluorophenethyl)benzo[d][1,3]dioxole (**2g**) in $CDCl_3$.

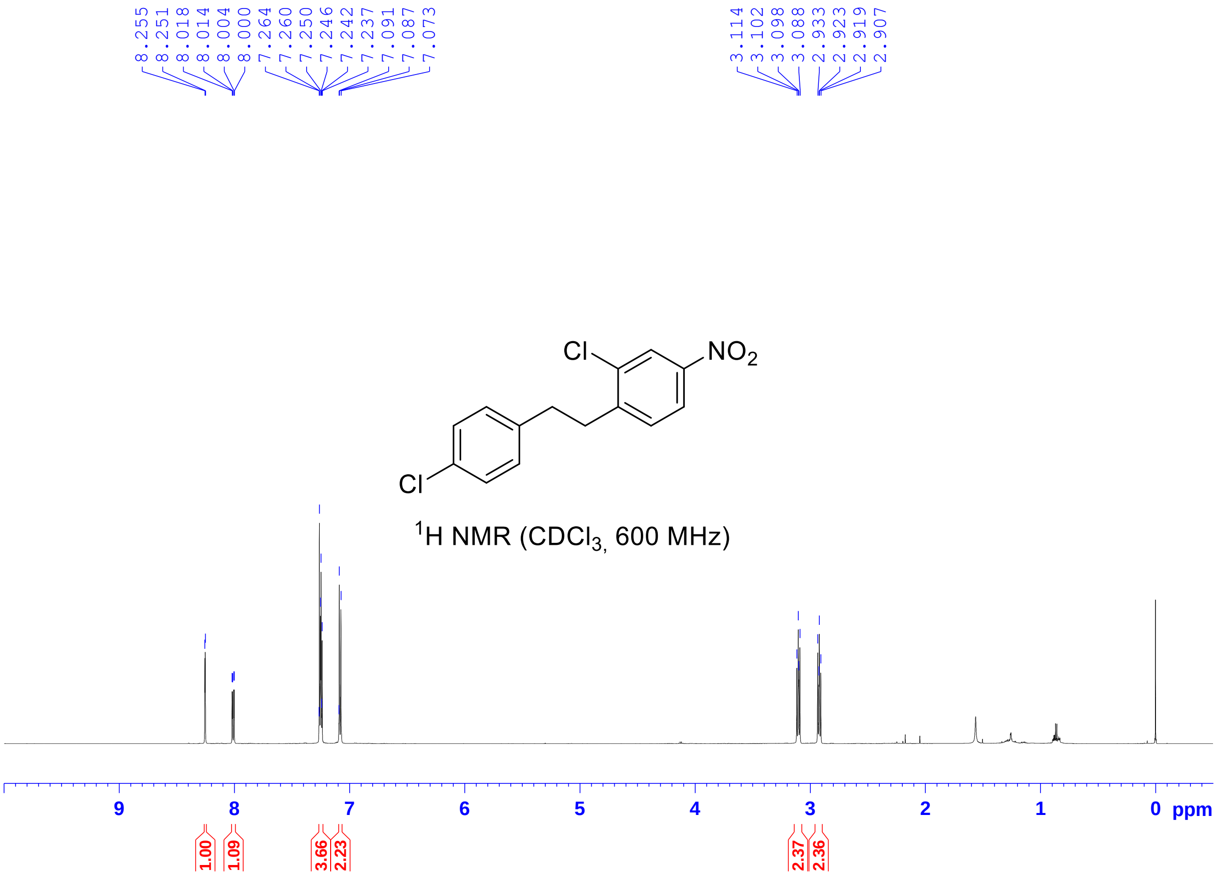

^{1}H NMR spectrum of 2-chloro-1-(4-chlorophenethyl)-4-nitrobenzene (**2h**) in $CDCl_3$. Aromatic signals at 7.26 ppm are overlapping with residual $CHCl_3$.

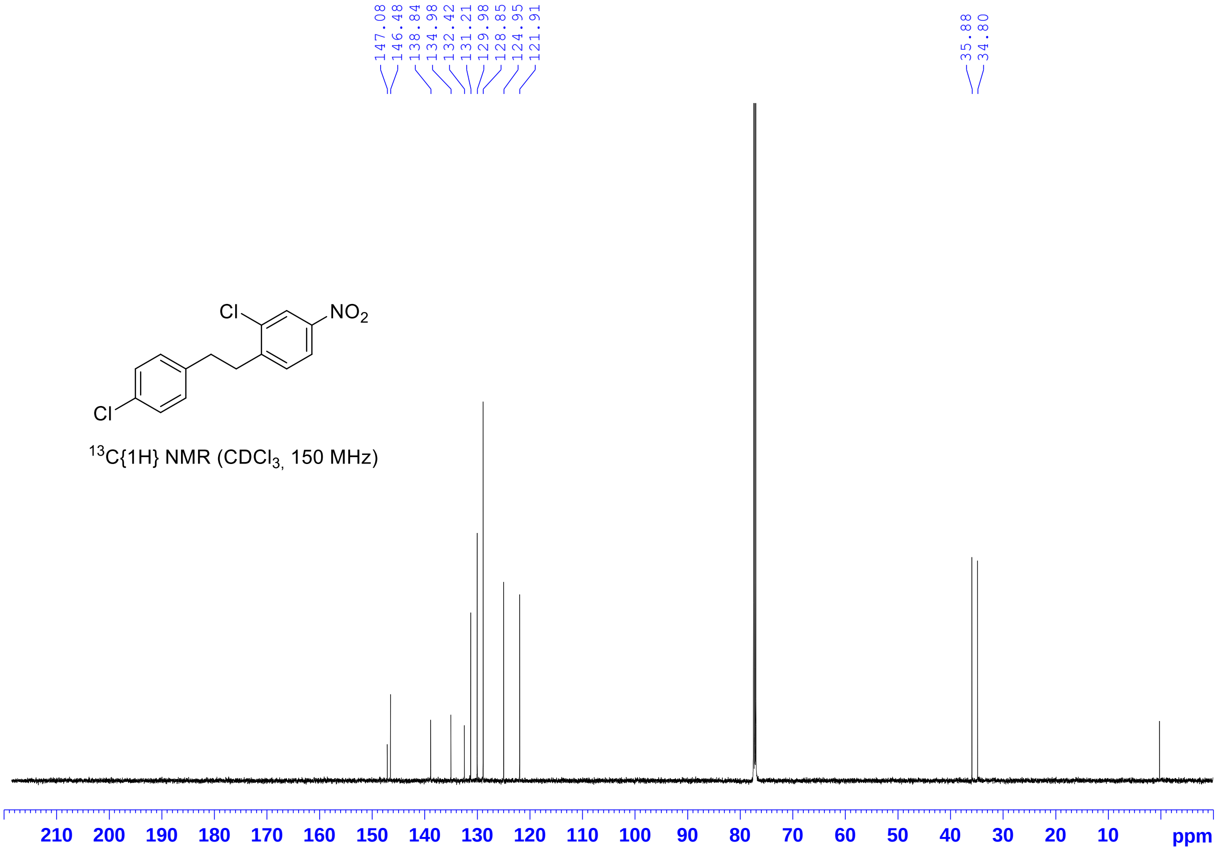

$^{13}C\{^{1}H\}$ NMR spectrum of 2-chloro-1-(4-chlorophenethyl)-4-nitrobenzene (**2h**) in $CDCl_3$.

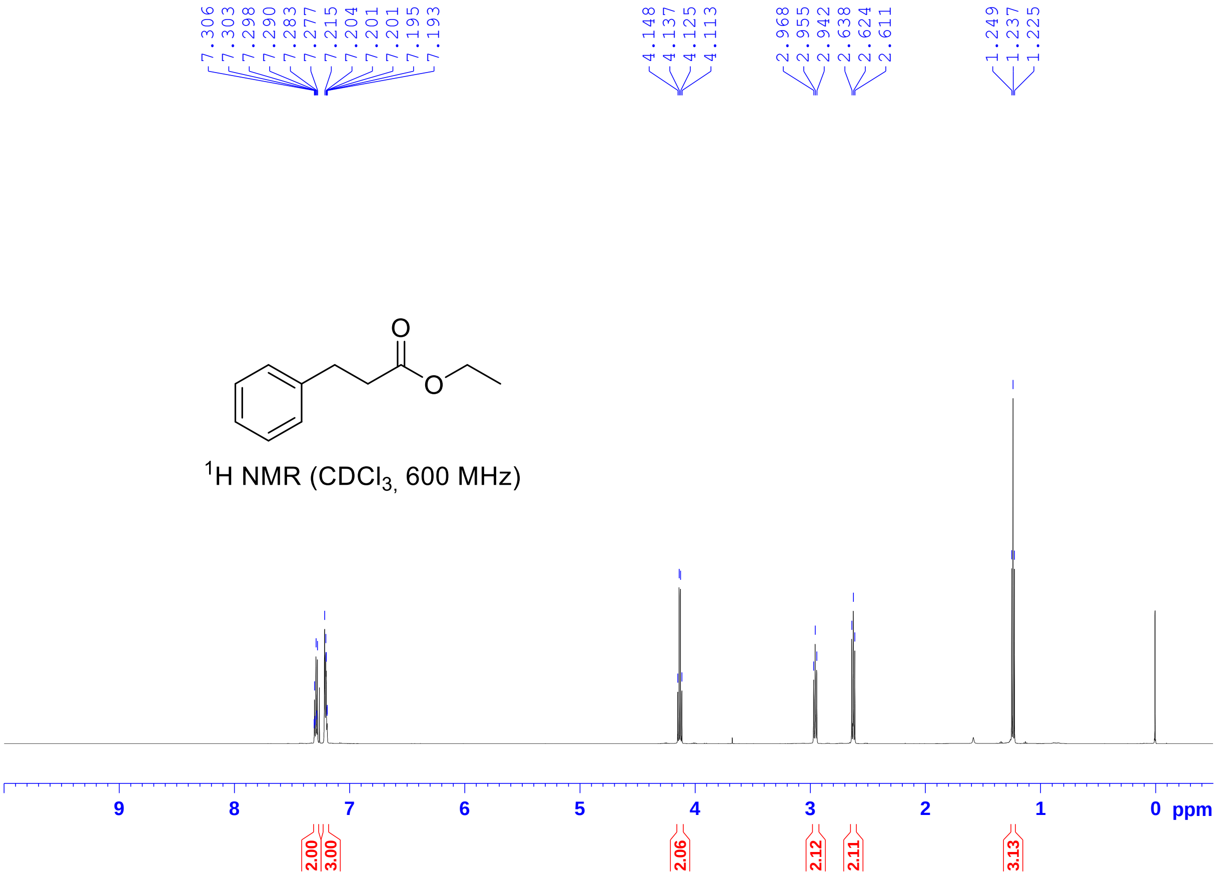

^{1}H NMR spectrum of ethyl 3-phenylpropanoate (**2i**) in $CDCl_3$.

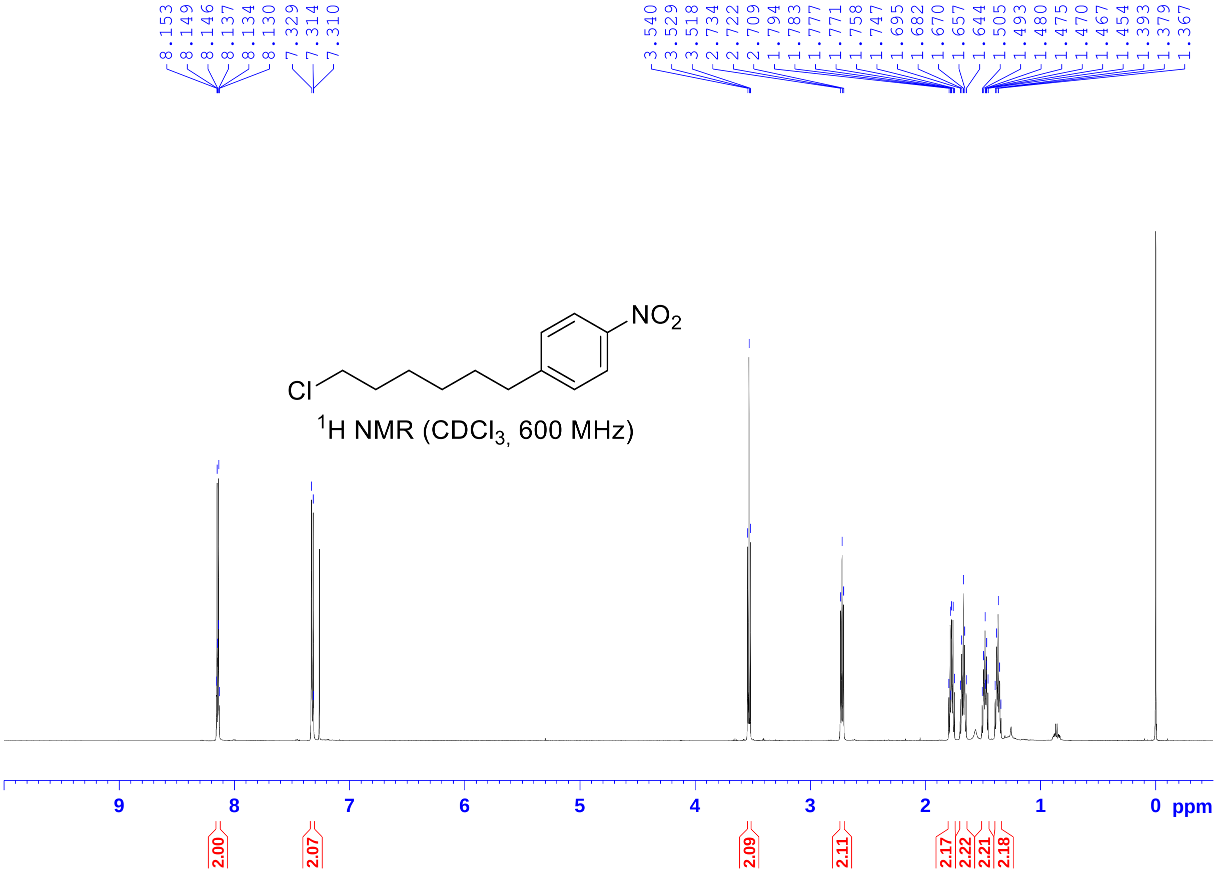

^{1}H NMR spectrum of 1-(6-Chlorohexyl)-4-nitrobenzene (**2j**) in $CDCl_3$.

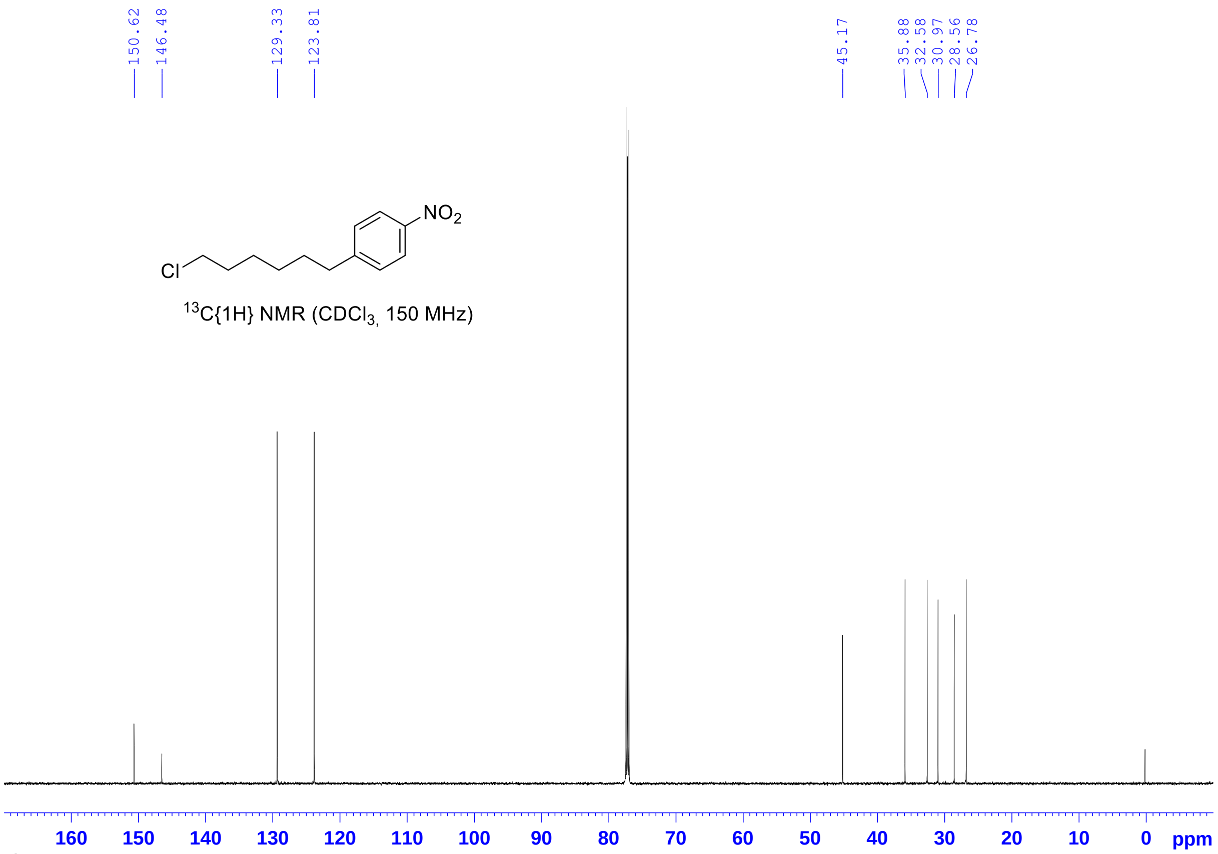

^{13}C{^{1}H} NMR spectrum of 1-(6-Chlorohexyl)-4-nitrobenzene (**2j**) in $CDCl_3$.

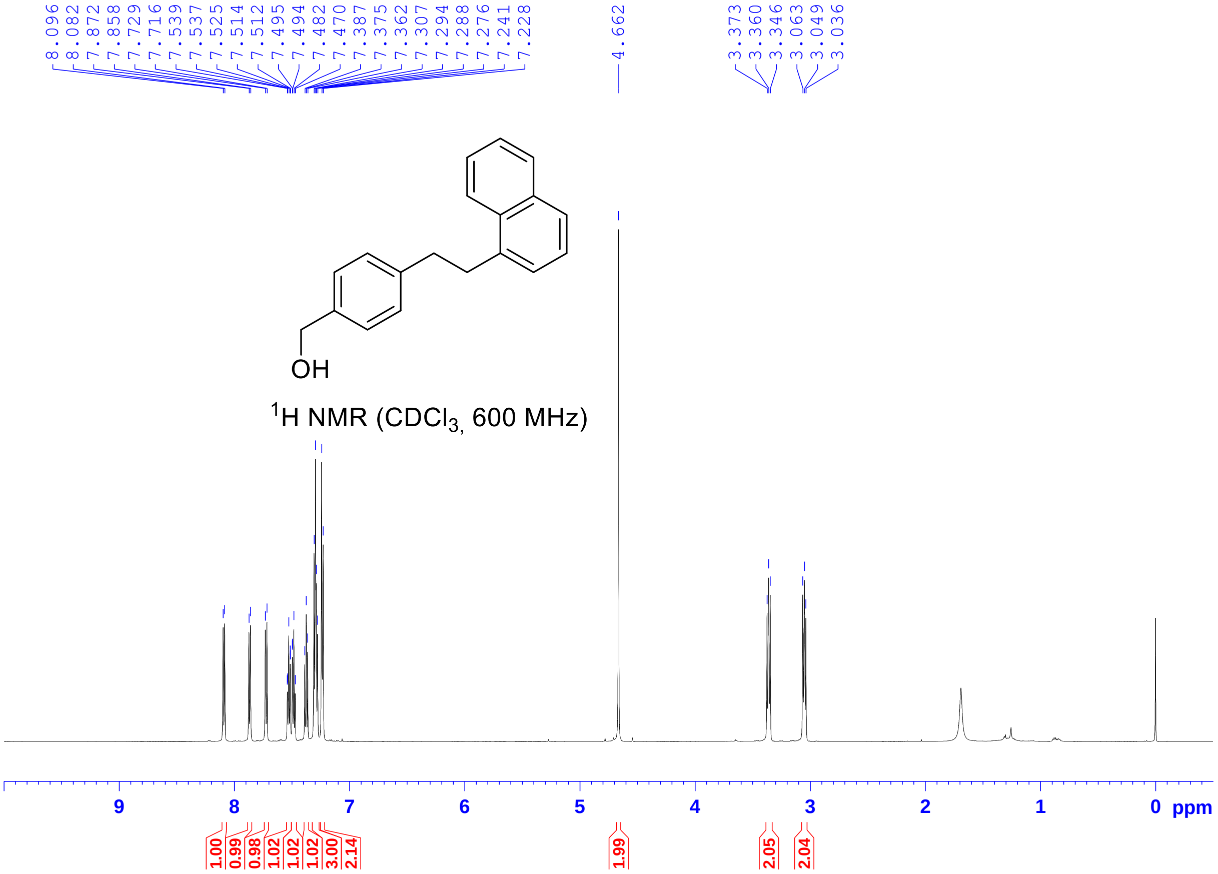

^{1}H NMR spectrum of (4-(2-(naphthalen-1-yl)ethyl)phenyl)methanol (**2k**) in $CDCl_3$.

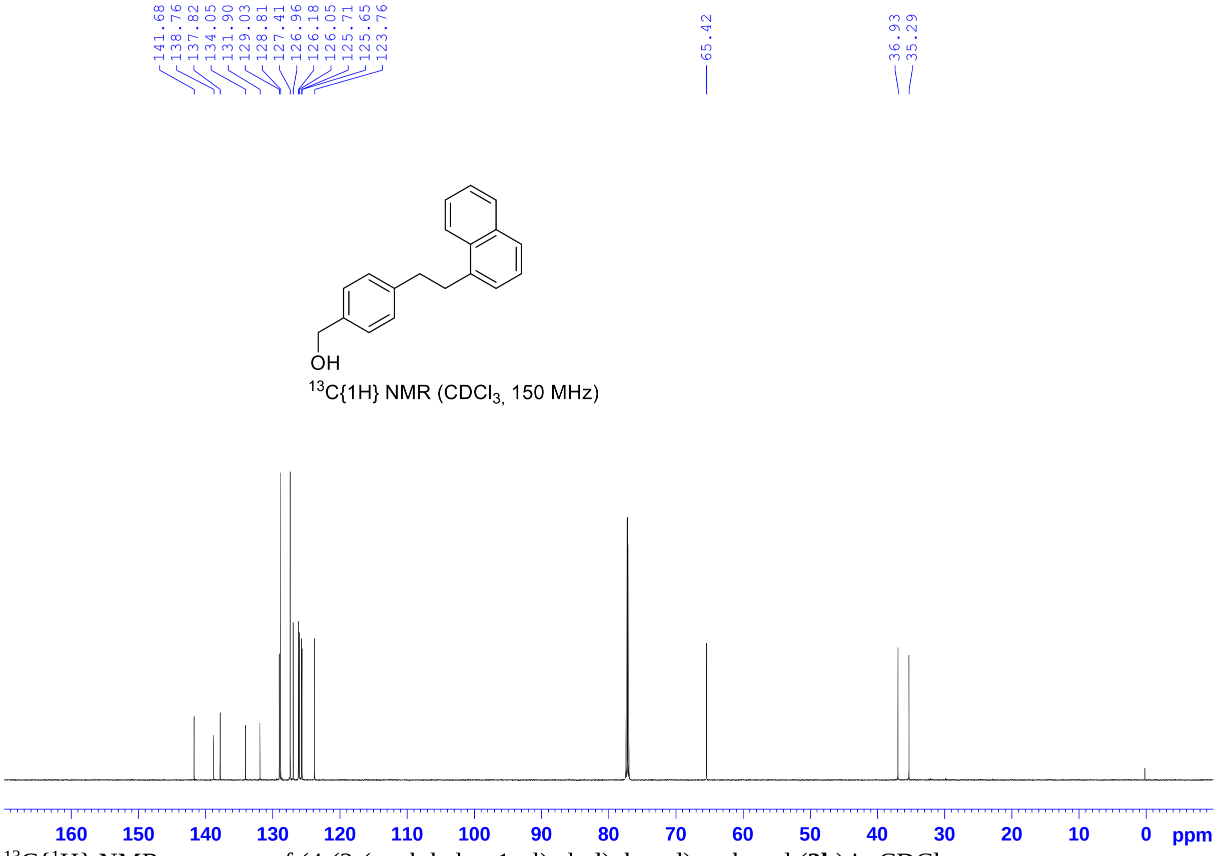

^{13}C{^{1}H} NMR spectrum of (4-(2-(naphthalen-1-yl)ethyl)phenyl)methanol (**2k**) in $CDCl_3$.

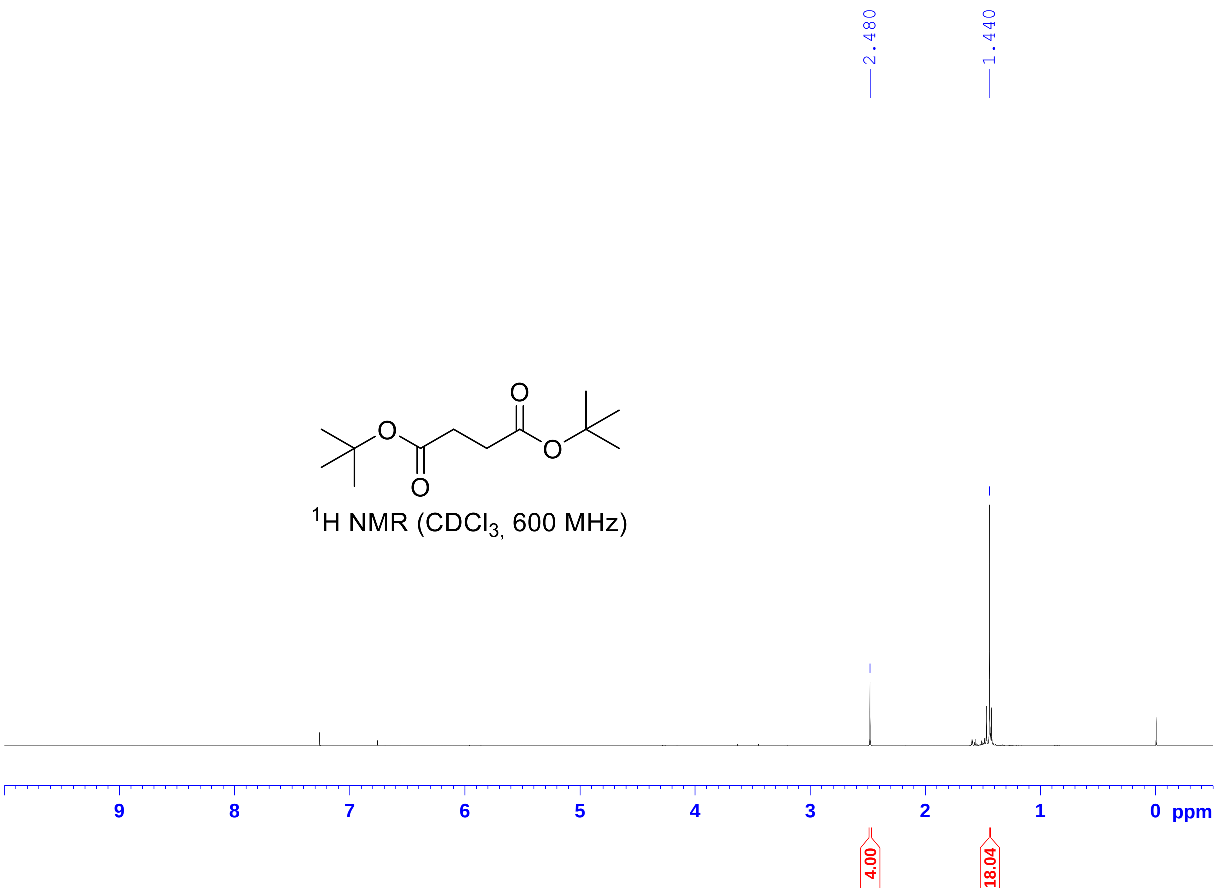

^{1}H NMR spectrum of di-*tert*-butyl succinate (**2l**) in $CDCl_3$.

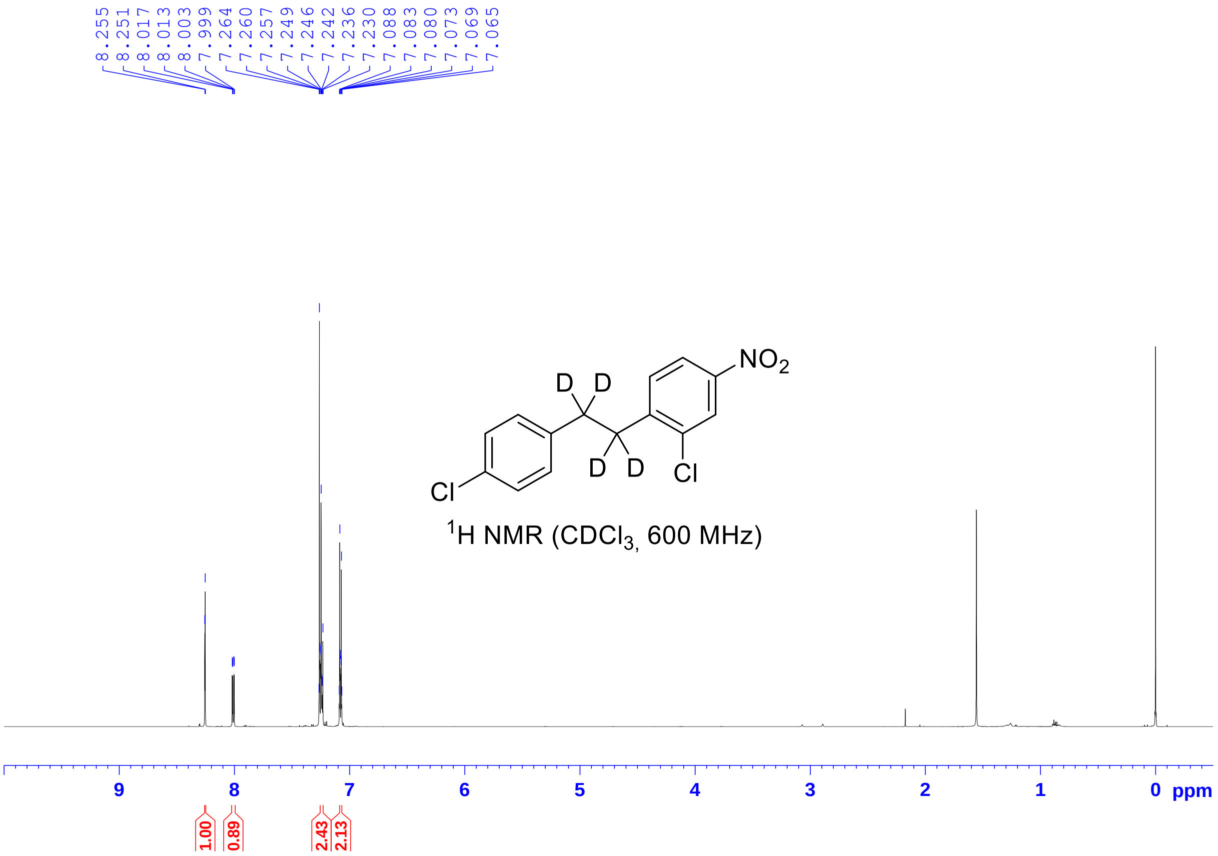

^{1}H NMR spectrum of 2-chloro-1-(2-(4-chlorophenyl)ethyl-1,1,2,2-d_4)-4-nitrobenzene (**2m**) in $CDCl_3$. Aromatic signals at 7.26 ppm are overlapping with residual $CHCl_3$.

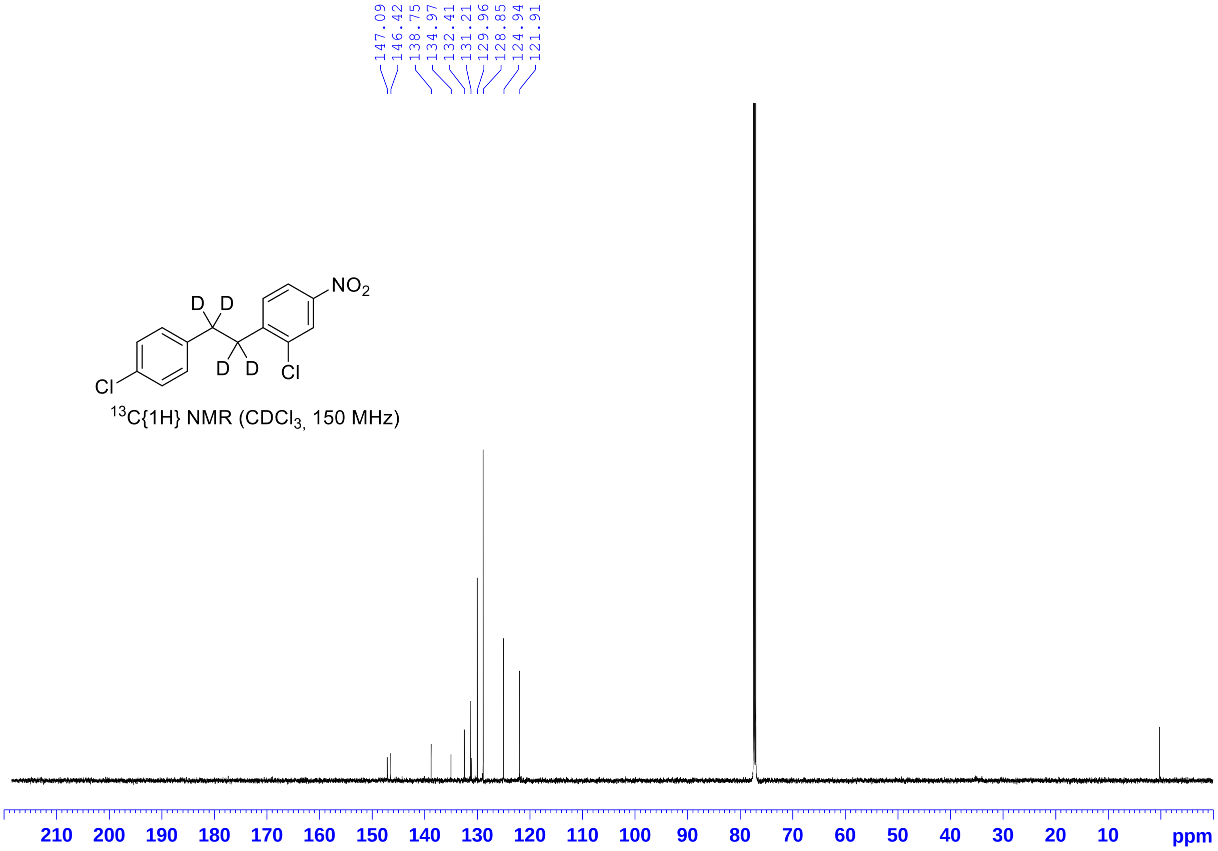

$^{13}C\{^{1}H\}$ NMR spectrum of 2-chloro-1-(2-(4-chlorophenyl)ethyl-1,1,2,2-d_4)-4-nitrobenzene (**2m**) in $CDCl_3$.

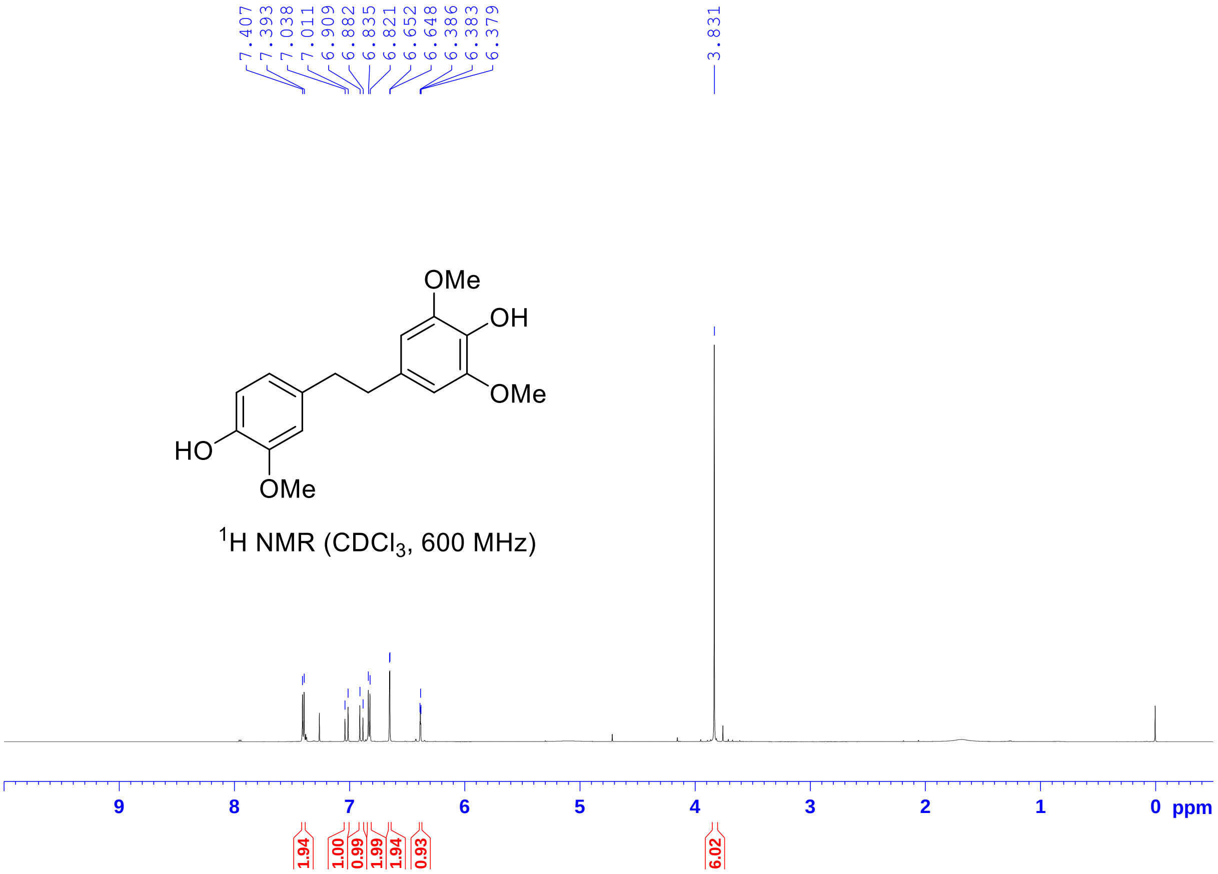

^{1}H NMR spectrum of 4-(4-hydroxy-3-methoxyphenethyl)-2,6-dimethoxyphenol (moscatilin) (**2n**) in $CDCl_3$.

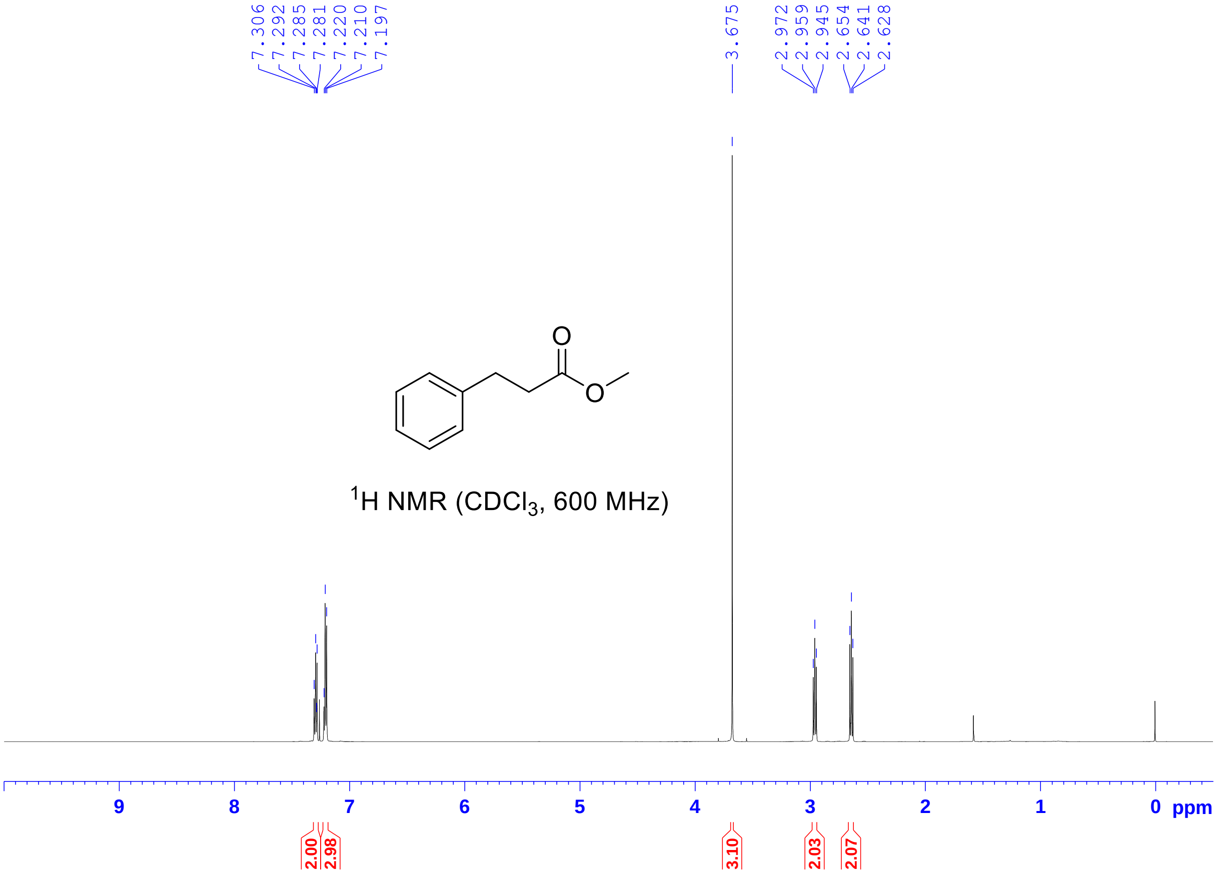

^{1}H NMR spectrum of methyl 3-phenylpropanoate (**2o**) in $CDCl_3$.

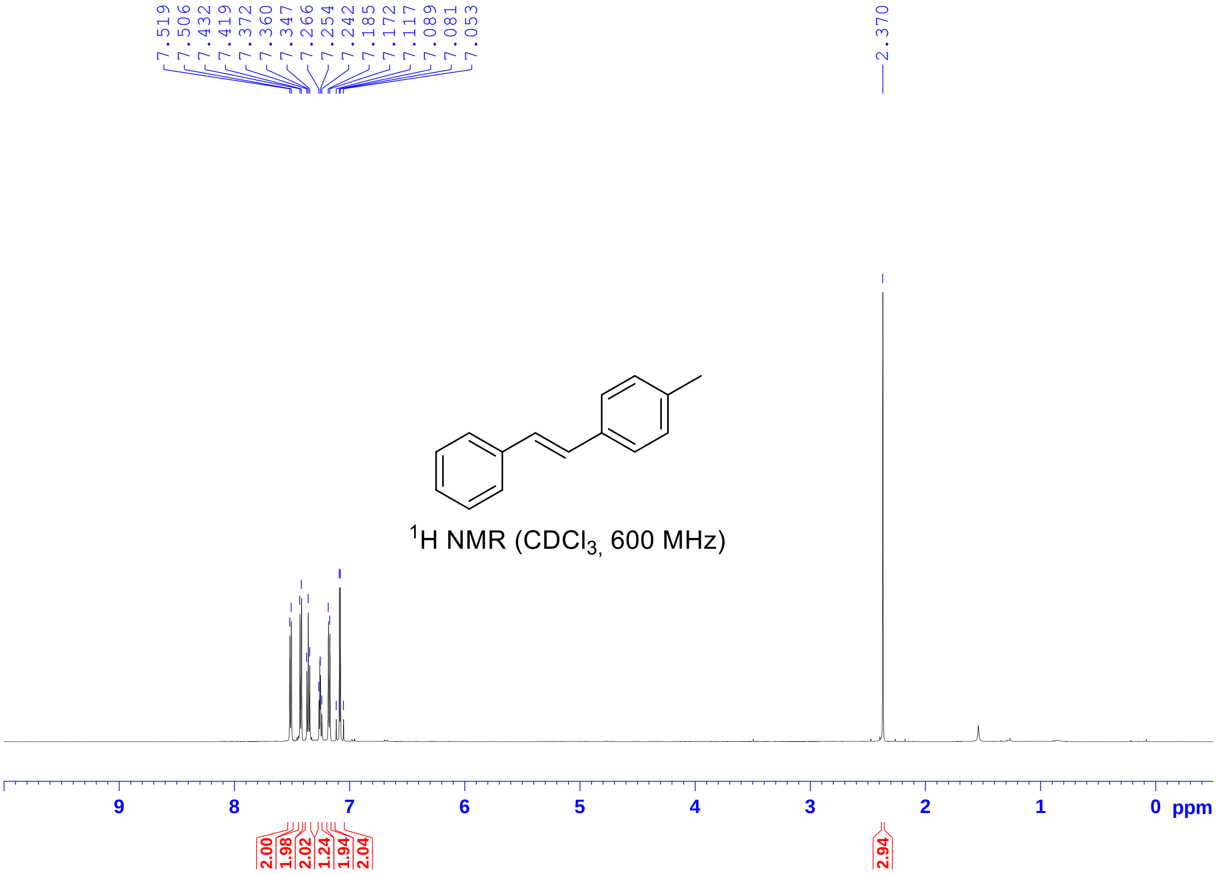

^{1}H NMR spectrum of (*E*)-1-methyl-4-styrylbenzene (**5a**) in $CDCl_3$.

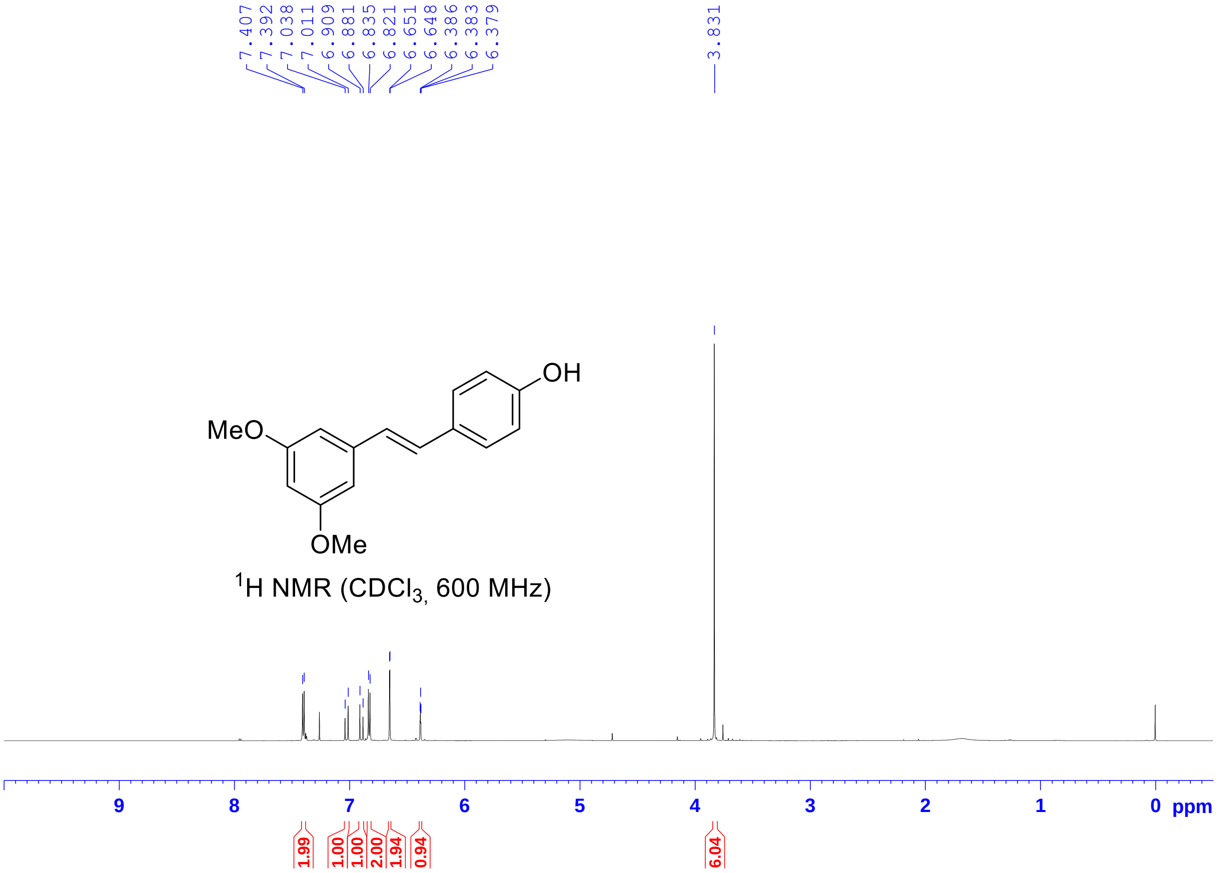

^{1}H NMR spectrum of (*E*)-1-methyl-4-styrylbenzene (pterostilbene) (**5b**) in $CDCl_3$.

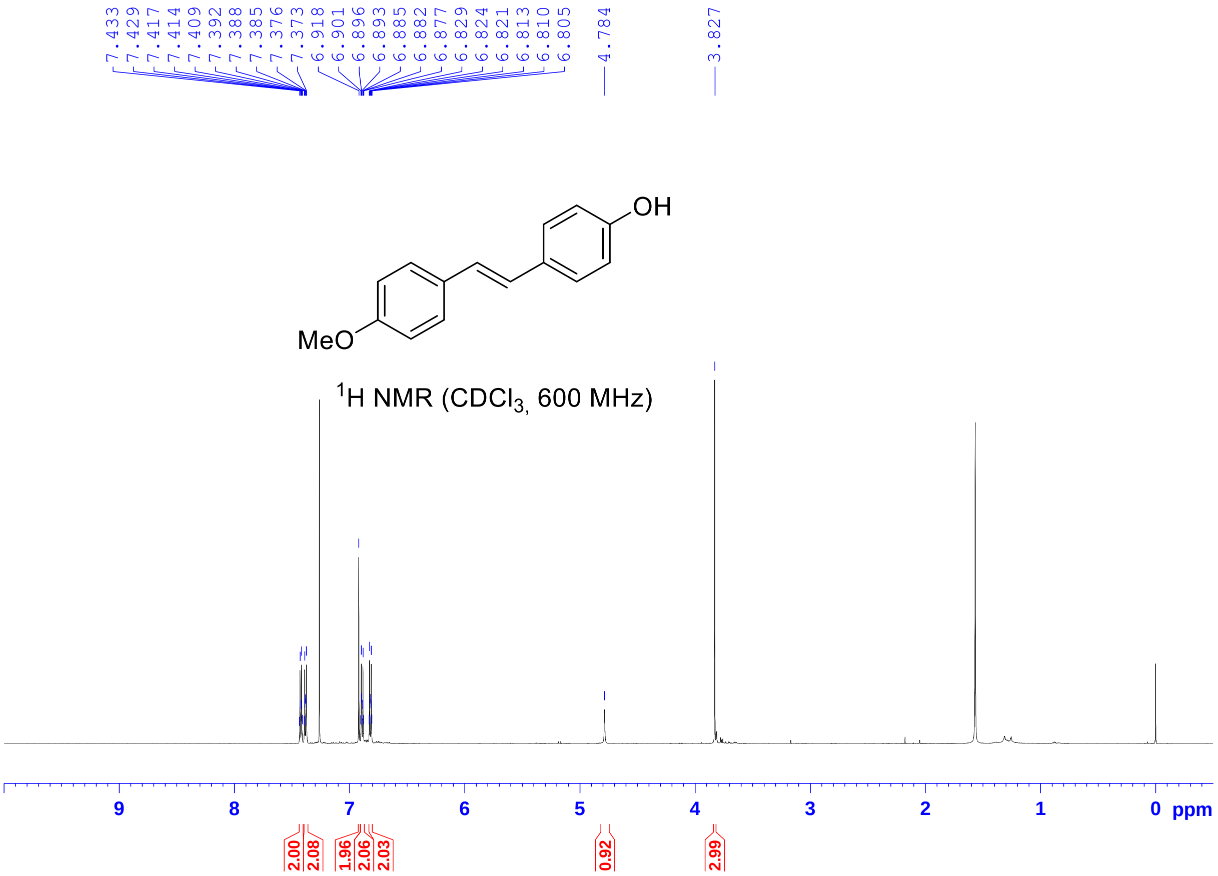

^{1}H NMR spectrum of (*E*)-4-(4-methoxystyryl)phenol (**5c**) in $CDCl_3$.

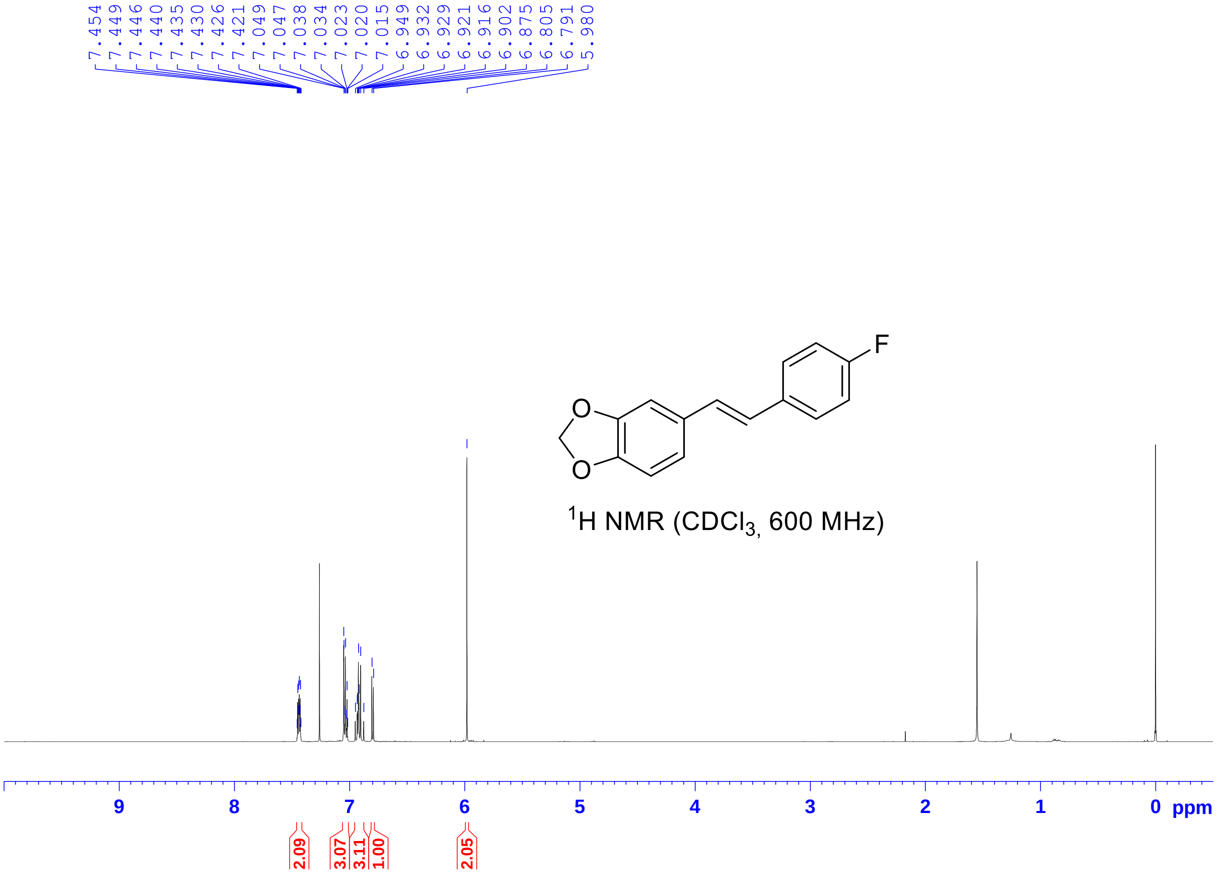

^{1}H NMR spectrum of (*E*)-5-(4-fluorostyryl)benzo[d][1,3]dioxole (**5d**) in $CDCl_3$.

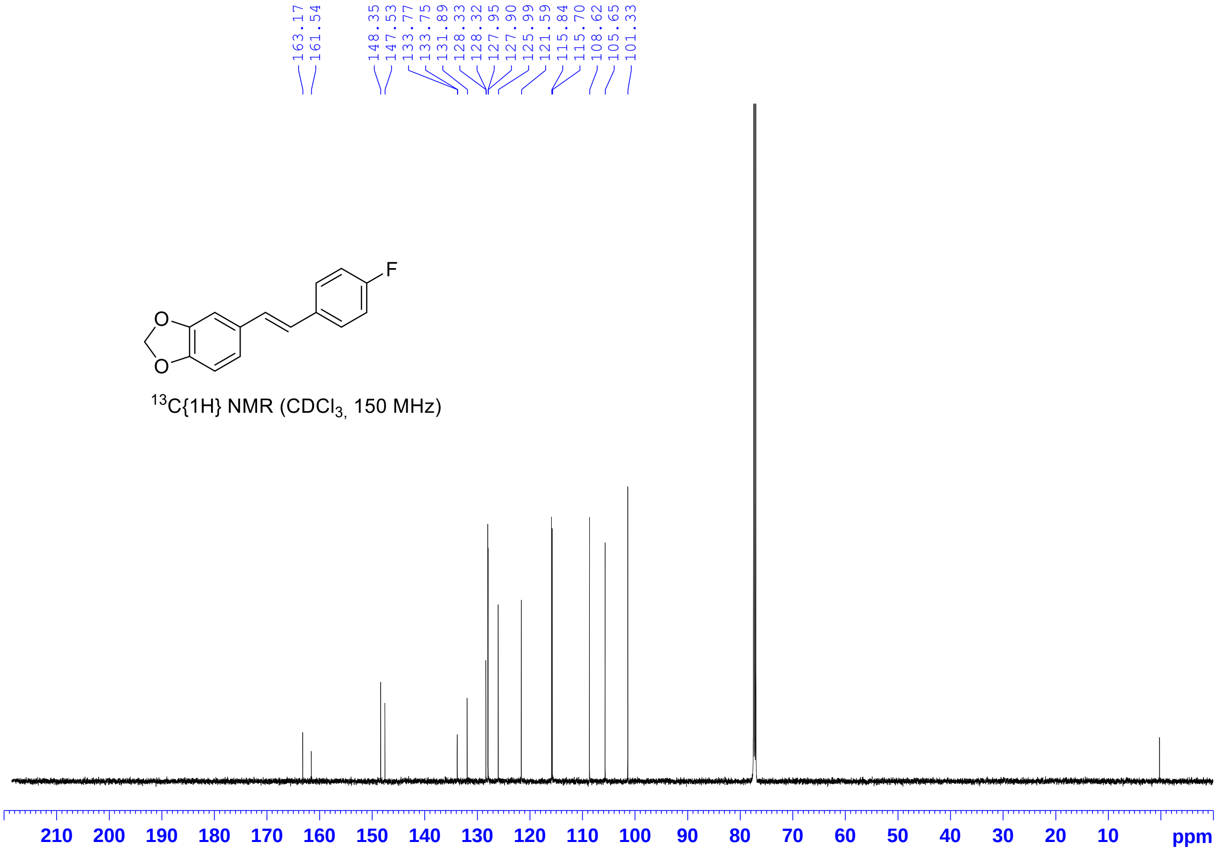

^{13}C{^{1}H} NMR spectrum of (*E*)-5-(4-fluorostyryl)benzo[d][1,3]dioxole (**5d**) in $CDCl_3$.

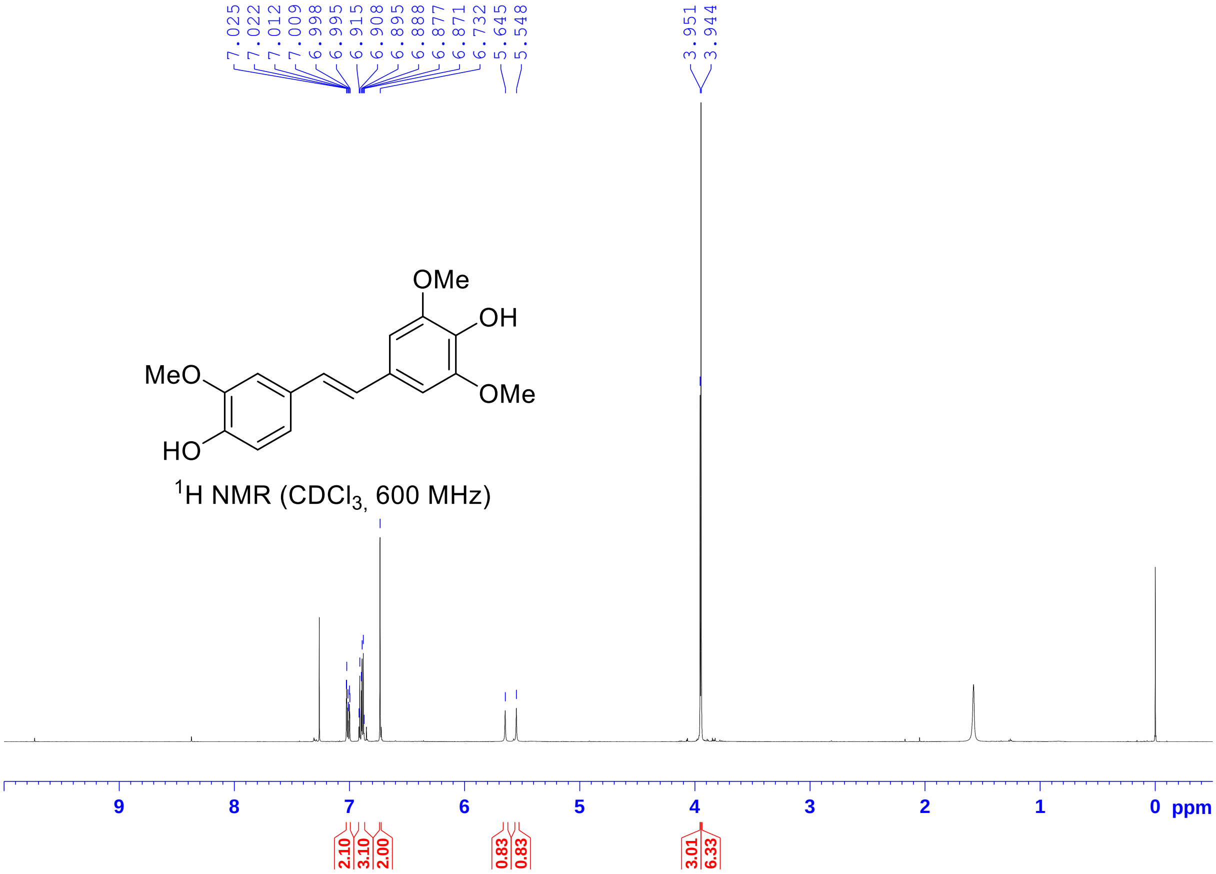

^{1}H NMR spectrum of (*E*)-4-(4-hydroxy-3-methoxystyryl)-2,6-dimethoxyphenol (**5e**) in $CDCl_3$.

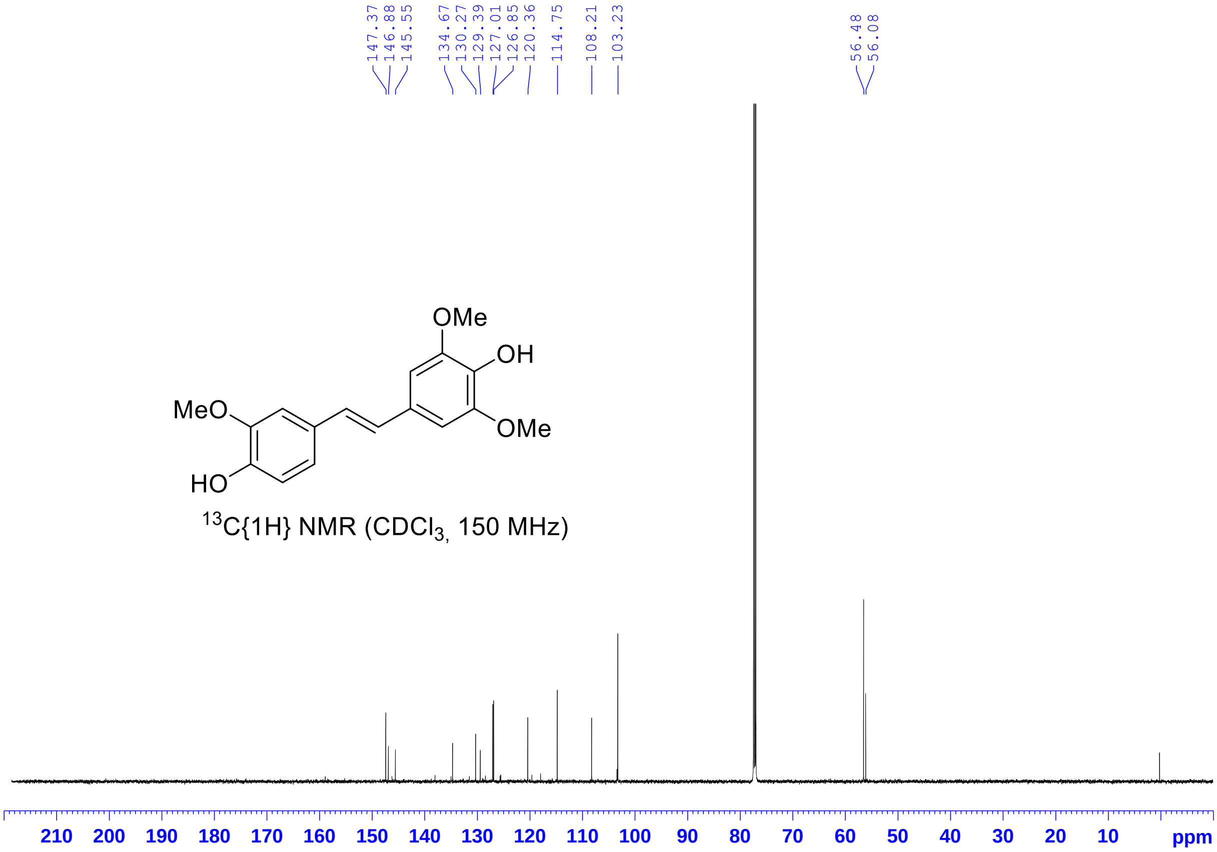

^{13}C{^{1}H} NMR spectrum of (*E*)-4-(4-hydroxy-3-methoxystyryl)-2,6-dimethoxyphenol (**5e**) in $CDCl_3$.

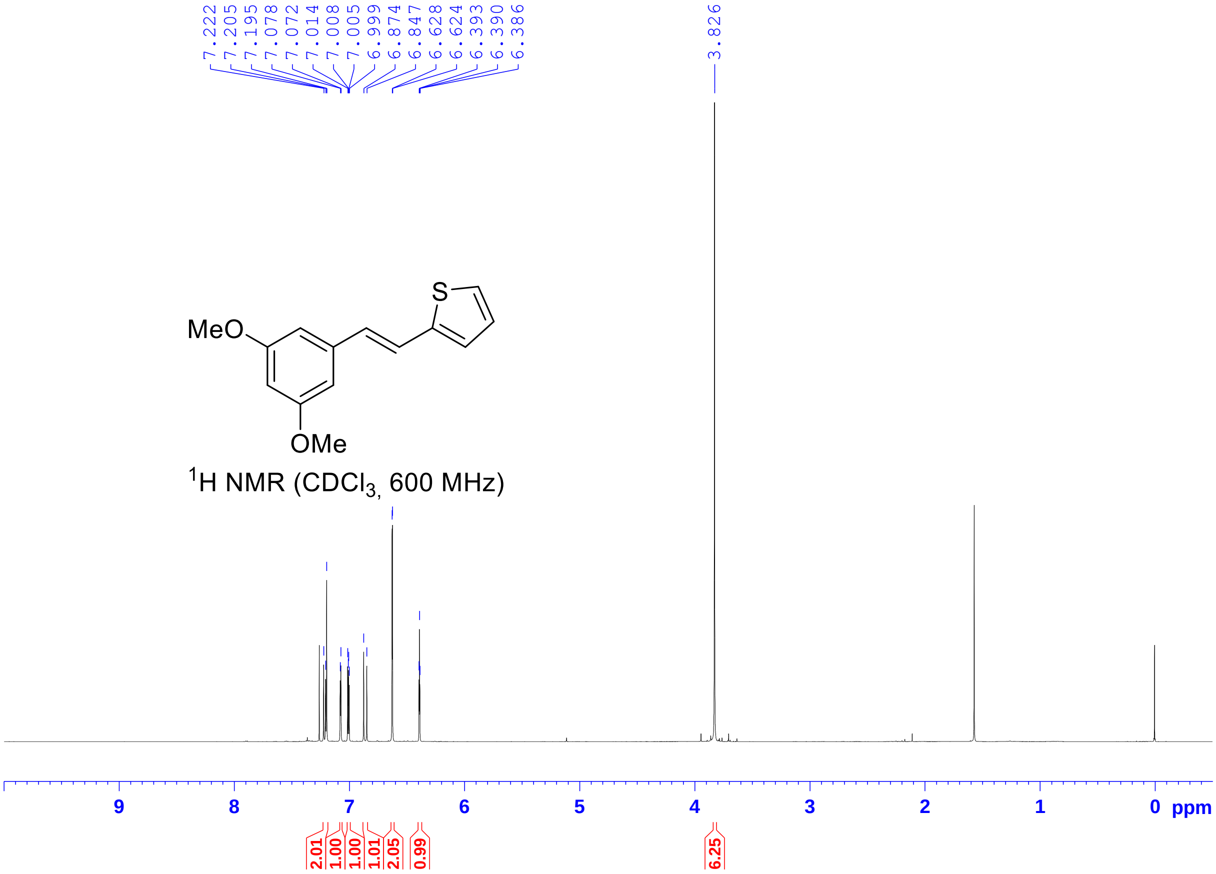

^{1}H NMR spectrum of (*E*)-2-(3,5-Dimethoxystyryl)thiophene (**5f**) in $CDCl_3$.

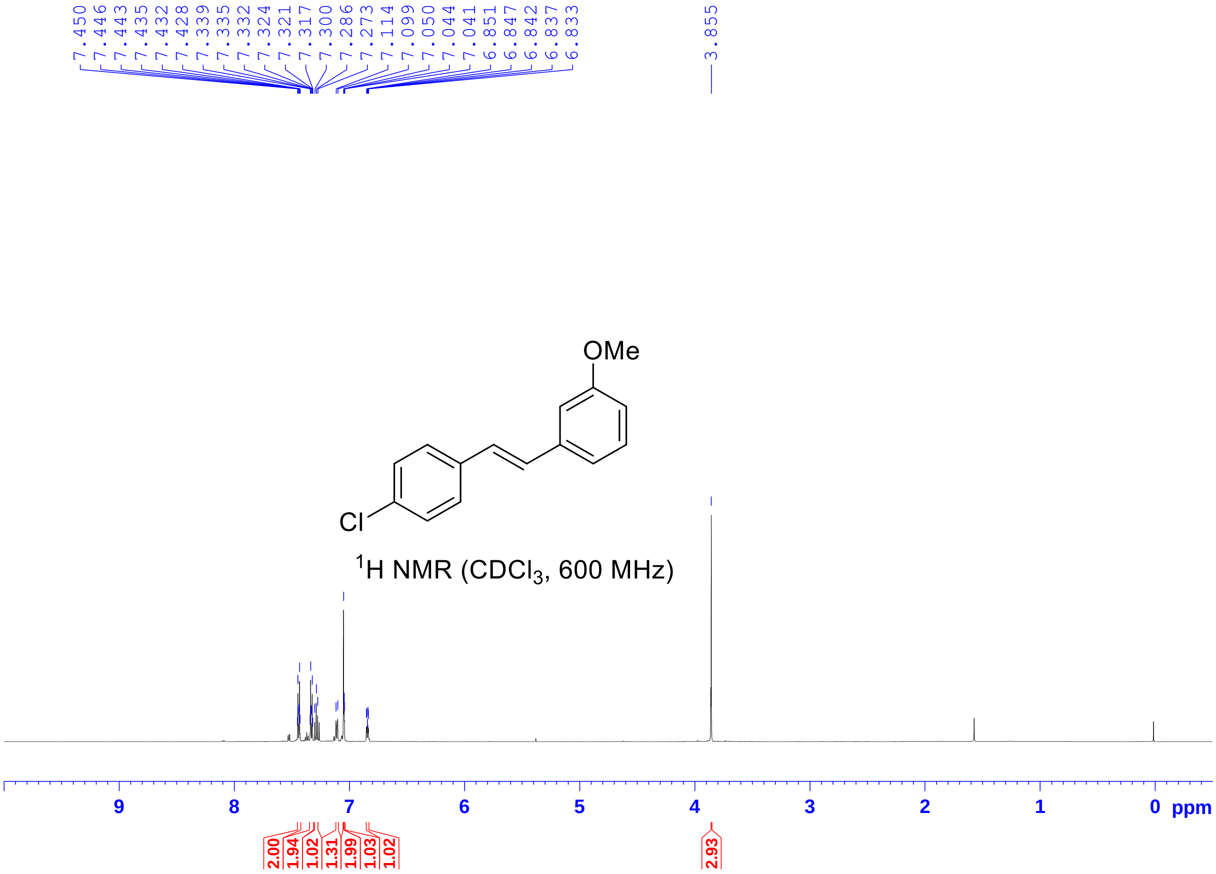

^{1}H NMR spectrum of (*E*)-1-(4-Chlorostyryl)-3-methoxybenzene (**5g**) in $CDCl_3$.

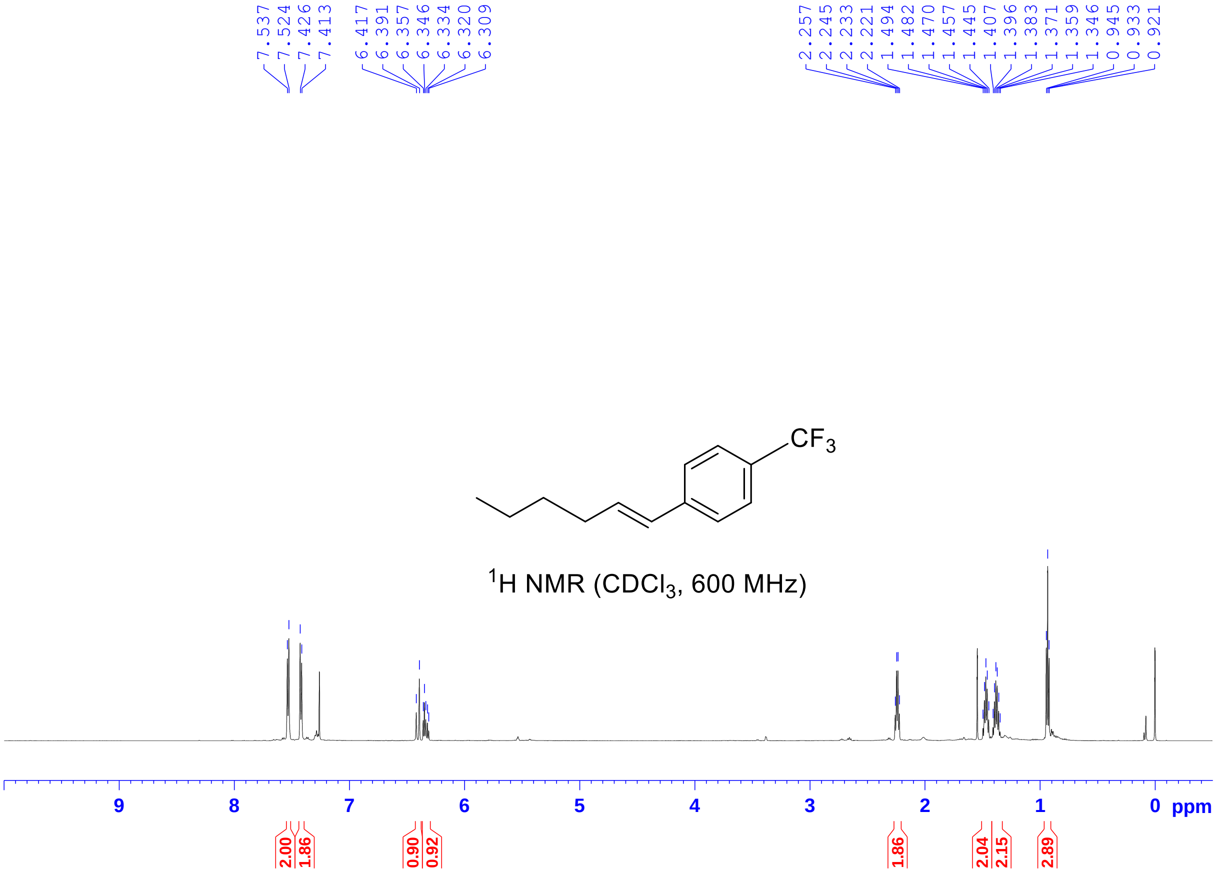

^{1}H NMR spectrum of (*E*)-1-(hex-1-en-1-yl)-4-(trifluoromethyl)benzene (**5h**) in $CDCl_3$.

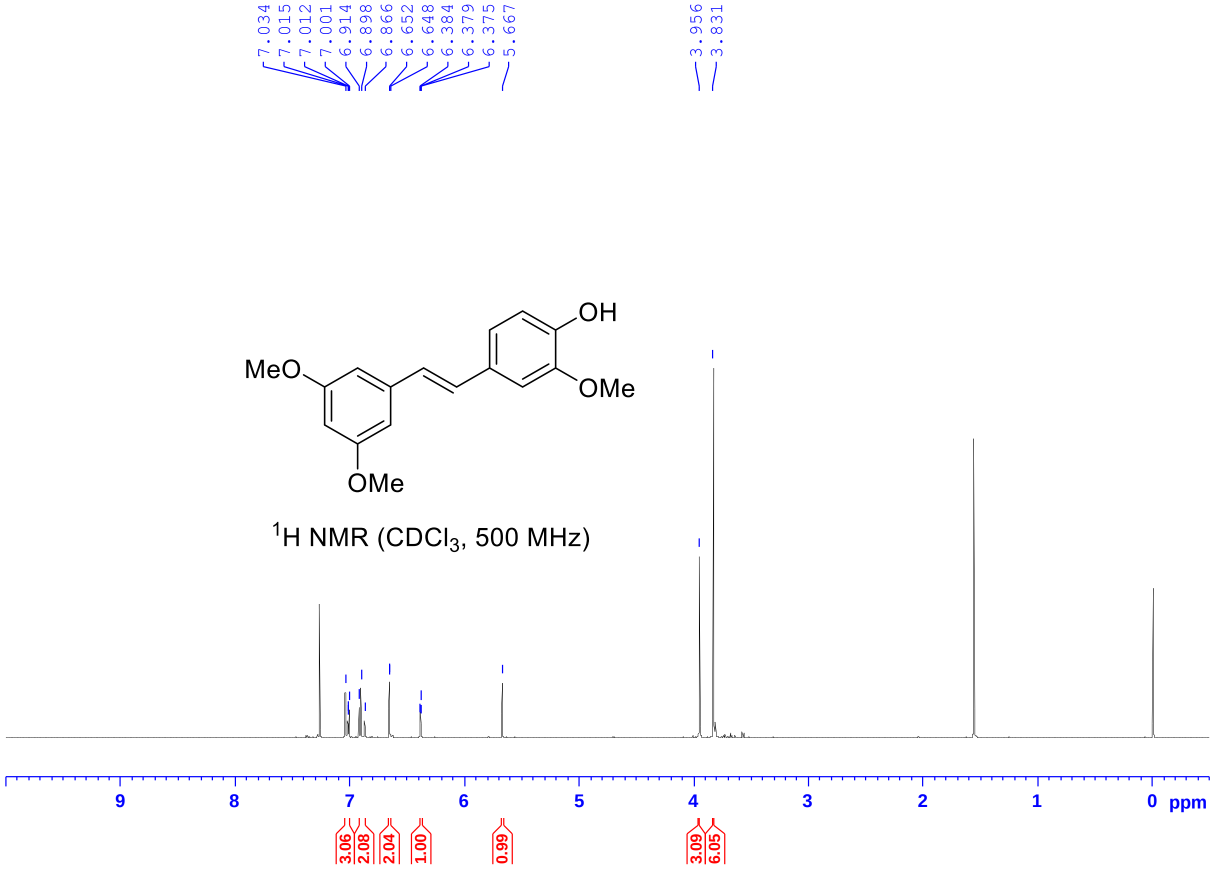

^{1}H NMR spectrum of (*E*)-4-(3,5-dimethoxystyryl)-2-methoxyphenol (isorhapontigenin) (**5i**) in $CDCl_3$.

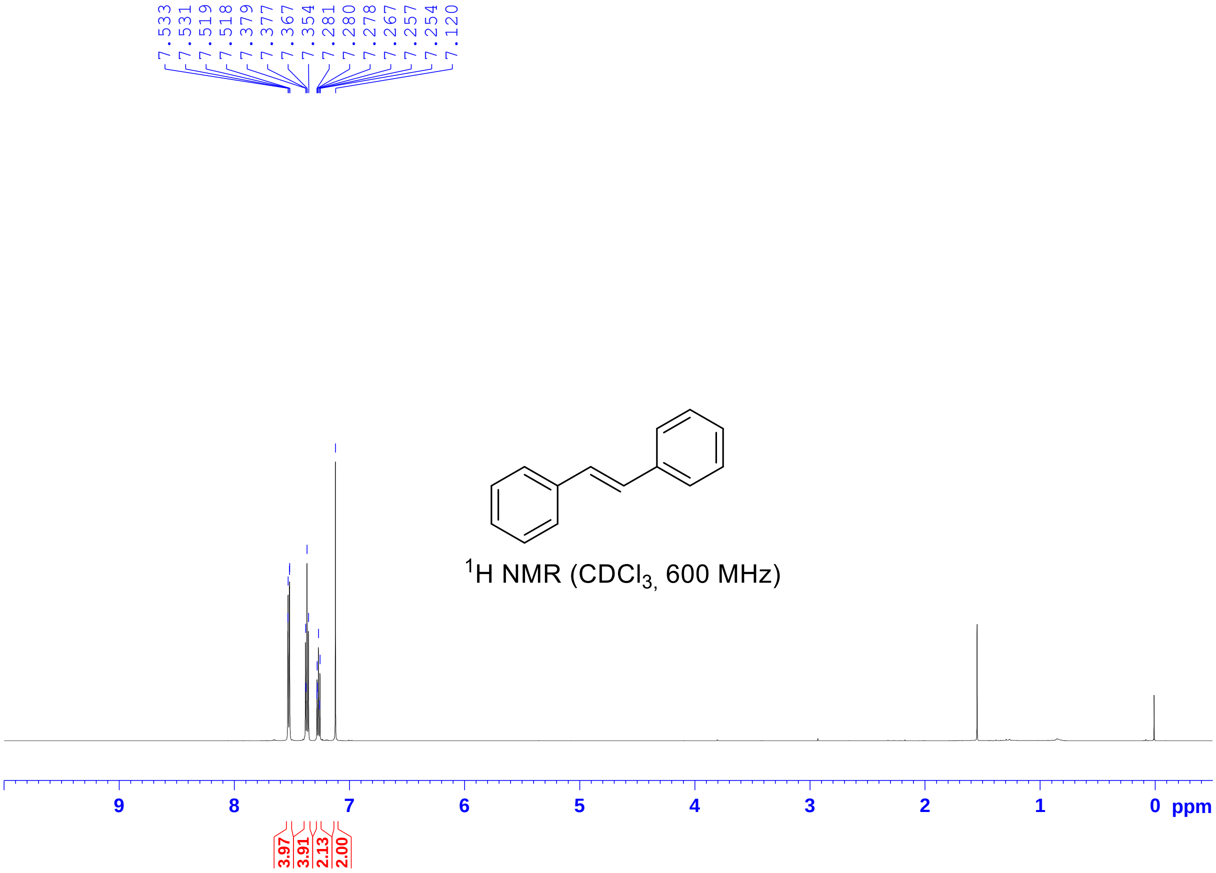

^{1}H NMR spectrum of (*E*)-1,2-diphenylethene (**5j**) in $CDCl_3$.

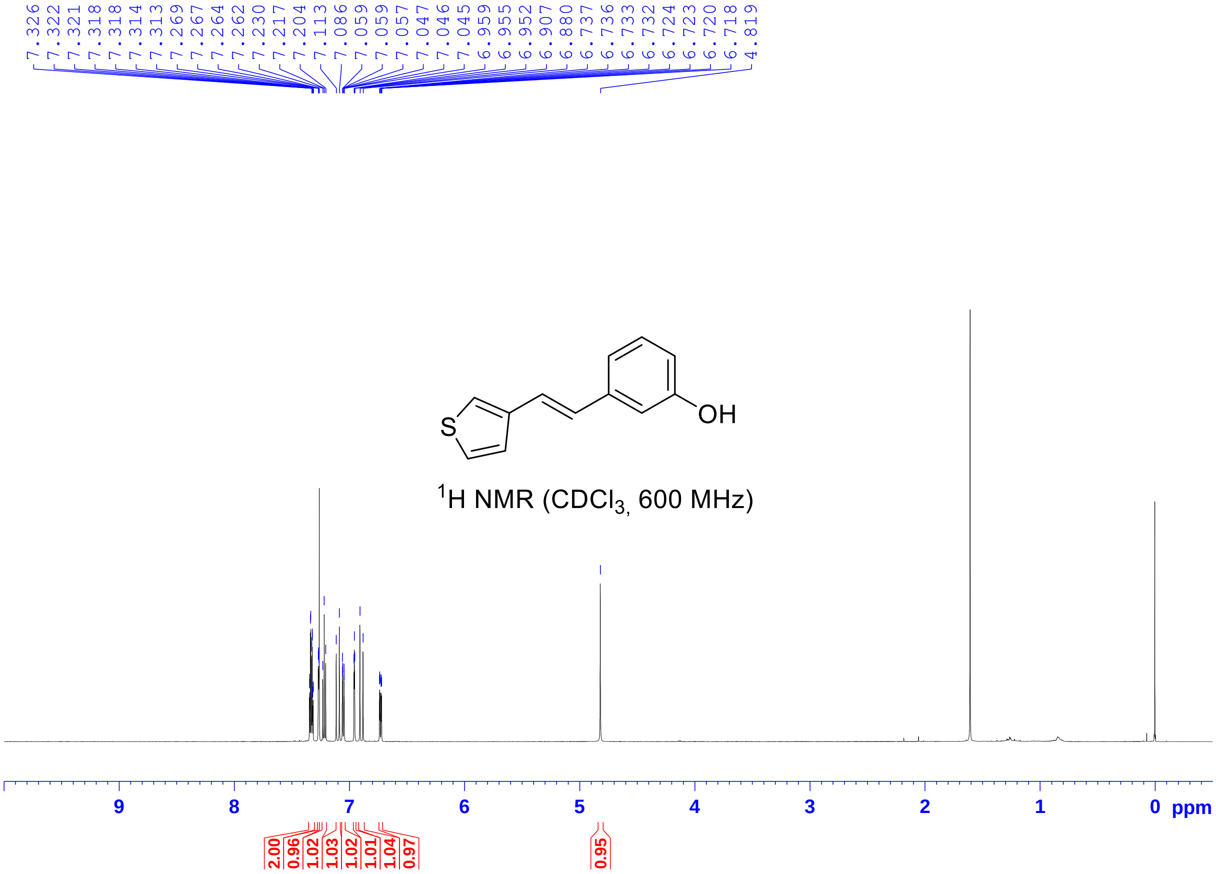

^{1}H NMR spectrum of (*E*)-3-(2-(thiophen-3-yl)vinyl)phenol (**5k**) in $CDCl_3$.

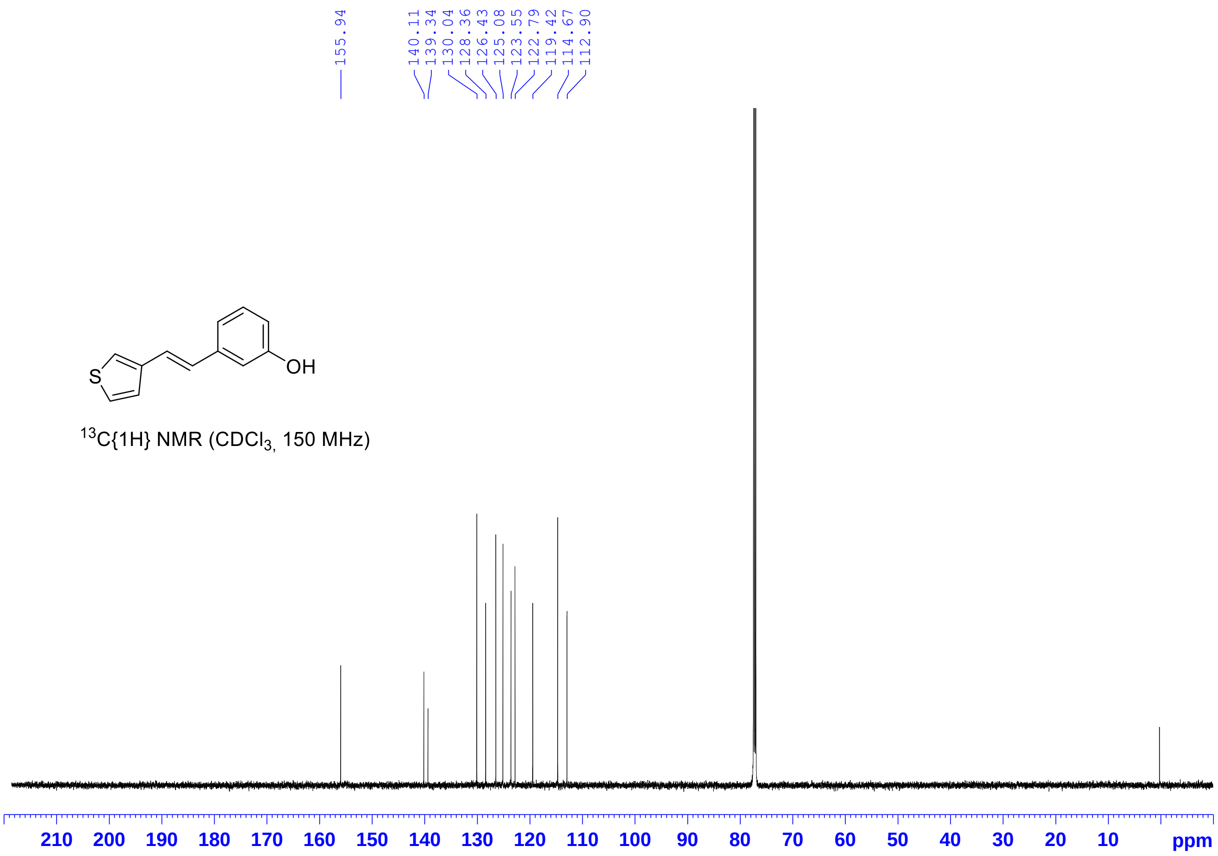

$^{13}C\{^1H\}$ NMR spectrum of (*E*)-3-(2-(thiophen-3-yl)vinyl)phenol (**5k**) in $CDCl_3$.

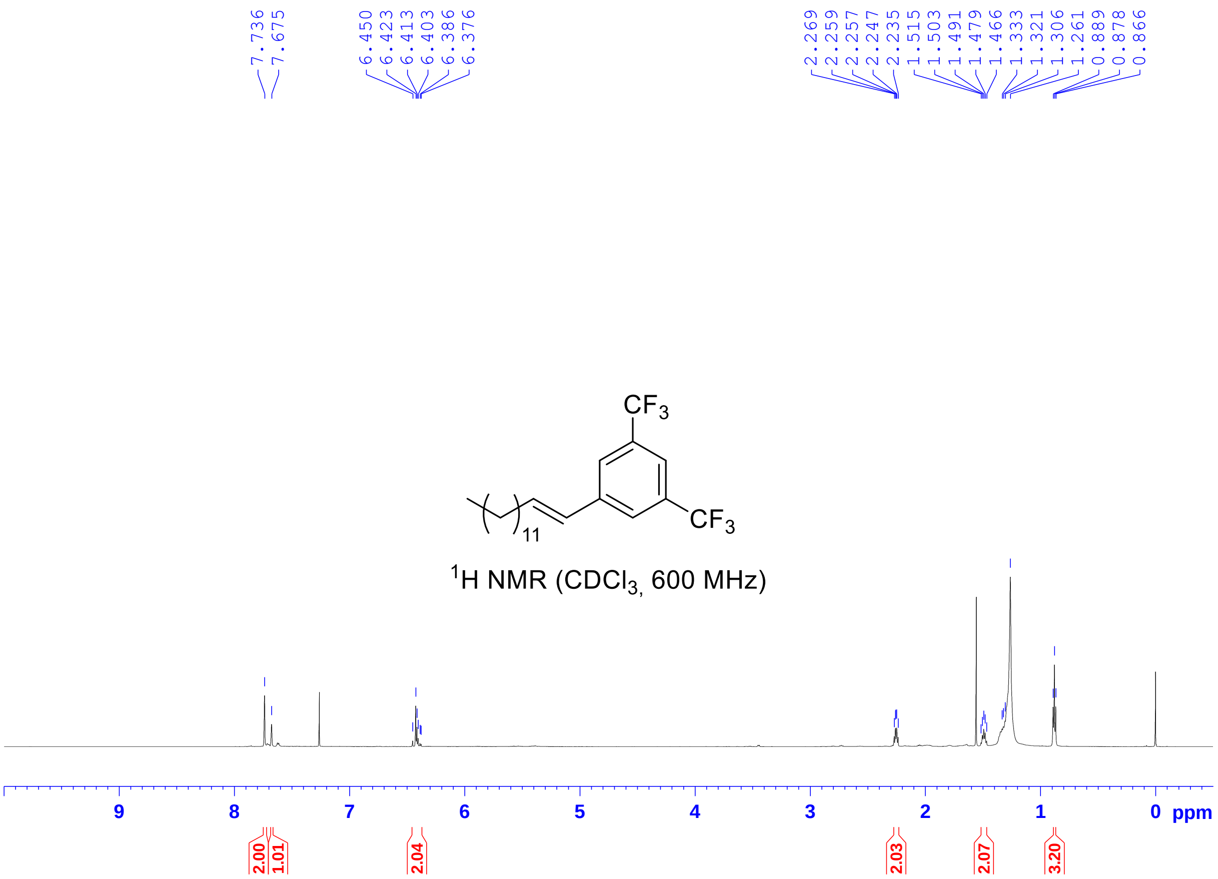

^1H NMR spectrum of (*E*)-1-(tetradec-1-en-1-yl)-3,5-bis(trifluoromethyl)benzene (**5l**) in $CDCl_3$.

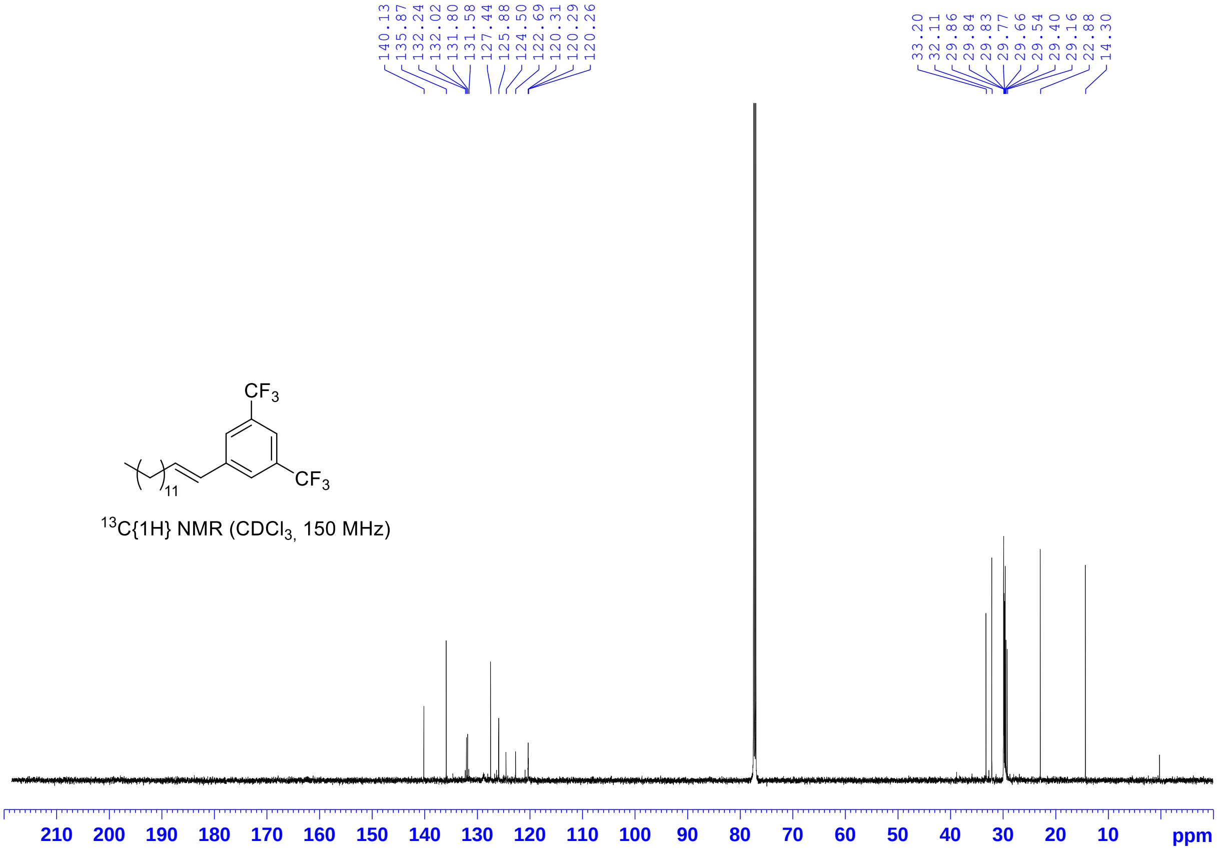

$^{13}C\{^1H\}$ NMR spectrum of (*E*)-1-(tetradec-1-en-1-yl)-3,5-bis(trifluoromethyl)benzene (**5l**) in $CDCl_3$.

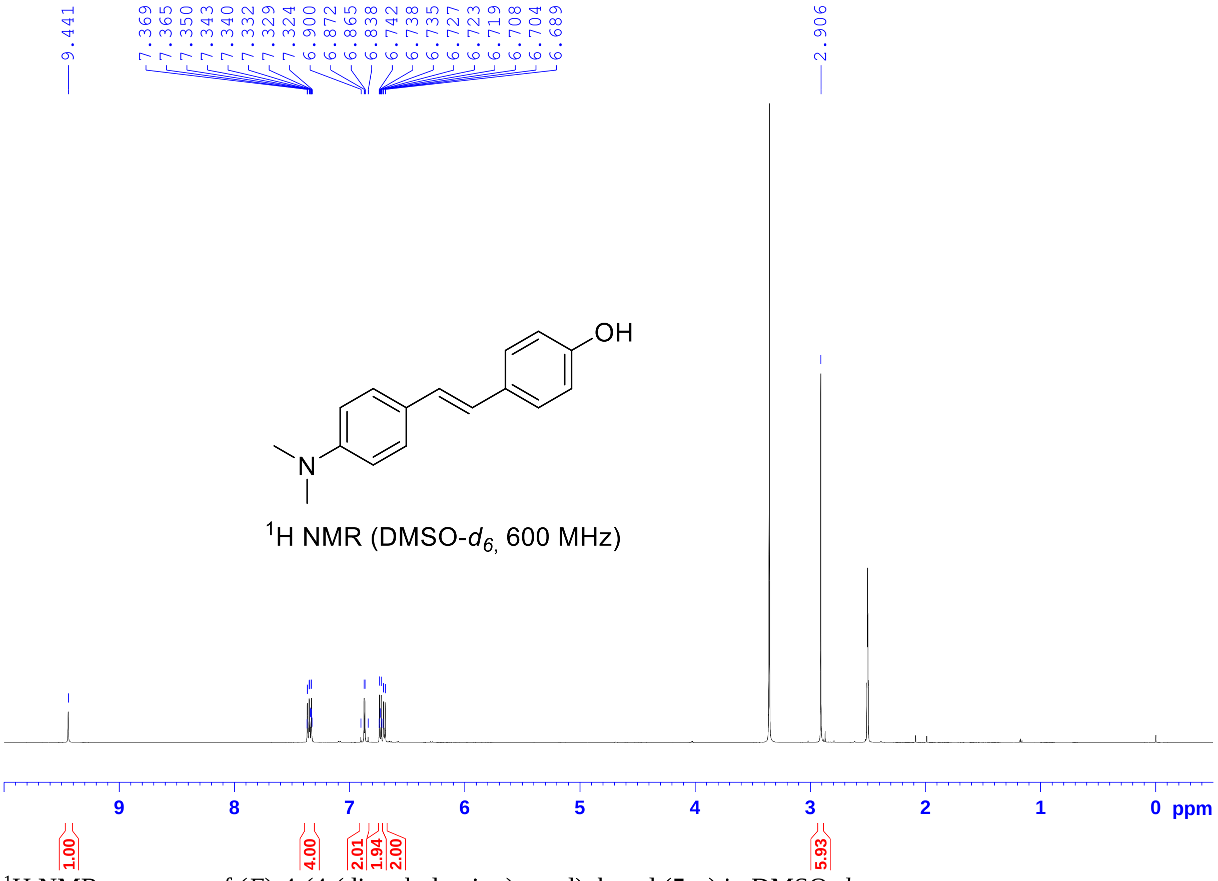

^1H NMR spectrum of (*E*)-4-(4-(dimethylamino)styryl)phenol (**5m**) in DMSO-d_6.

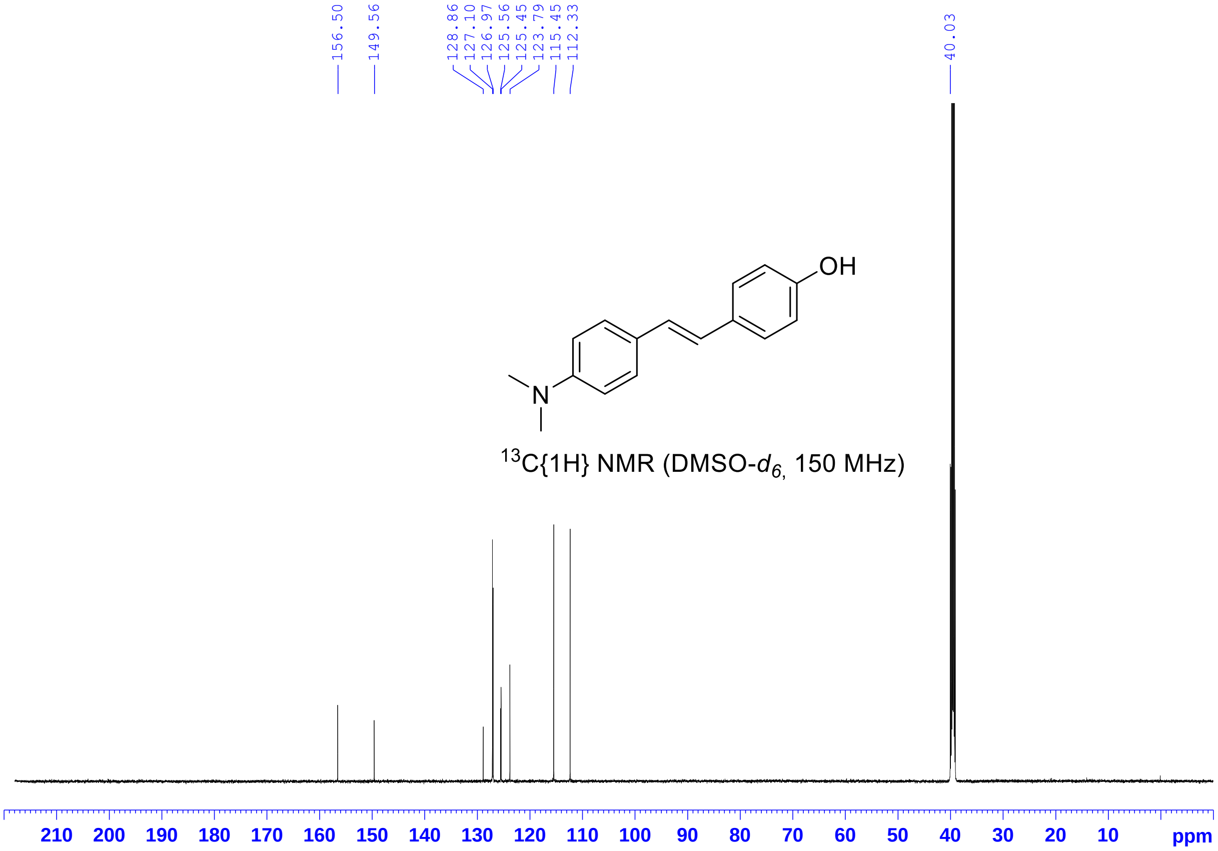

$^{13}C\{^{1}H\}$ NMR spectrum of (*E*)-4-(4-(dimethylamino)styryl)phenol (**5m**) in DMSO-d_6.

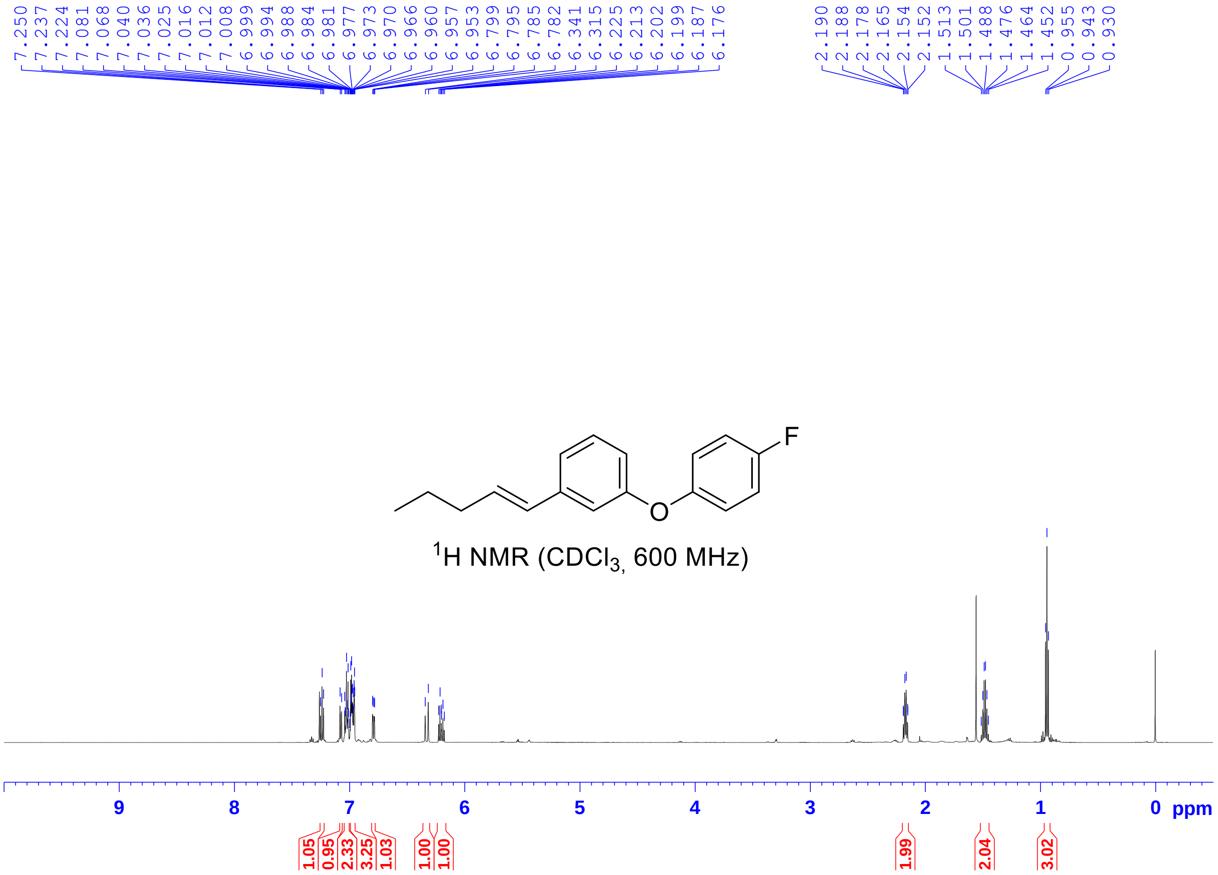

^{1}H NMR spectrum of (*E*)-1-(4-fluorophenoxy)-3-(pent-1-en-1-yl)benzene (**5n**) in $CDCl_3$.

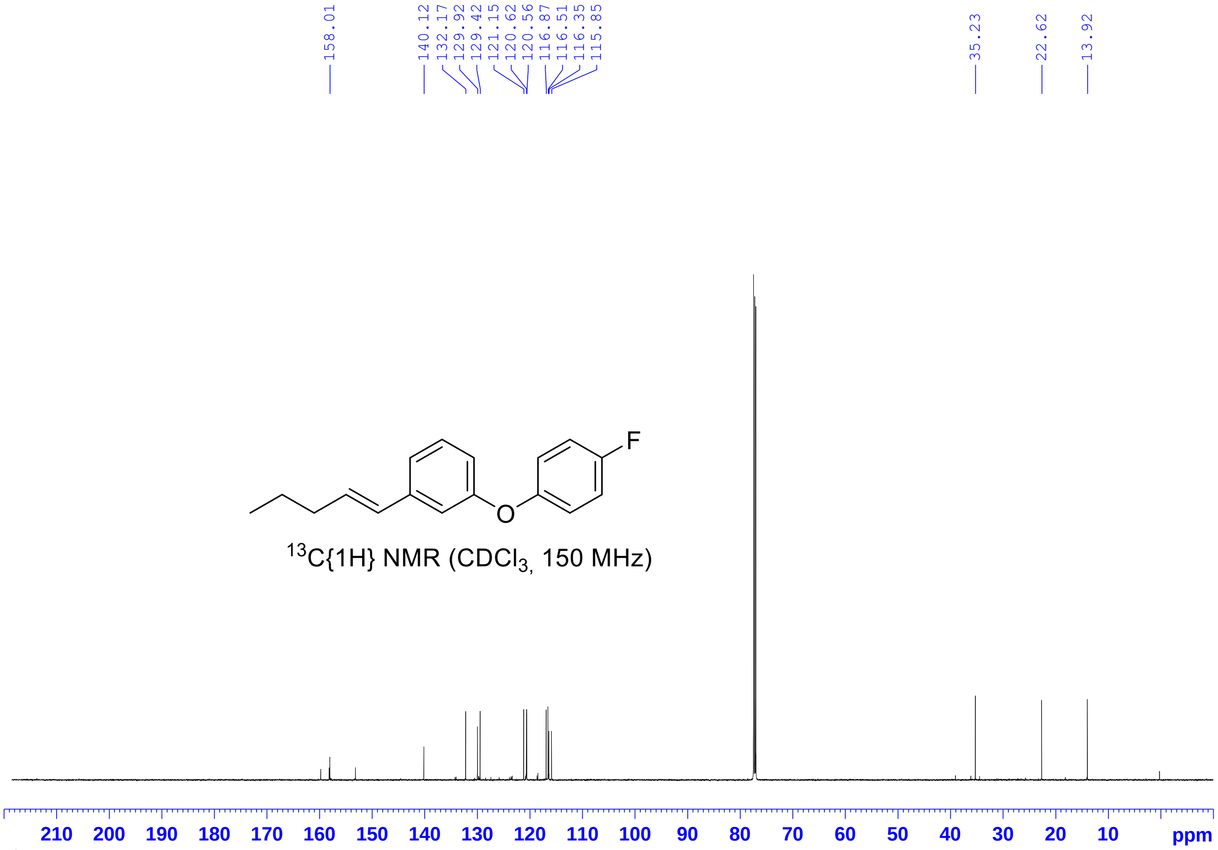

^{13}C{^{1}H} NMR spectrum of (*E*)-1-(4-fluorophenoxy)-3-(pent-1-en-1-yl)benzene (**5n**) in $CDCl_3$.

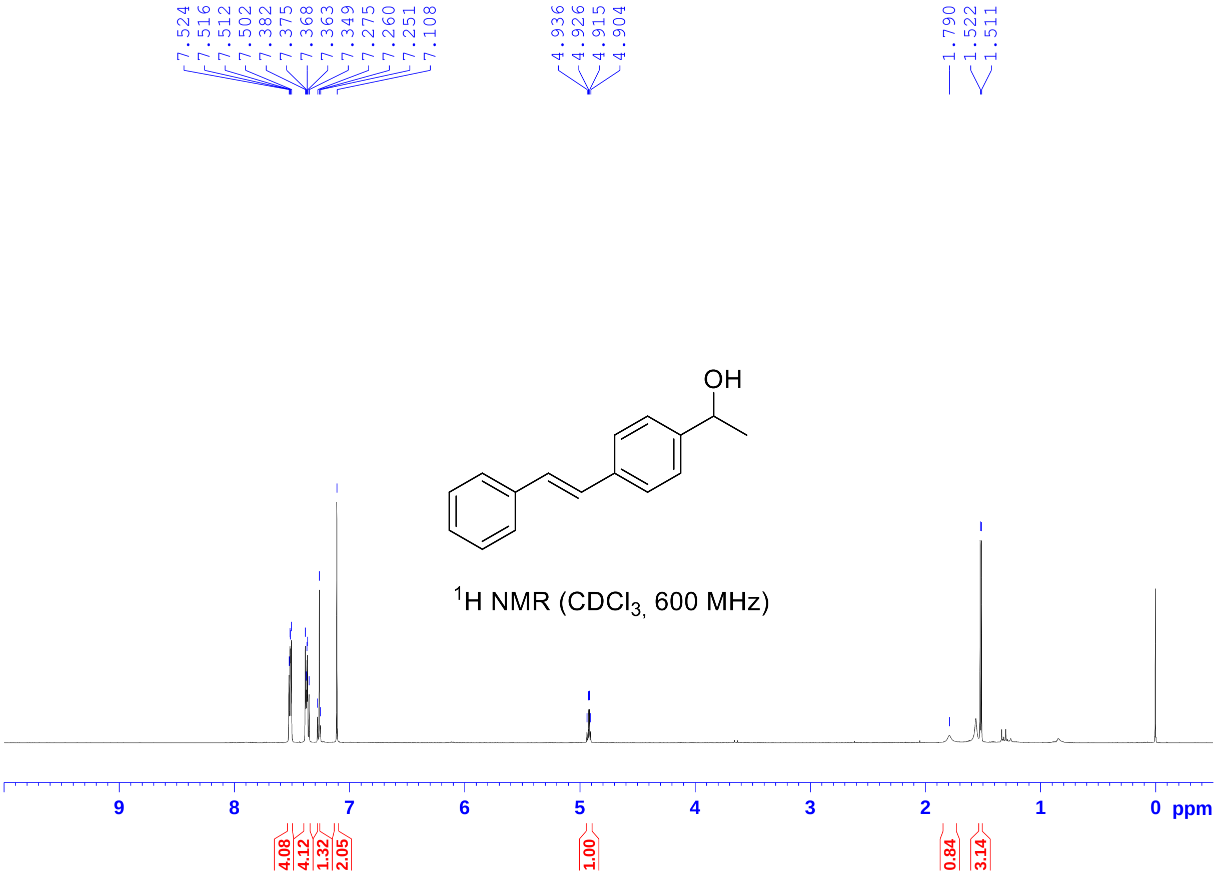

^{1}H NMR spectrum of (*E*)-1-(4-styrylphenyl)ethan-1-ol (**5o**) in $CDCl_3$.

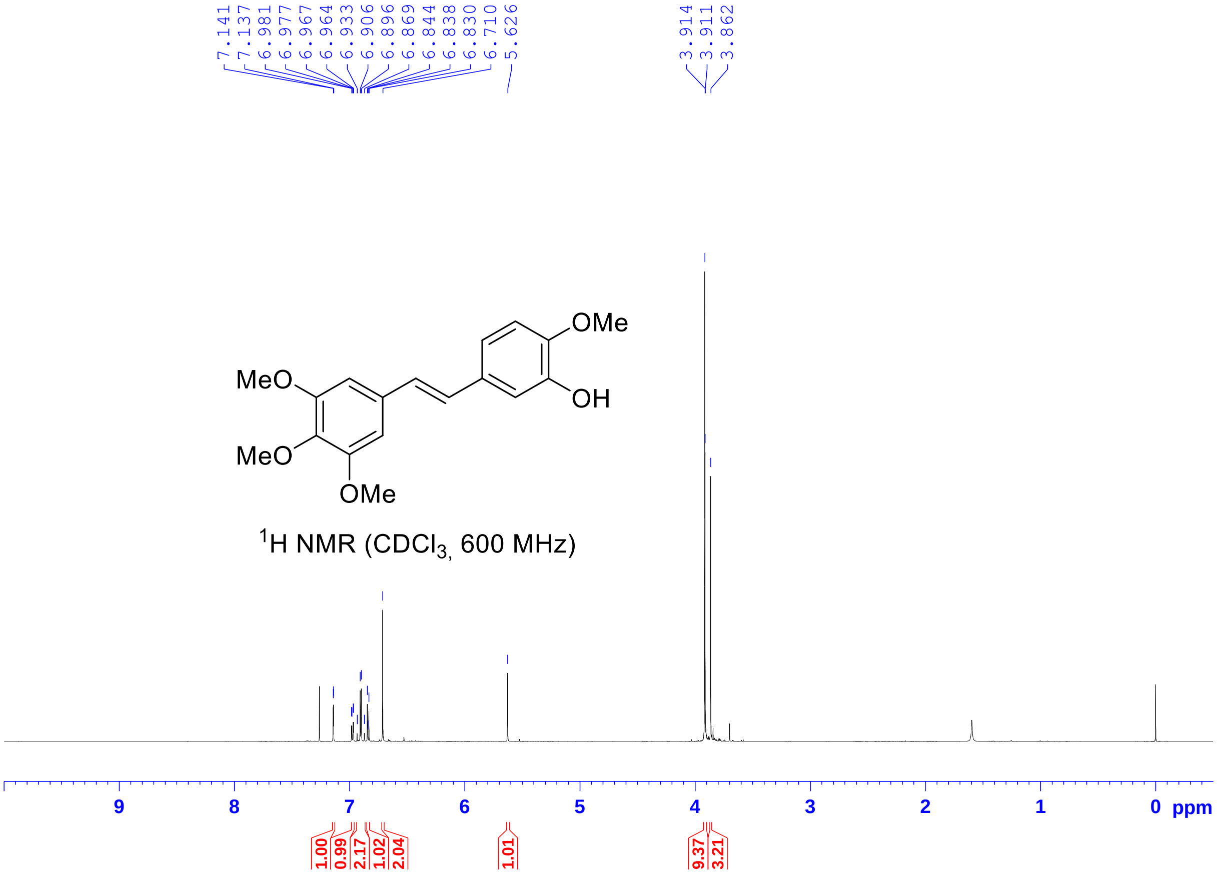

^{1}H NMR spectrum of (*E*)-2-Methoxy-5-(3,4,5-trimethoxystyryl)phenol ("*trans*-combretastatin") (**5p**) in $CDCl_3$.

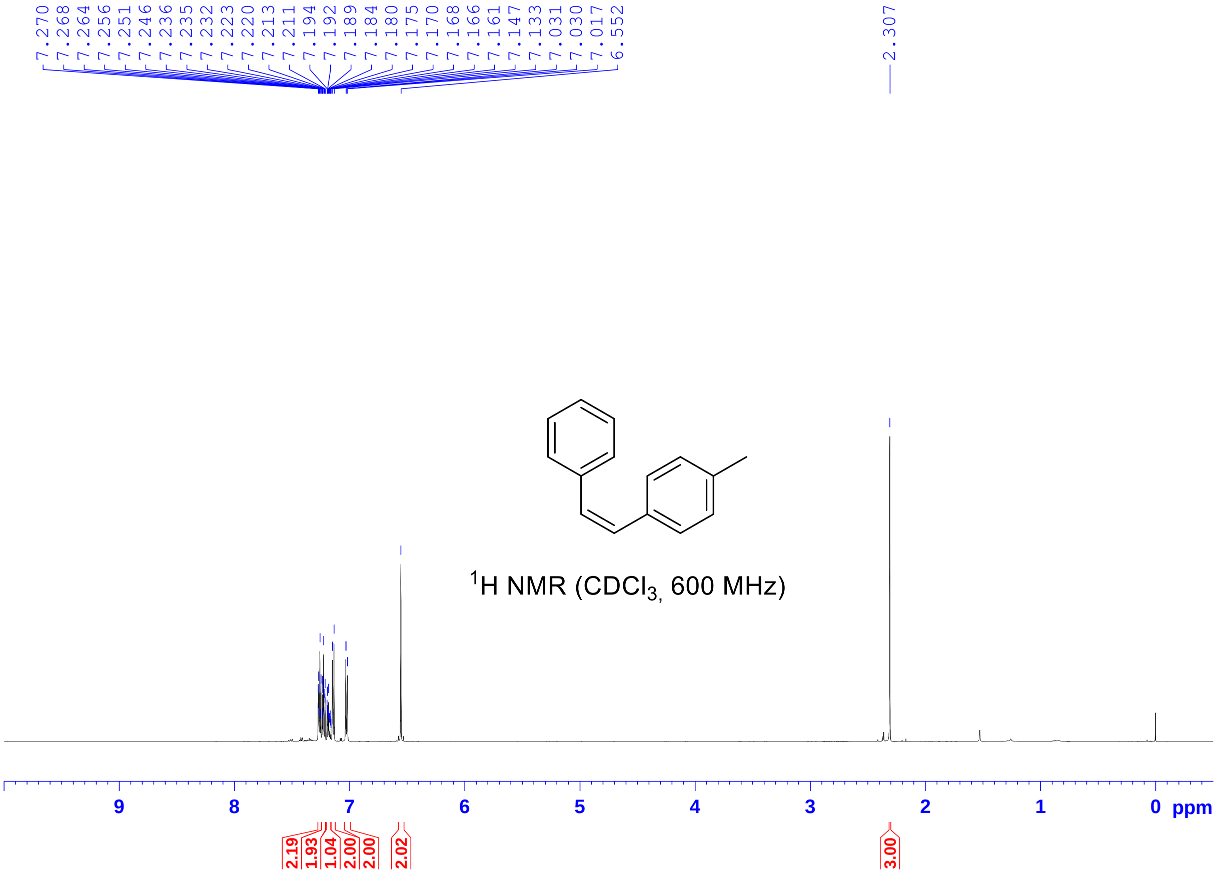

^{1}H NMR spectrum of (*Z*)-1-Methyl-4-styrylbenzene (**5r**) in $CDCl_3$.

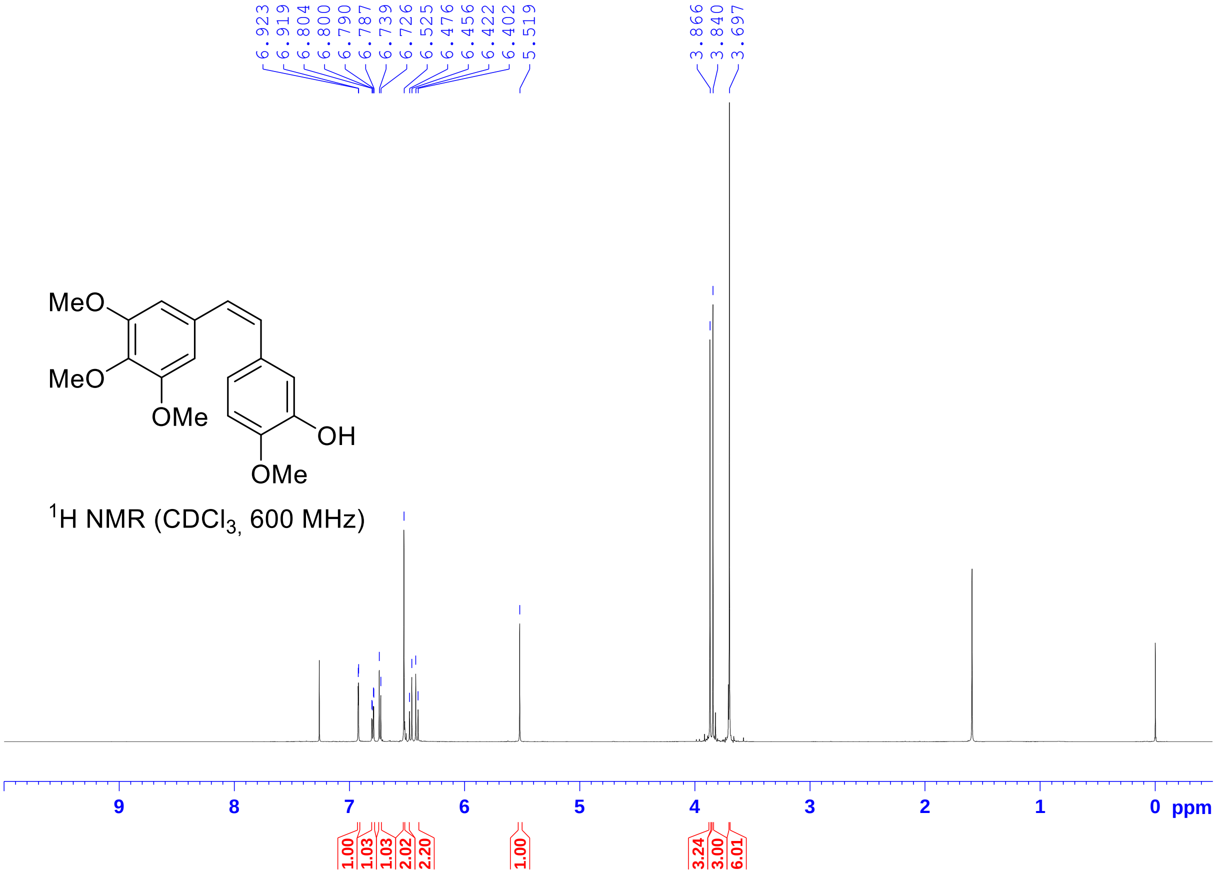

^{1}H NMR spectrum of (*Z*)-2-Methoxy-5-(3,4,5-trimethoxystyryl)phenol (combretastatin A4) (**5s**) in $CDCl_3$.

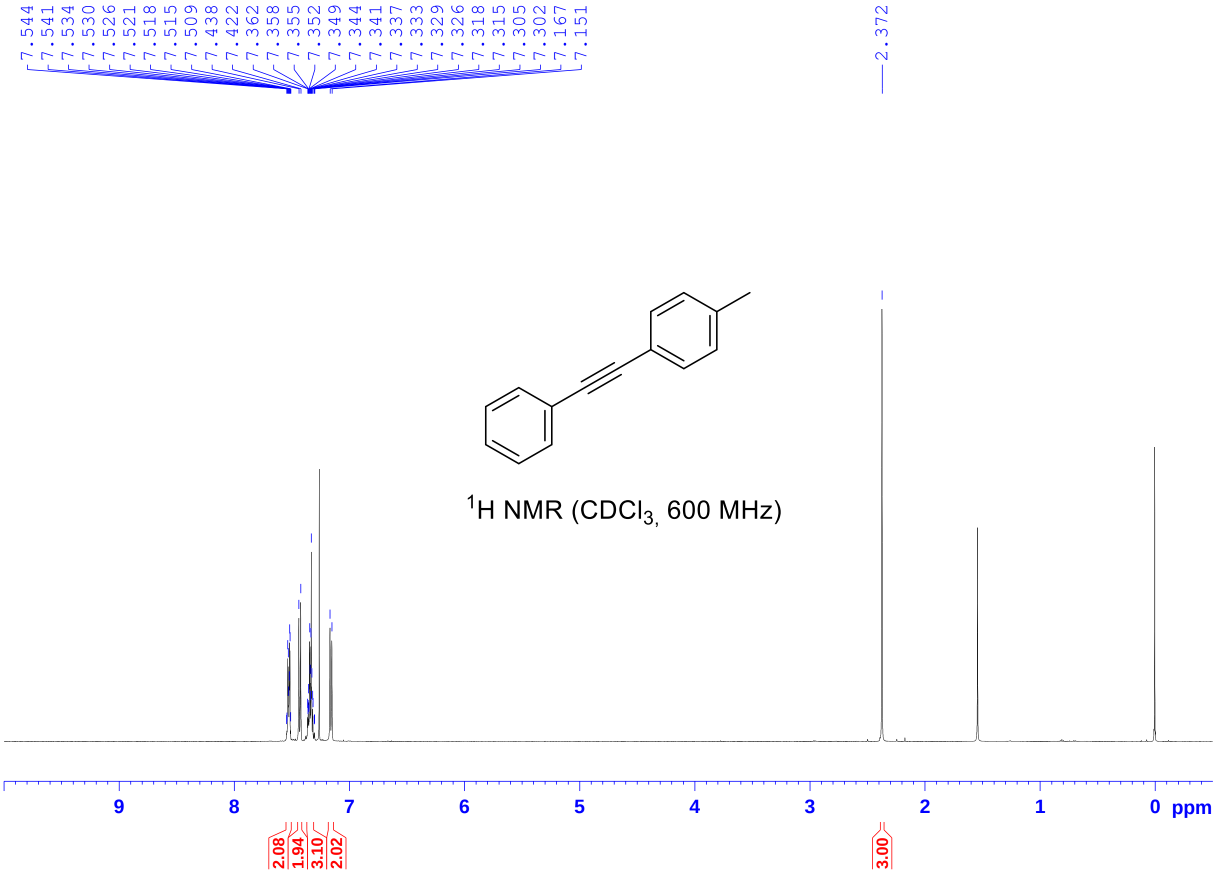

^{1}H NMR spectrum of 1-methyl-4-(phenylethynyl)benzene (**1b**) in $CDCl_3$.

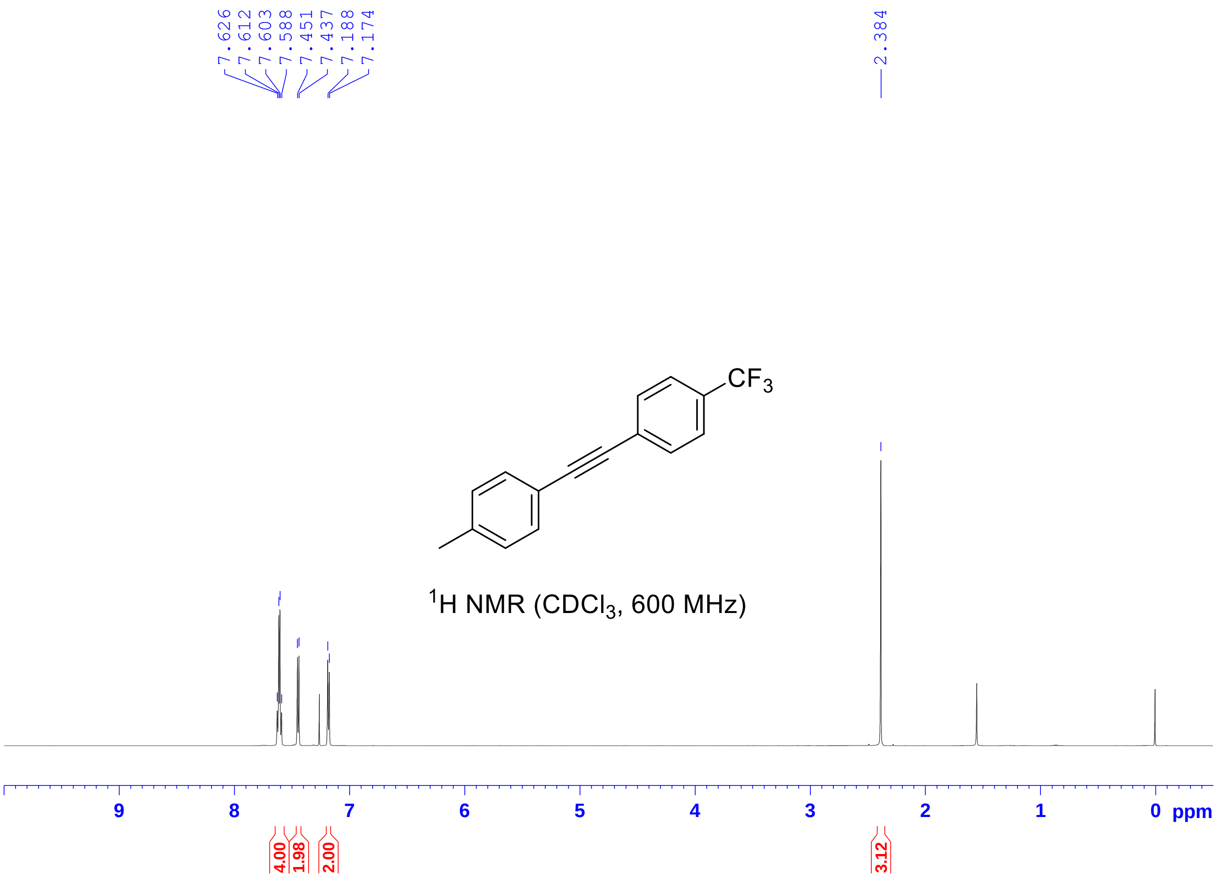

^{1}H NMR spectrum of 1-methyl-4-((4-(trifluoromethyl)phenyl)ethynyl)benzene (**1c**) in $CDCl_3$.

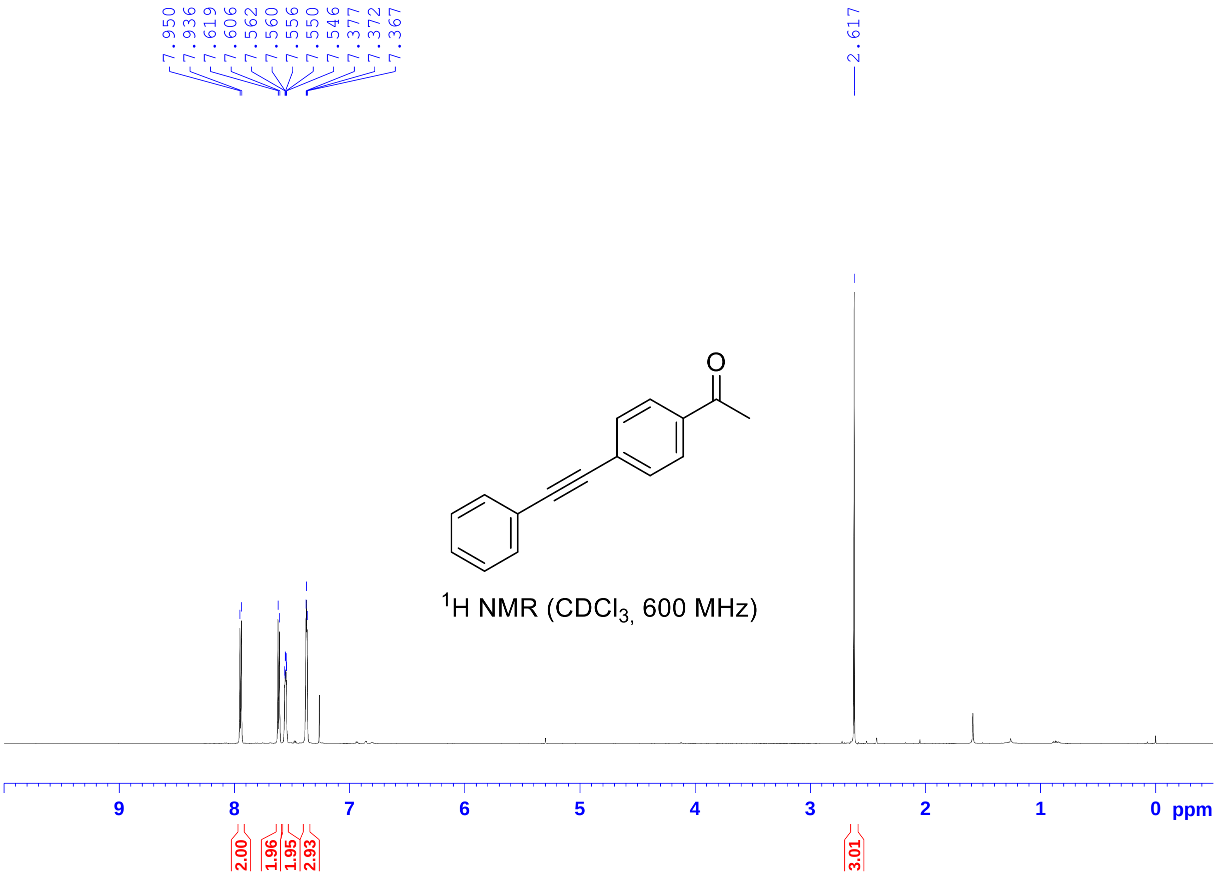

^{1}H NMR spectrum of (1-(4-(phenylethynyl)phenyl)ethan-1-one (**1d**) in $CDCl_3$.

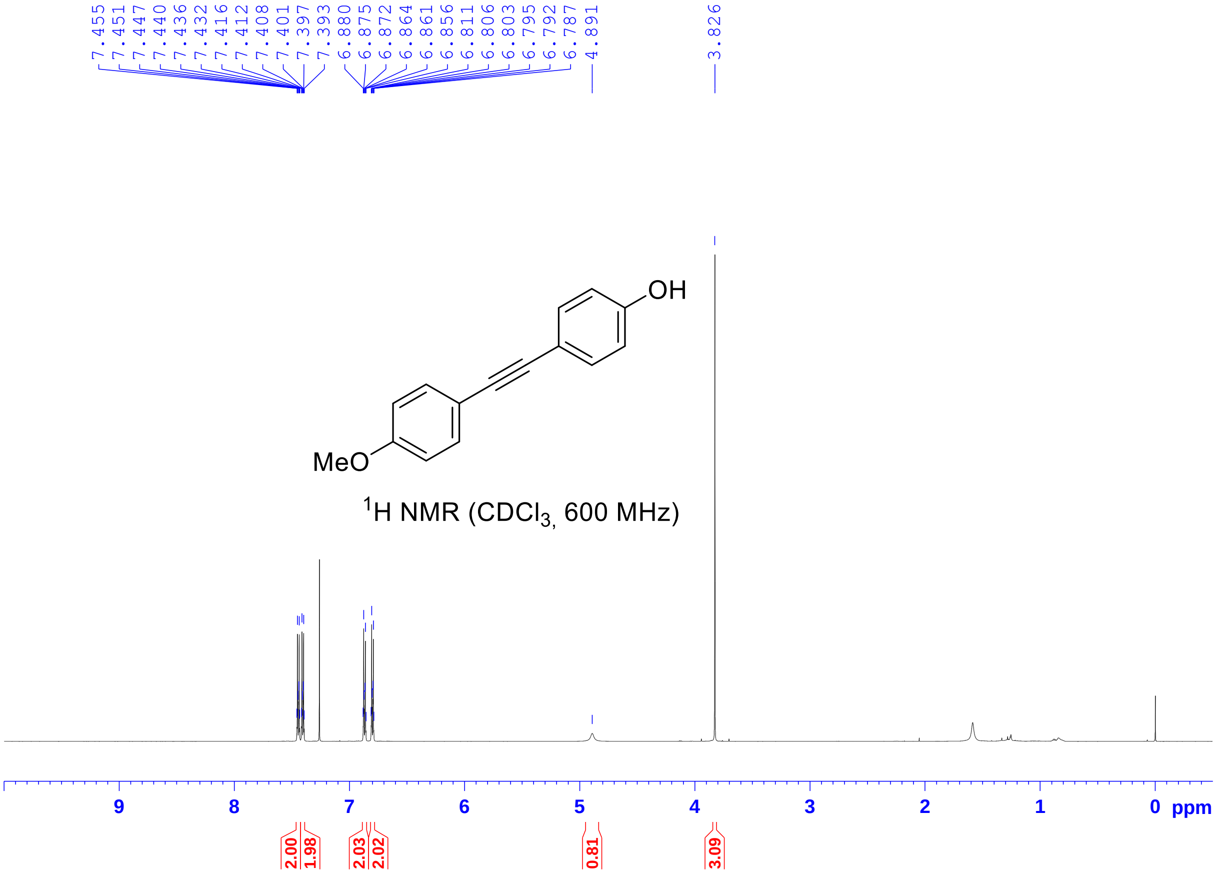

^{1}H NMR spectrum of 4-((4-methoxyphenyl)ethynyl)phenol (**1e**) in $CDCl_3$.

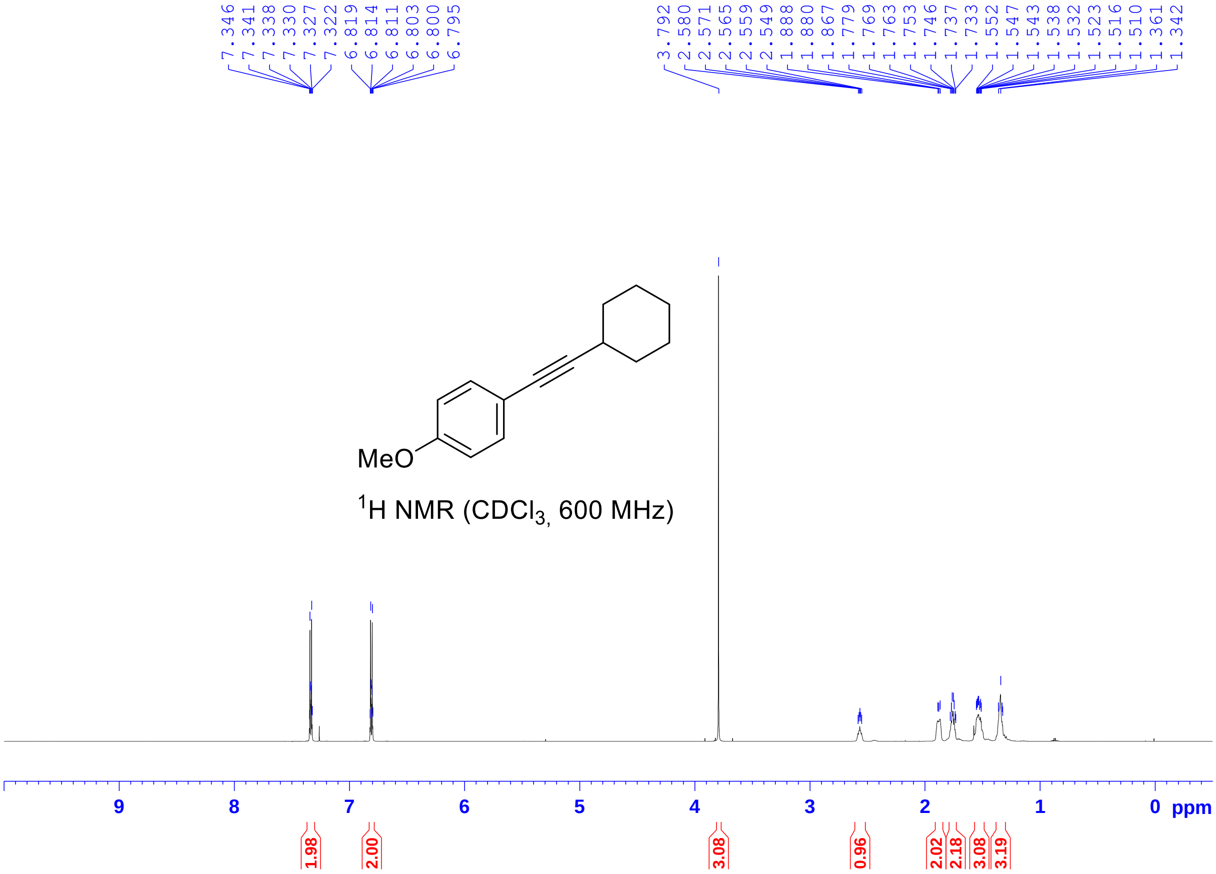

^{1}H NMR spectrum of 1-(cyclohexylethynyl)-4-methoxybenzene (**1f**) in $CDCl_3$.

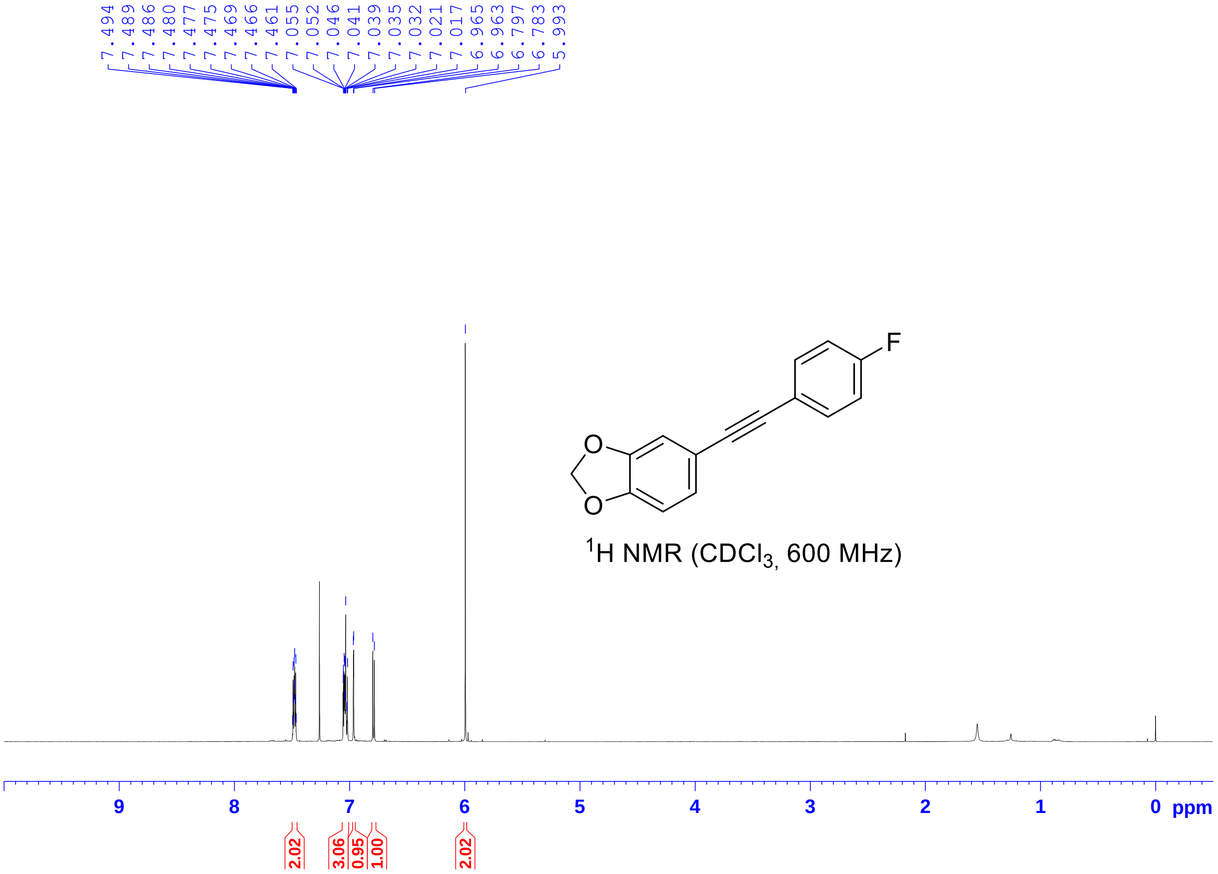

^{1}H NMR spectrum of 5-((4-fluorophenyl)ethynyl)benzo[d][1,3]dioxole (**1g**) in $CDCl_3$.

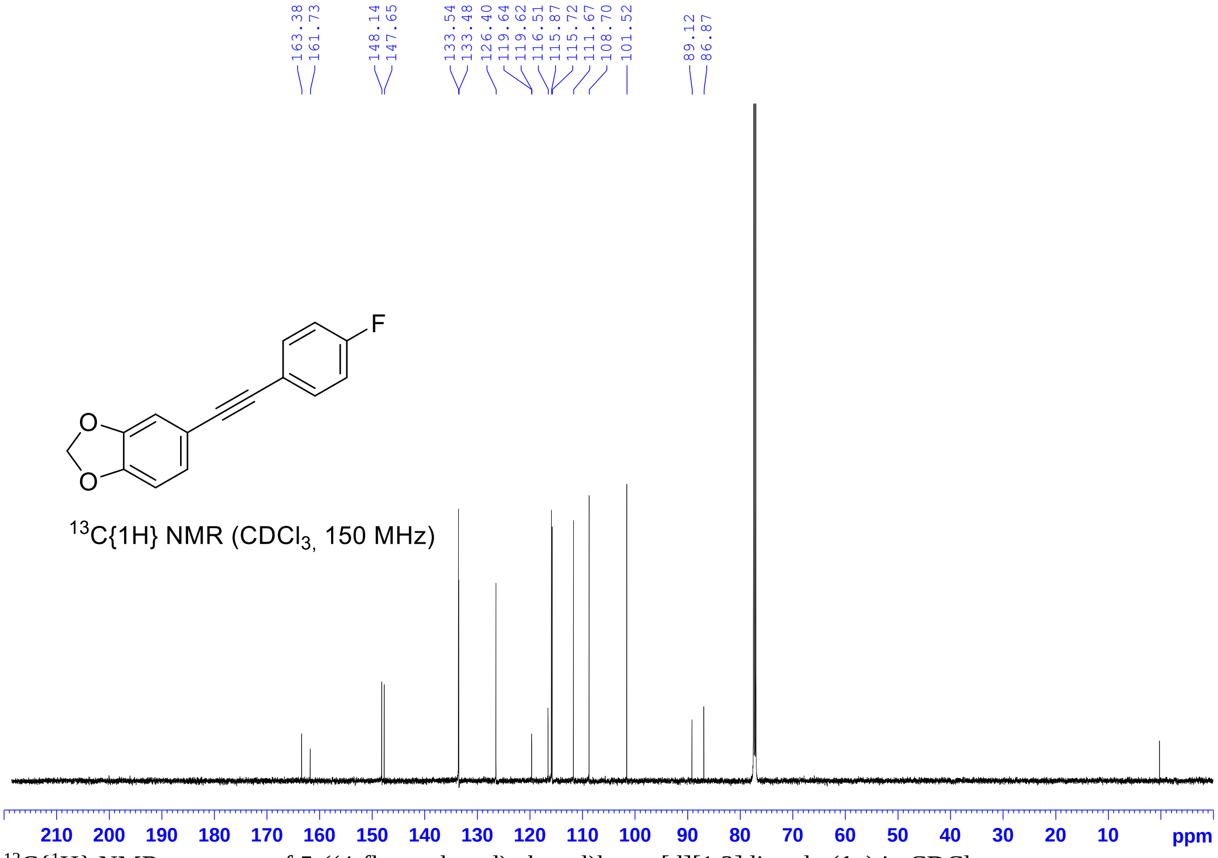

^{13}C{^{1}H} NMR spectrum of 5-((4-fluorophenyl)ethynyl)benzo[d][1,3]dioxole (**1g**) in $CDCl_3$.

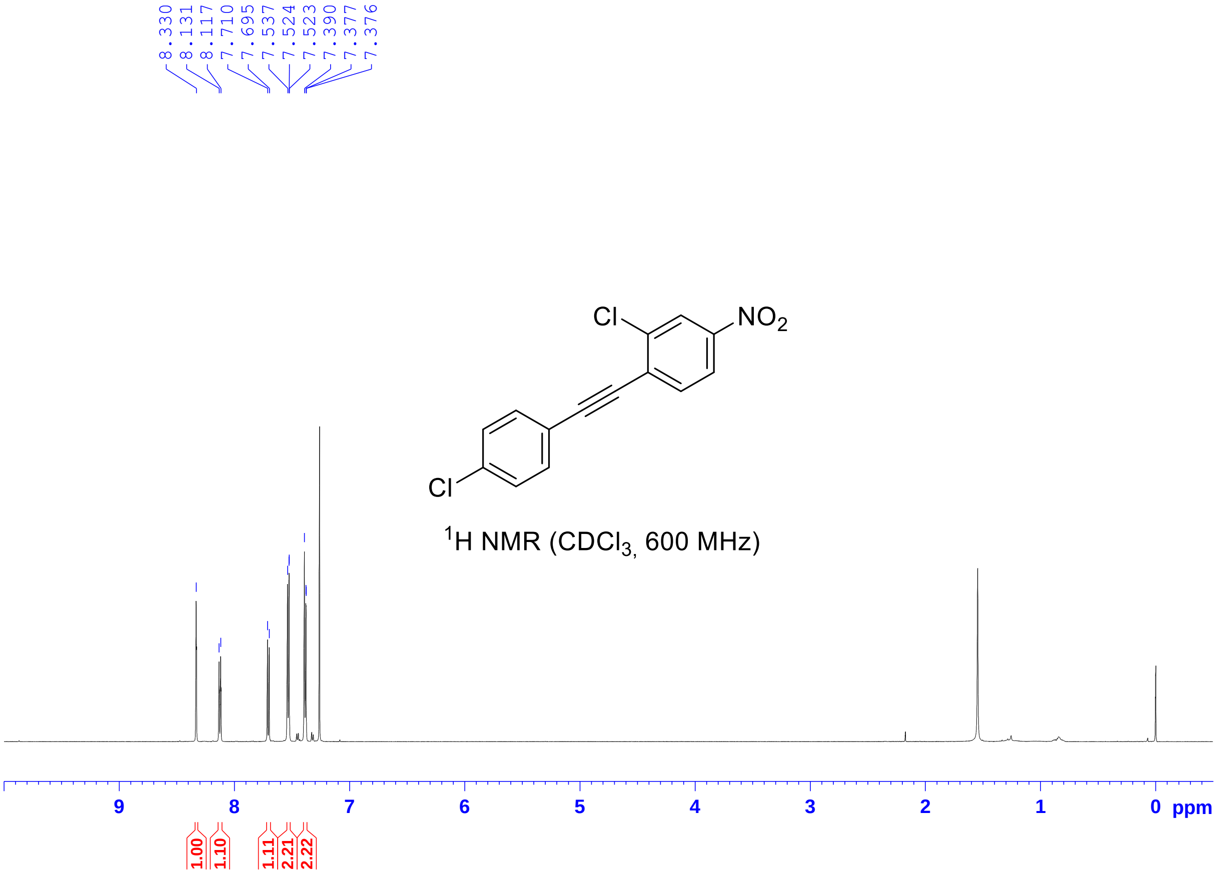

^{1}H NMR spectrum of 2-chloro-1-((4-chlorophenyl)ethynyl)-4-nitrobenzene (**1h**) in $CDCl_3$.

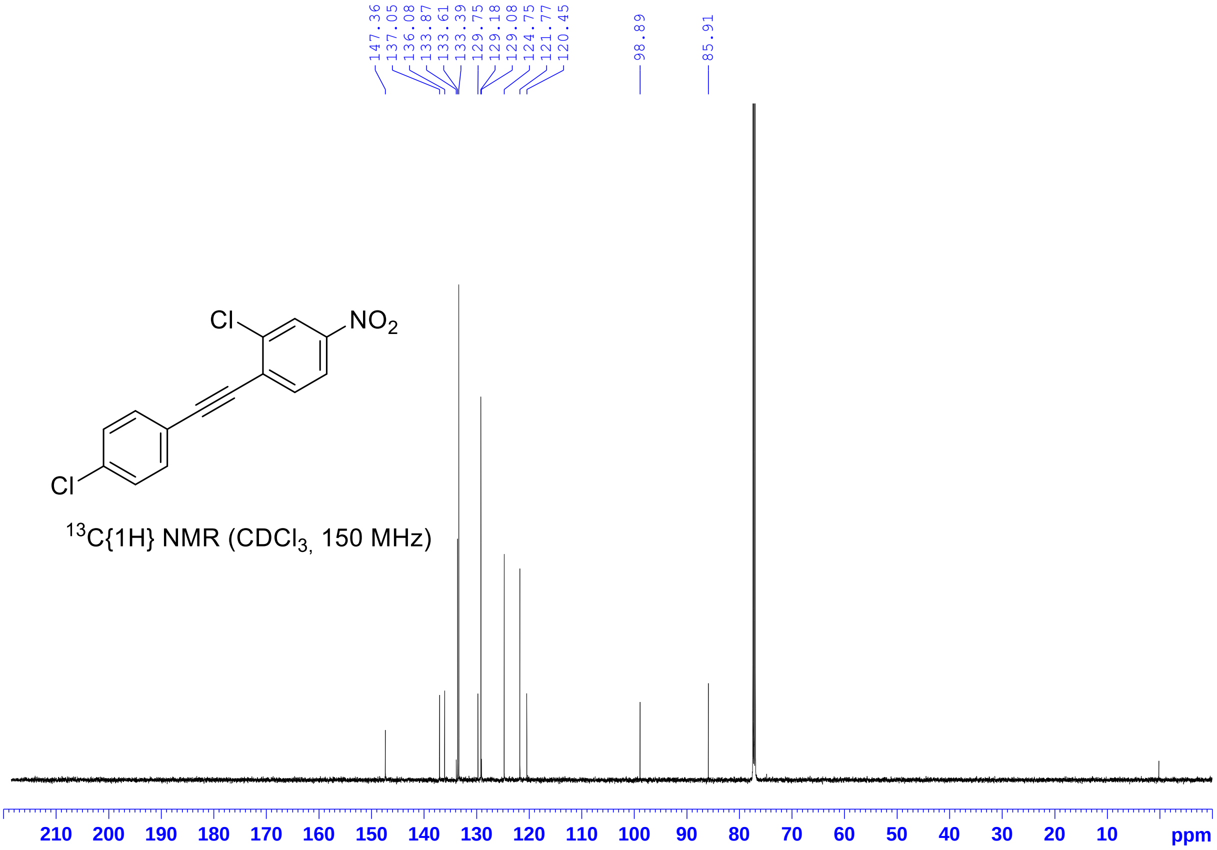

^{13}C{^{1}H} NMR spectrum of 2-chloro-1-((4-chlorophenyl)ethynyl)-4-nitrobenzene (**1h**) in $CDCl_3$.

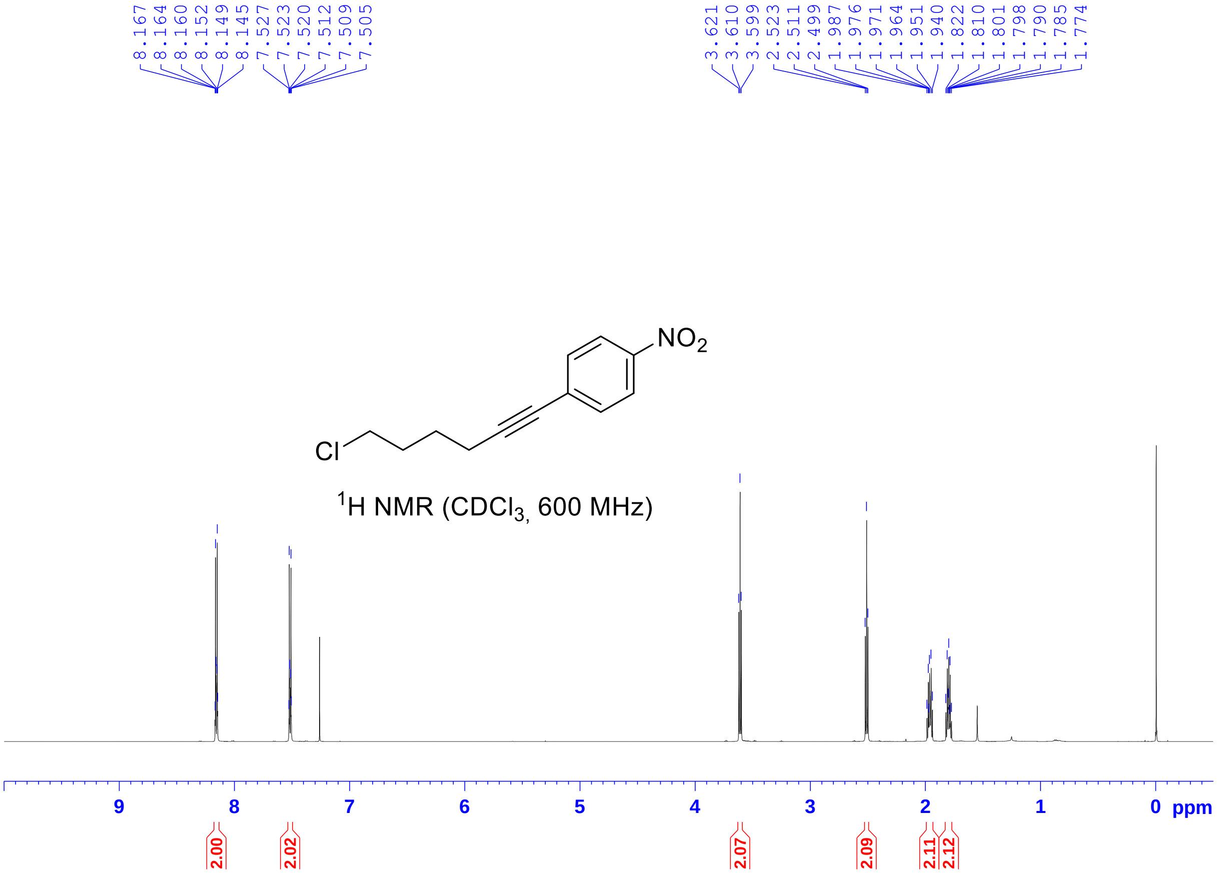

^{1}H NMR spectrum of 1-(6-chlorohex-1-yn-1-yl)-4-nitrobenzene (**1j**) in $CDCl_3$.

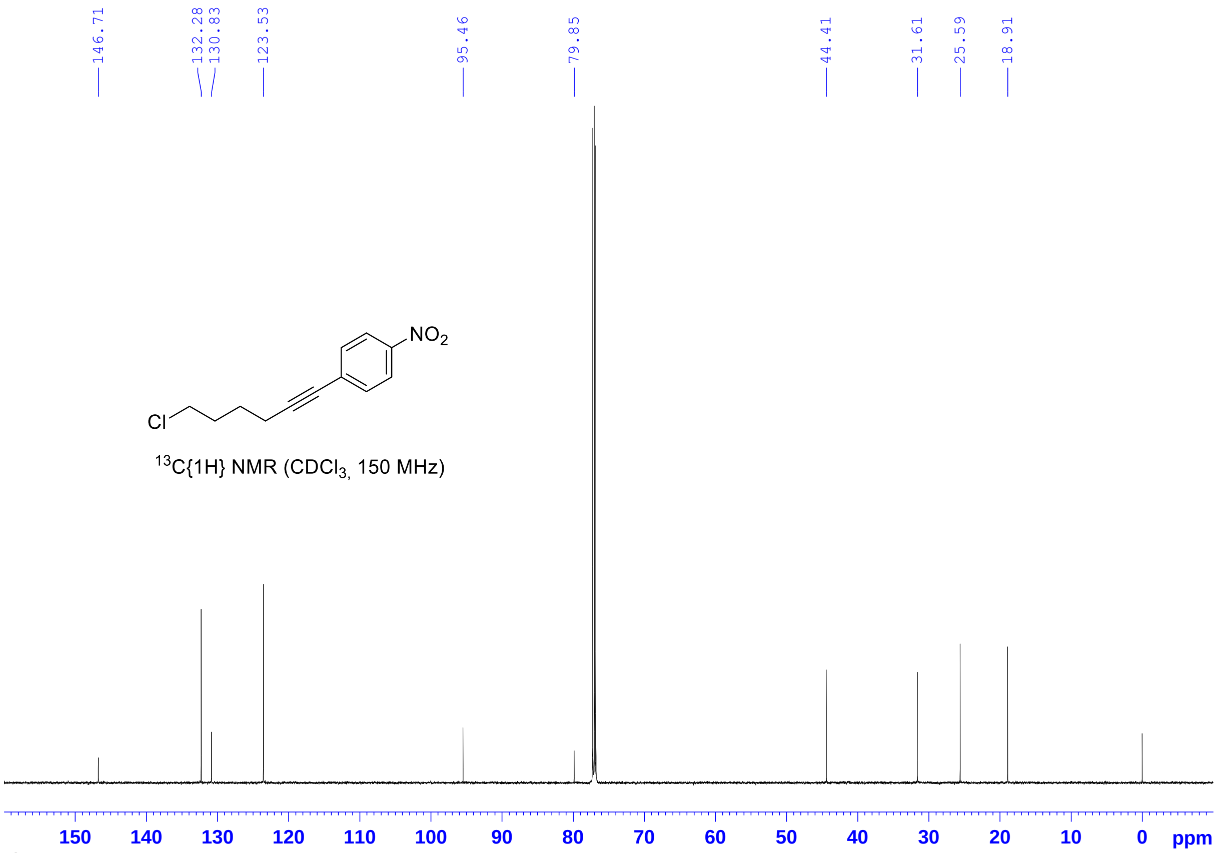

^{13}C{^{1}H} NMR spectrum of 1-(6-chlorohex-1-yn-1-yl)-4-nitrobenzene (**1j**) in $CDCl_3$.

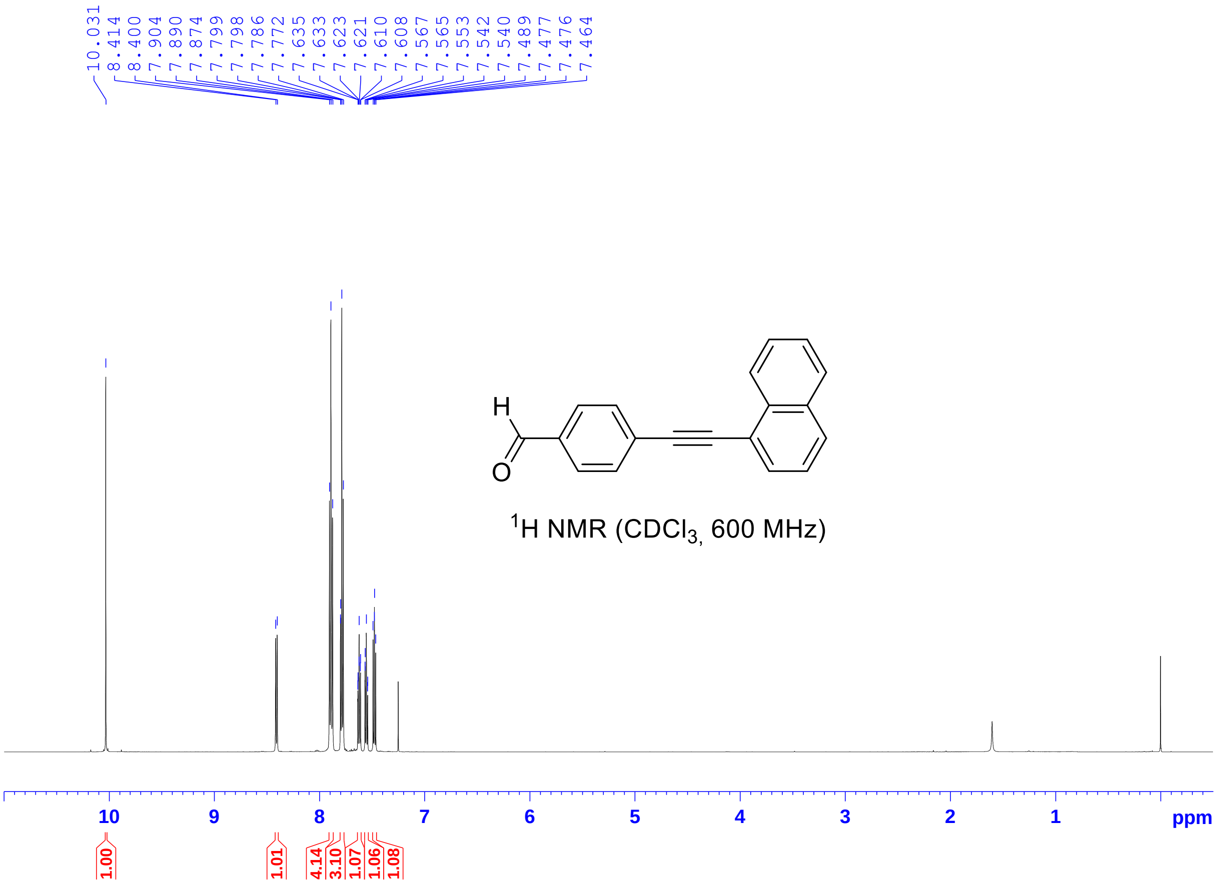

^{1}H NMR spectrum of 4-(naphthalen-1-ylethynyl)benzaldehyde (**1k**) in $CDCl_3$.

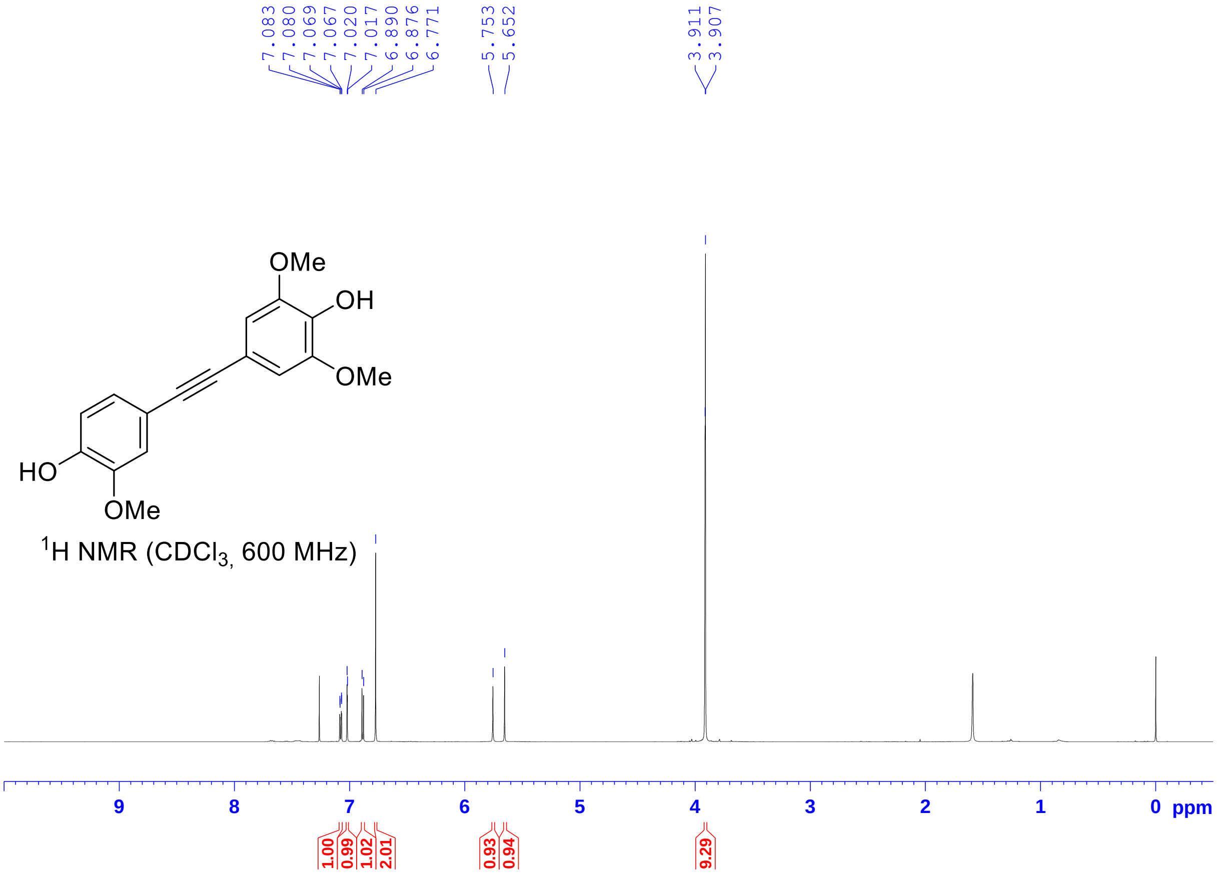

^{1}H NMR spectrum of 4-((4-hydroxy-3-methoxyphenyl)ethynyl)-2,6-dimethoxyphenol (**1n**) in $CDCl_3$.

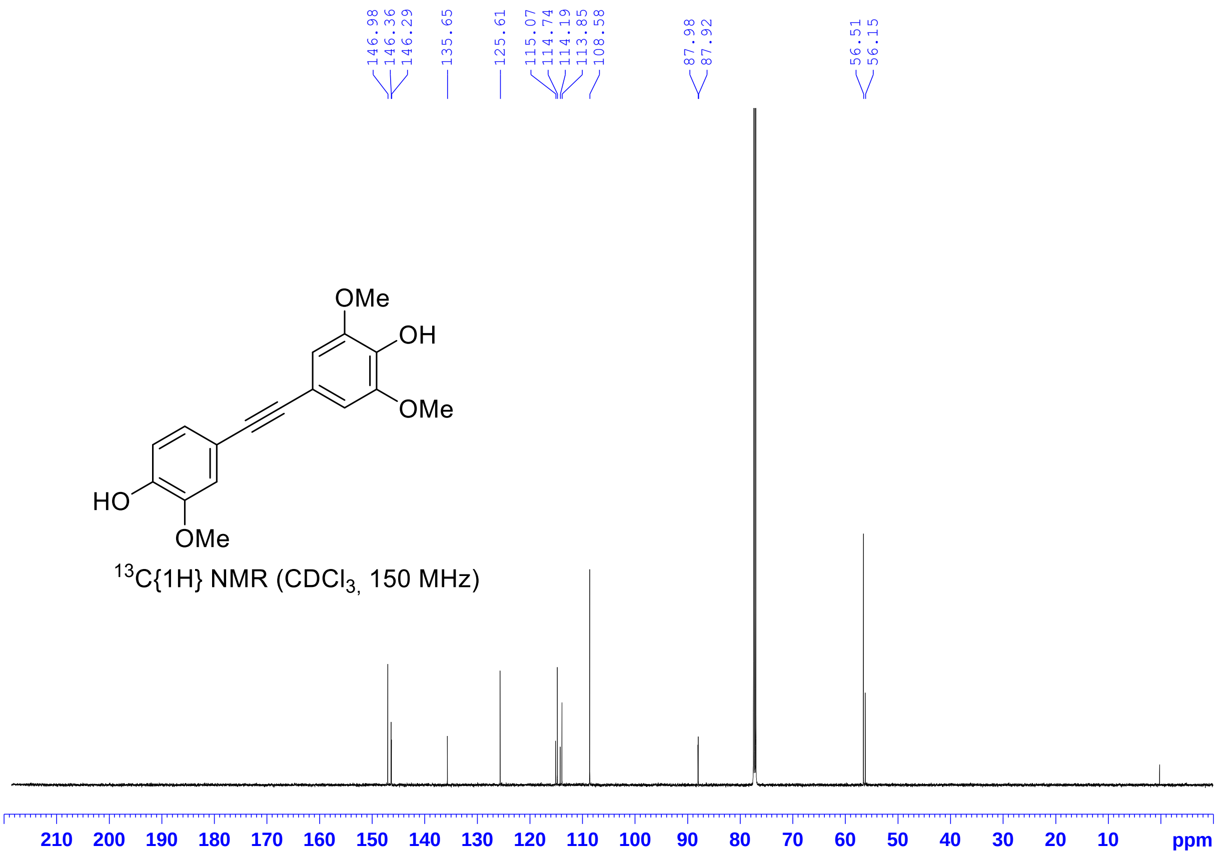

$^{13}C\{^{1}H\}$ NMR spectrum of 4-((4-hydroxy-3-methoxyphenyl)ethynyl)-2,6-dimethoxyphenol (**1n**) in $CDCl_3$.

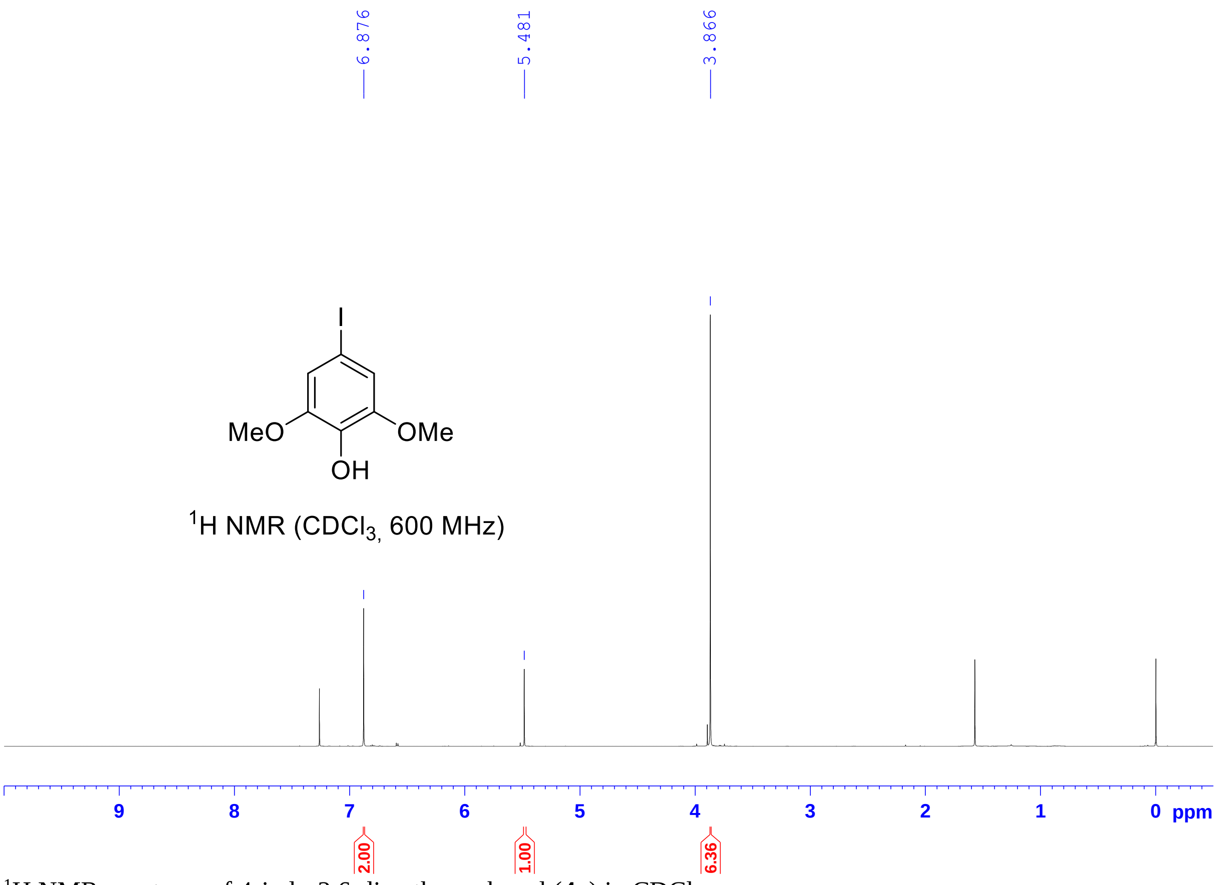

^{1}H NMR spectrum of 4-iodo-2,6-dimethoxyphenol (**4a**) in $CDCl_3$.

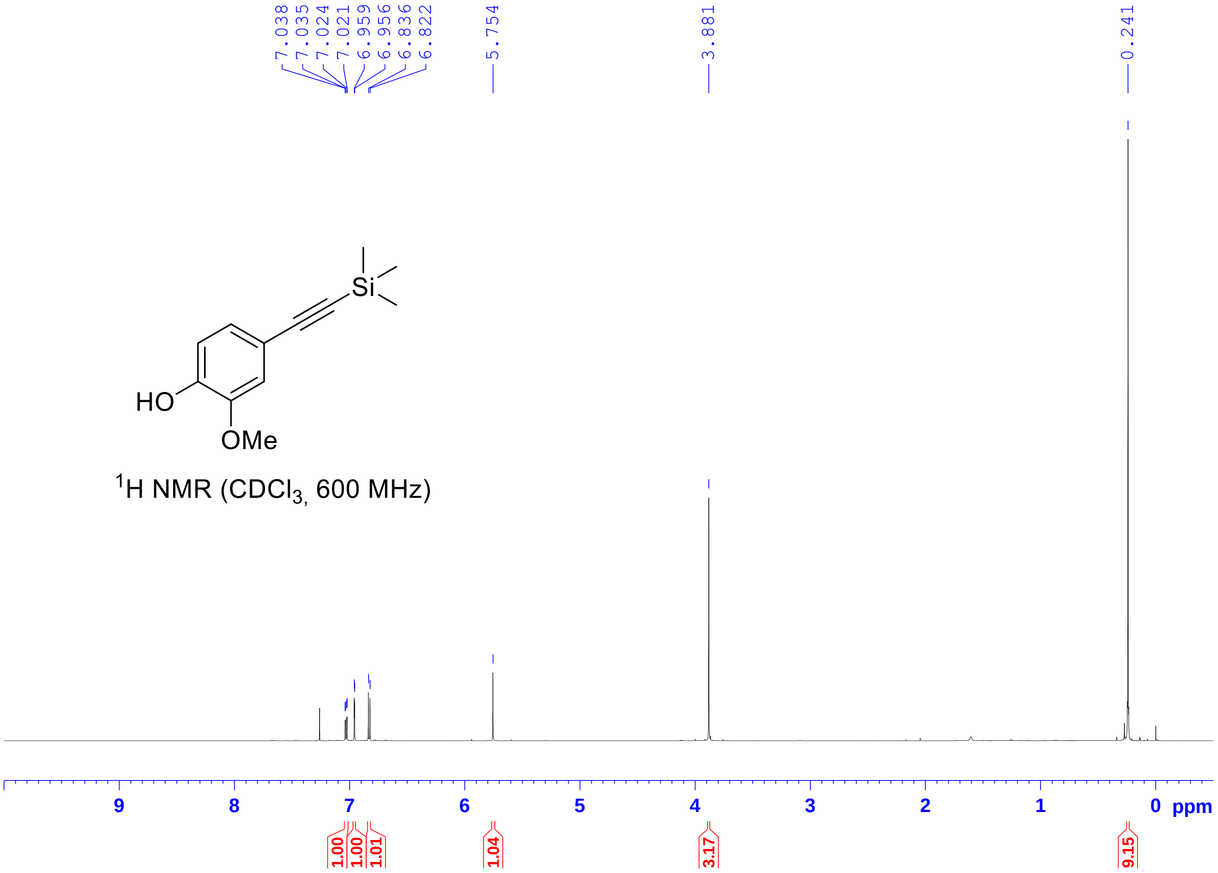

^{1}H NMR spectrum of 2-methoxy-4-((trimethylsilyl)ethynyl)phenol in $CDCl_3$.

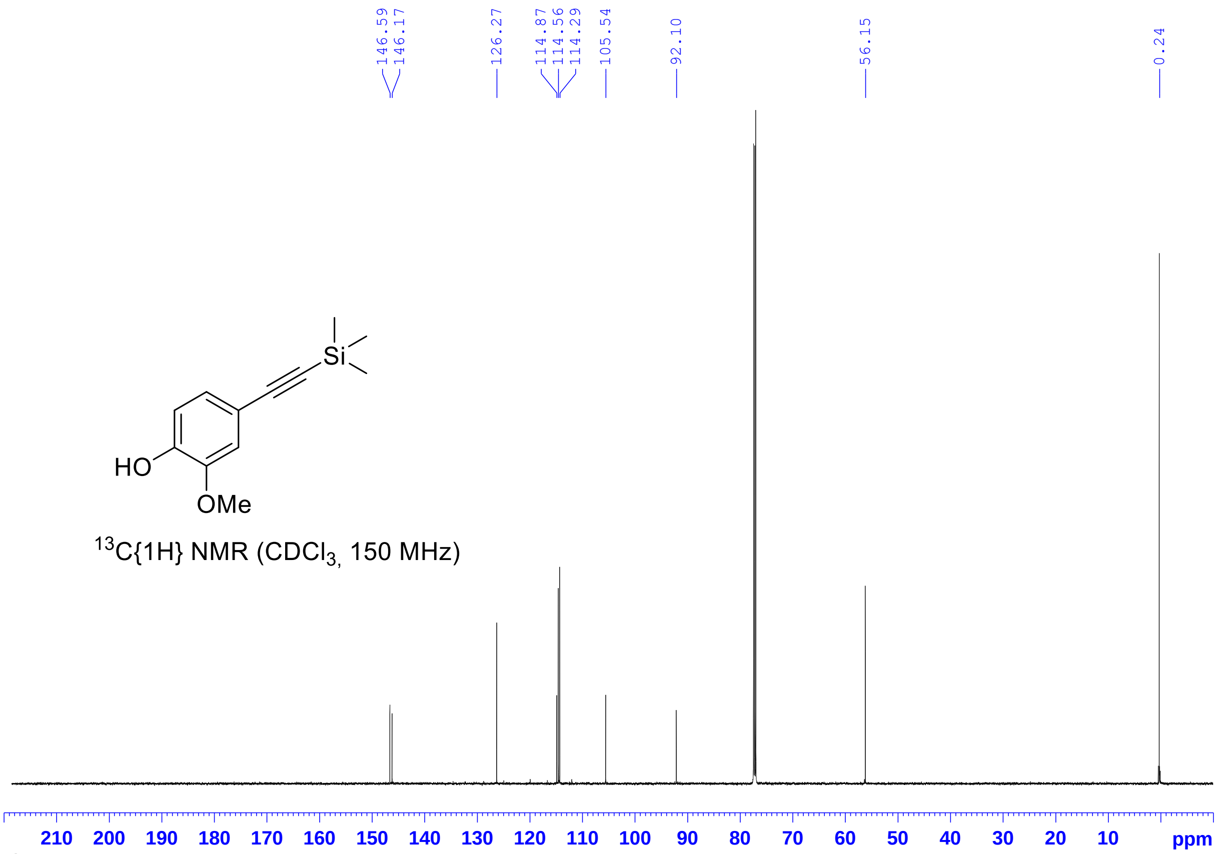

^{13}C{^{1}H} NMR spectrum of 2-methoxy-4-((trimethylsilyl)ethynyl)phenol in $CDCl_3$.

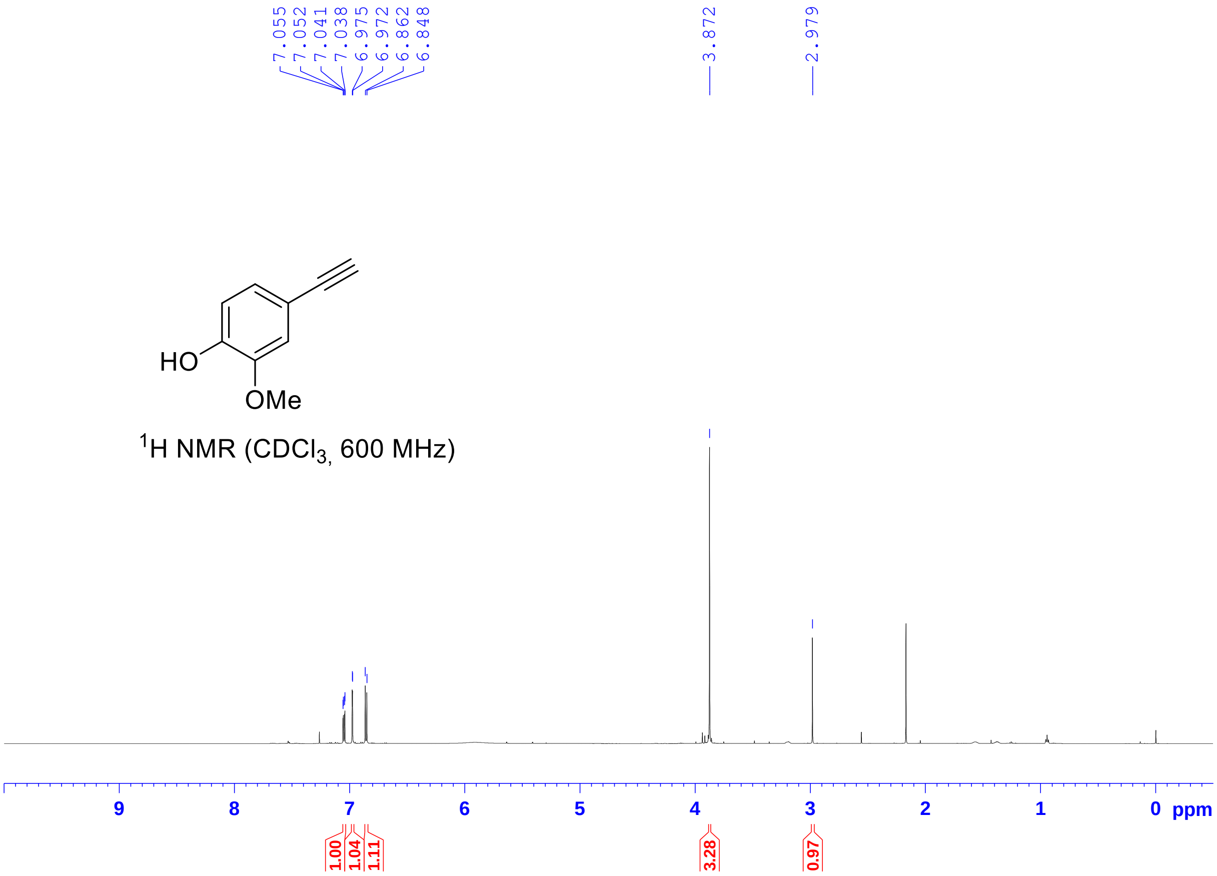

^{1}H NMR spectrum of 4-ethynyl-2-methoxyphenol (**3a**) in $CDCl_3$. Signal at 2.16 ppm is from acetone.

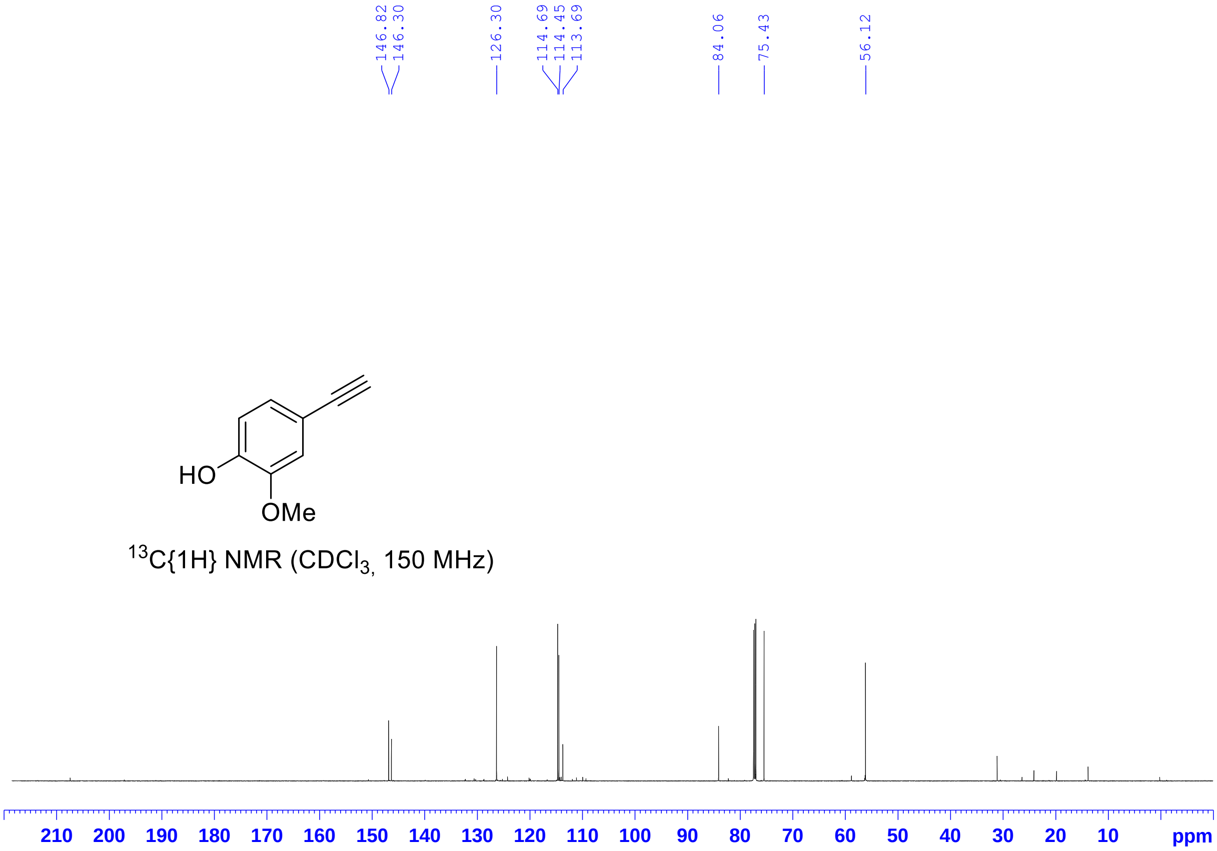

^{13}C{^{1}H} NMR spectrum of 4-ethynyl-2-methoxyphenol (**3a**) in $CDCl_3$. Signals from 35 to 10 ppm are from trace impurities.

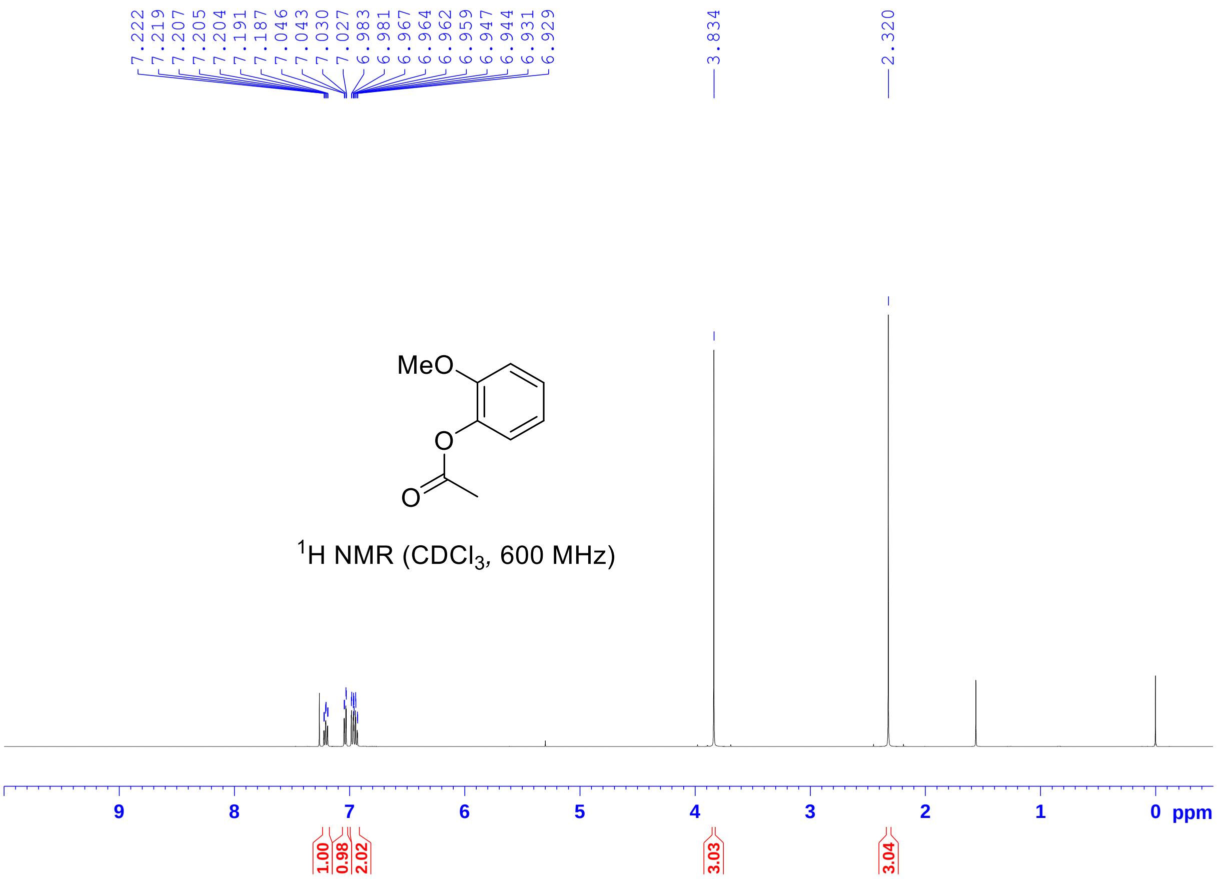

^{1}H NMR spectrum of 2-methoxyphenyl acetate in $CDCl_3$.

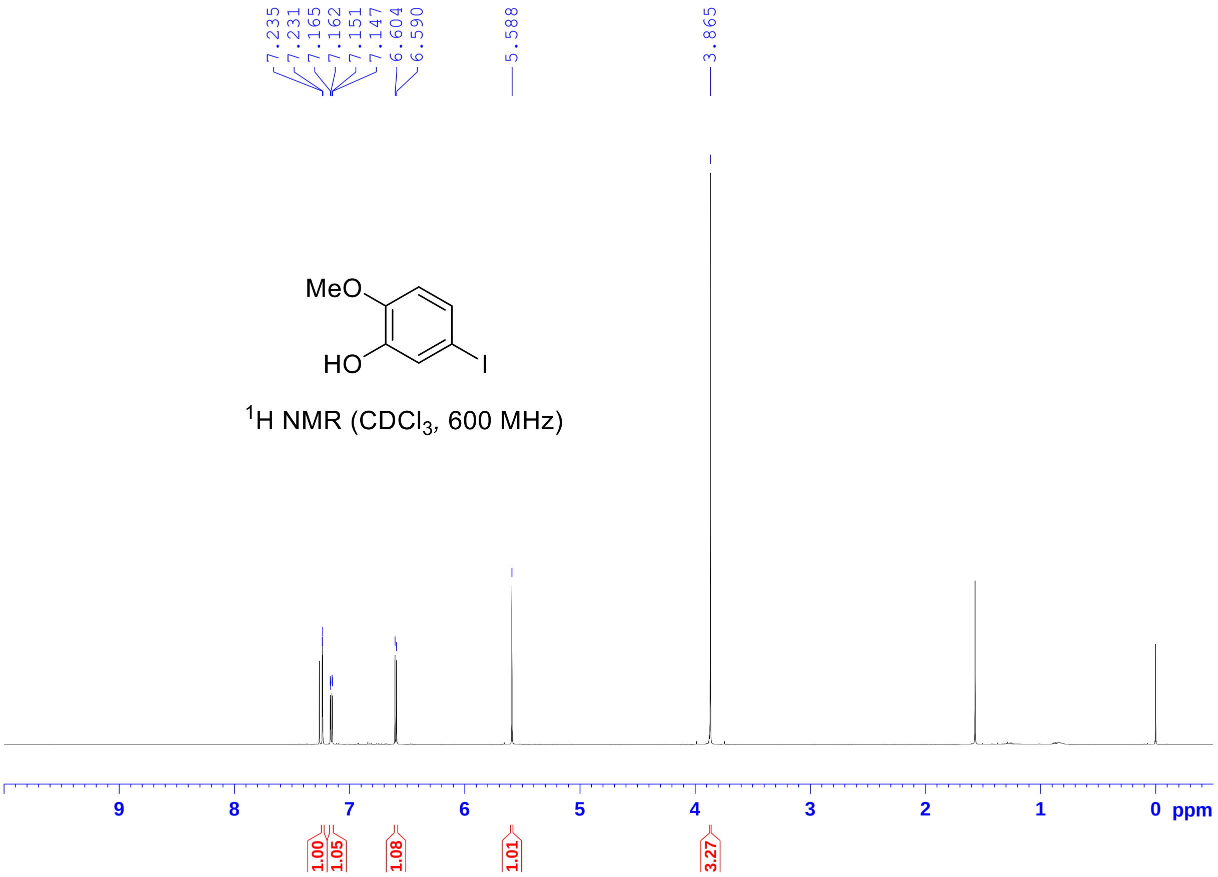

^{1}H NMR spectrum of 5-iodo-2-methoxyphenol (**4b**) in $CDCl_3$.

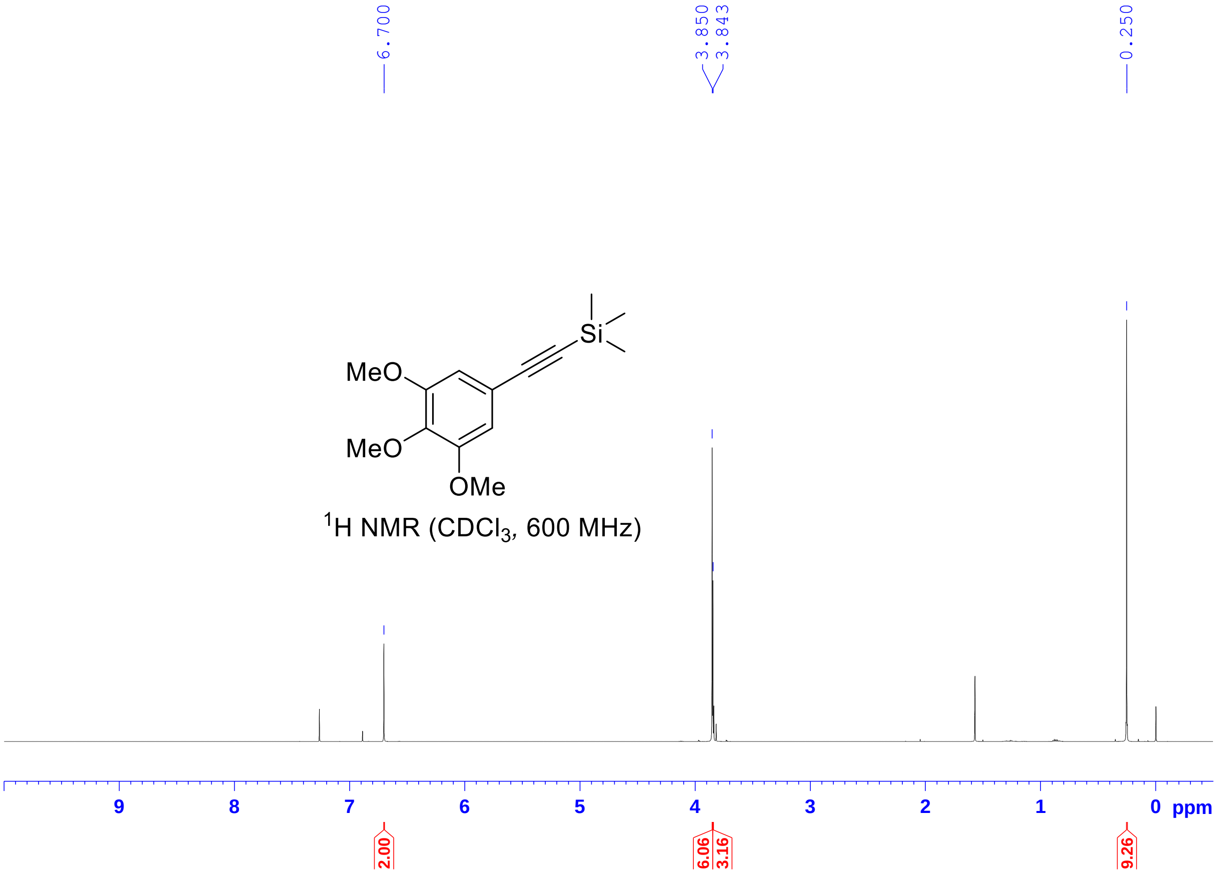

^{1}H NMR spectrum of trimethyl((3,4,5-trimethoxyphenyl)ethynyl)silane in $CDCl_3$.

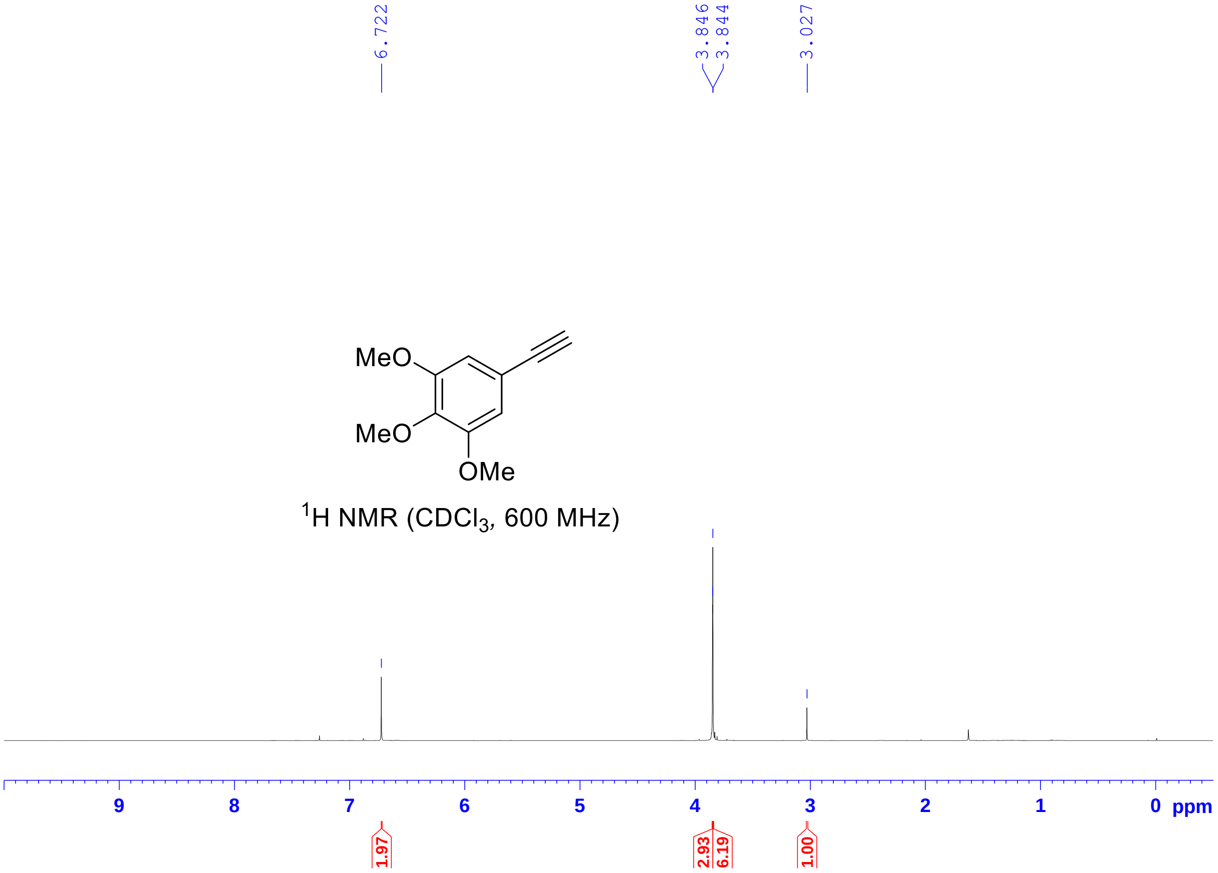

^{1}H NMR spectrum of 5-ethynyl-1,2,3-trimethoxybenzene (**3b**) in $CDCl_3$.

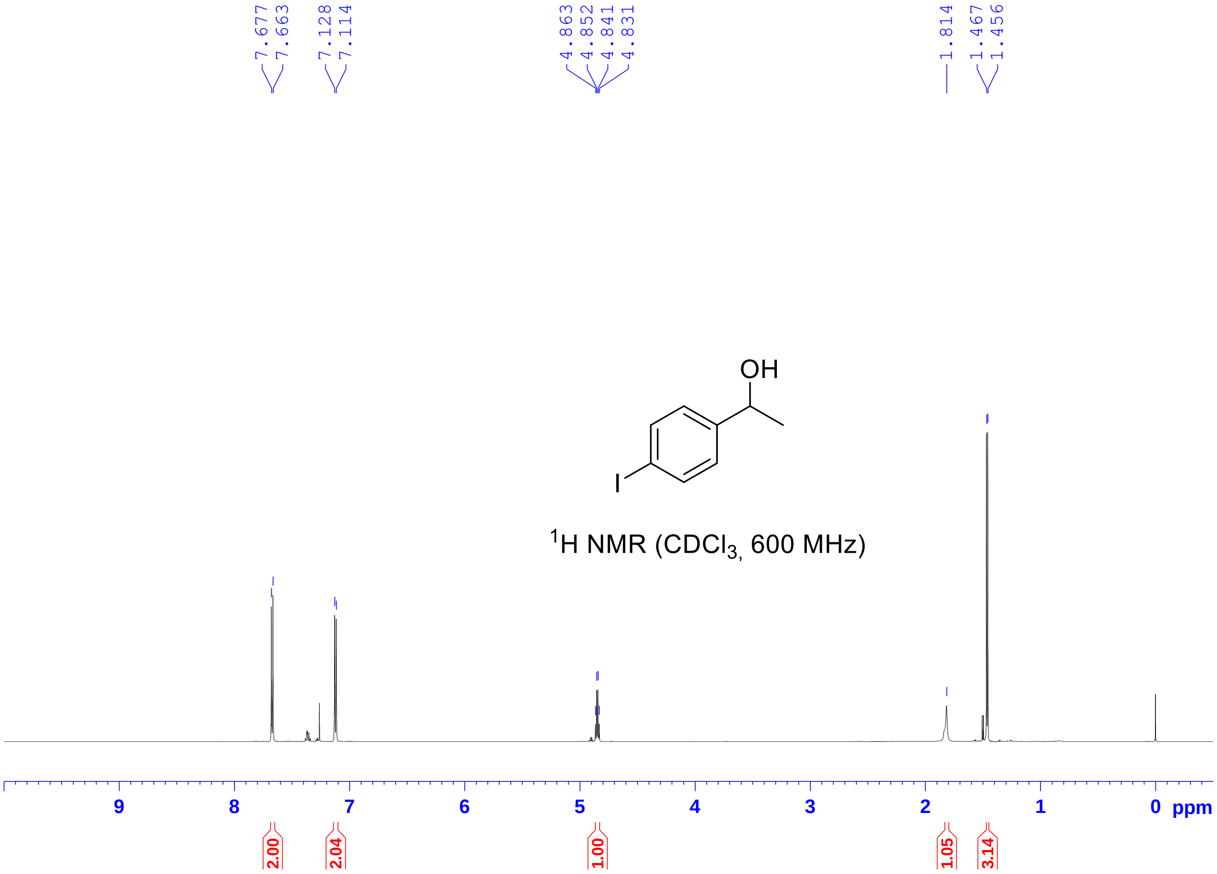

^{1}H NMR spectrum of 1-(4-iodophenyl)ethan-1-ol (**4c**) in $CDCl_3$.

4. REFERENCES

[1] Smith, A. J.; Young, A.; Rohrbach, S.; O'Connor, E. F.; Alison, M.; Wang, H.-S.; Poole, D. L.; Tuttle, T.; Murphy, J. A. Electron-Transfer and Hydride-Transfer Pathways in the Stoltz-Grubbs Reducing System (KO*t*Bu/Et_3SiH). *Angew. Chem., Int. Ed.* **2017**, *56*, 13747–13751.
[2] Kantam, M. L.; Chakravarti, R.; Chintareddy, V. R.; Sreedhar, B.; Bhargava, S. Palladium-Catalyzed Heck Coupling-Hydrogenation: Highly Efficient One-Pot Synthesis of Dibenzyls and Alkyl Phenyl Esters. *Adv. Synth. Catal.* **2008**, *350*, 2544–2550.
[3] Jin, L.; Qian, J.; Sun, N.; Hu, B.; Shen, Z. Hu, X. Pd-Catalyzed reductive heck reaction of olefins with aryl bromides for Csp^2-Csp^3 bond formation. *Chem. Commun.* **2018**, *54*, 5752–5755.
[4] Pandiri, H.; Gonnade, R. G.; Punji, B. Synthesis of quinolinyl-based pincer copper(II) complexes: an efficient catalyst system for Kumada coupling of alkyl chlorides and bromides with alkyl Grignar reagents. *Dalton Trans.* **2018**, *47*, 16747–16754.
[5] Yue, H.; Zhu, C.; Shen, L.; Geng, Q.; Hock, K. J.; Yuan, T.; Cavallo, L.; Rueping, M. Nickel-catalyzed C-N bond activation: activated primary amines as alkylationg reagents in reductive cross-coupling. *Chem. Sci.* **2019**, *10*, 4430–4435.
[6] Garapati, V. K. R.; Gravel, M. Oxazolium Salts as Organocatalysts for the Umpolung of Aldehydes. *Org. Lett.* **2018**, *20*, 6372–6375.
[7] Do, H.-Q.; Tran-Vu, H.; Daugulis, O. Copper-Catalyzed Homodimerization of Nitronates and Enolates under an Oxygen Atmosphere. *Organometallics,* **2012**, *31*, 7816–7818.
[8] Majumder, P. L.; Sen, R. C. Moscatilin, a bibenzyl derivative from the orchid *Dendrobium moscatum. Phytochemistry* **1987**, *26*, 2121–2124.
[9] Guo, L.; Ma, X.; Fang, H.; Jia, X.; Huang, Z. A General and Mild Catalytic α-Alkylation of Unactivated Esters Using Alcohols. *Angew. Chem., Int. Ed.* **2015**, *54*, 4023–4027.
[10] Yang, H. ; Han, X.; Li, G.; Wang, Y. *N*-Heterocyclic carbene palladium complex supported on ionic liquid-modified SBA-16: an efficient and highly recyclable catalyst for the Suzuki and Heck reactions. *Green Chem.* **2009**, *11*, 1184–1193.
[11] Peddikotla, P.; Chittiboyina, A. G.; Khan, I. A. Synthesis of Pterostilbene by Julia Olefination. *Synth. Commun.* **2013**, *43*, 3217–3223.
[12] Zou, Y.; Huang, Q.; Huang, T.; Ni, Q.; Zhang, E.; Xu, T.; Yuan, M.; Li, J. CuI/1,10-phen/PEG promoted decarboxylation of 2,3-diarylacrylic acids: synthesis of stilbenes under neutral and microwave conditions with an *in situ* generated recyclable catalyst. *Org. Biomol. Chem.* **2013**, *11*, 6967–6974.
[13] Zhu, X.; Liu, J.; Chen, T.; Su, W. Mechanically activated synthesis of (*E*)-stilbene derivatives by high-speed ball milling. *Appl. Organomet. Chem.* **2012**, *26*, 145–147.
[14] Ekebergh, A.; Begon, R.; Kann, N. Ruthenium-Catalyzed *E*-Selective Alkyne Semihydrogenation with Alcohols as Hydrogen Donors. *J. Org. Chem.* **2020**, *85*, 2966–2975.
[15] Lu, X.-L.; Shannon, M.; Peng, X.-S.; Wong, H. N. C. Stereospecific Iron-Catalyzed Carbon(sp^2)–Carbon(sp^3) Cross-Coupling with Alkyllithium and Alkenyl Iodides. *Org. Lett.* **2019**, *21*, 2546–2549.
[16] Gill, M. T.; Bajaj, R.; Chang, C. J.; Nichols, D. E.; McLaughlin, J. L. 3,3',5'-Tri-O-methylpiceatannol and 4,3',5'-tri-O-methylpiceatannol: Improvements over piceatannol in bioactivity. *J. Nat. Prod.* **1987**, *50*, 36–40.
[17] Peng, Z.-Y.; Ma, F.-F.; Zhu, L.-F.; Xie, X.-M.; Zhang, Z. Lewis Acid Promoted Carbon–Carbon Double-Bond Formation via Organozinc Reagents and Carbonyl Compounds. *J. Org. Chem.* **2009**, *74*, 6855–6858.
[18] Garg, N.; Paira, S.; Sundararaju, B. Efficient Transfer Hydrogenation of Ketones using Methanol as Liquid Organic Hydrogen Carrier. *ChemCatChem* **2020**, *12*, 3472–3476.
[19] Gaukroger, K.; Hadfield, J. A.; Hepworth, L. A.; Lawrence, N. J.; McGown, A. T. Novel Syntheses of Cis and Trans Isomers of Combretastatin A-4. *J. Org. Chem.* **2001**, *66*, 8135–8138.
[20] Fu, S.; Chen, N.-Y.; Liu, X.; Shao, Z.; Luo, S.-P.; Liu, Q. Ligand-Controlled Cob alt-Catalyzed Transfer Hydrogenation of Alkynes: Stereodivergent Synthesis of *Z*- and *E*-Alkenes. *J. Am. Chem. Soc.* **2016**, *138*, 8588–8594.
[21] Sonogashira, K.; Tohada, Y.; Hagihara, N. A convenient synthesis of acetylenes: catalytic substitutions of acetylenic hydrogen with bromoalkenes, iodoarenes and bromopyridines. *Tetraherdon Lett.* **1975**, *16*, 4467–4470.
[22] Hamasaka, G.; Roy, D.; Tazawa, A.; Uozumi, Y. Arylation of Terminal Alkynes by Aryl Iodides Catalyzed by a Parts-per-Million Loading of Palladium Acetate. *ACS Catal.* **2019**, *9*, 11640–11646.
[23] Watanabe, K.; Mino, T.; Ishikawa, E.; Okano, M.; Ikematsu, T.; Yoshida, Y.; Sakamoto, M.; Sato, K.; Yoshida, K. Synthesis of *o*-Allyloxy(ethynyl)benzene Derivatives by Cu-Catalyzed Suzuki-Miyaura-Type Reaction and Their Transformations into Heterocyclic Compounds. *Eur. J. Org.* **2017**, *16*, 2359–2368.
[24] Chandra, R.; Oya, S.; Kung, M.-P.; Hou, C.; Jin, L.-W.; Kung, H. F. New Diphenylacetylenes as Probes for Positron Emission Tomographic Imaging of Amyloid Plaques. *J. Med. Chem.* **2007**, *50*, 2415–2423.

[25] Tang, S.; Liu, Y.; Gao, X.; Wang, P.; Huang, P.; Lei, A. Multi-Metal-Catalyzed Oxidative Radical Alkynylation with Terminal Alkynes: A New Strategy for C(sp^3)–C(sp) Bond Formation. *J. Am. Chem. Soc.* **2018**, *140*, 6006–6013.
[26] Mase, N.; Ando, T.; Shibagaki, F.; Sugita, A.; Narumi, T.; Toda, M.; Watanabe, N.; Tanaka, F. Fluorogenic aldehydes bearing arylethynyl groups: turn-on aldol reaction sensors for evaluation of organocatalysis in DMSO. *Tetrahedron Lett.* **2014**, *55*, 1946–1948.
[27] Luong, T. M.; Pilkington, L. I.; Barker, D. Stereoselective Total Synthesis of (+)-Aristolactam GI. *J. Org. Chem.* **2019**, *84*, 5747–5756.
[28] Bi, J.; Wu, H.; Zhang, Z.; Zhang, A.; Yang, H.; Feng, Y.; Fang, Y.; Zhang, L.; Wang, Z.; Qu, W.; Liu, F.; Zhang, C. Highly ordered columnar superlattice nanostructures with improved charge carrier mobility by thermotropic self-assembly of triphenylene-based discotics. *J. Mater. Chem. C* **2019**, *7*, 12463–12469.
[29] Kale, T. S.; Tovar, J. D. Regulation of peptide-π-peptide nanostructure bundling: the impact of 'cruciform' π-electron segments. *Tetrahedron* **2016**, *72*, 6084–6090.
[30] Zha, G.-F.; Fang, W.-Y.; Li, Y.-G.; Leng, J.; Chen, X.; Qin, H.-L. SO_2F_2-Mediated Oxidative Dehydrogenation and Dehydration of Alcohols to Alkynes. *J. Am. Chem. Soc.* **2018**, *140*, 17666–17673.
[31] Goseki, R.; Koizumi, T.; Kurakake, R.; Uchida, S.; Ishizone, T. Living Anionic Polymerization of 4-Halostyrenes. *Macromolecules* **2021**, *54*, 1489–1498.
[32] Saito, T.; Nishimoto, Y.; Yasuda, M.; Baba, A. Direct Coupling Reaction between Alcohols and Silyl Compounds: Enhancement of Lewis Acidity of Me_3SiBr Using $InCl_3$. *J. Org. Chem.* **2006**, *71*, 8516–8522.
